# Testicular SIRT1 Loss Reveals an Aging‐Like Proteomic Landscape and Precipitates Reproductive Deterioration

**DOI:** 10.1111/andr.70201

**Published:** 2026-03-12

**Authors:** María Iniesta‐Cuerda, Jiřina Havránková, Hedvika Řimnáčová, Jiří Moravec, František Liška, Martin Knytl, Milena Králíčková, Jan Nevoral

**Affiliations:** ^1^ Biomedical Center Faculty of Medicine in Pilsen Charles University Pilsen Czech Republic; ^2^ Department of Histology and Embryology Faculty of Medicine in Pilsen Charles University Pilsen Czech Republic; ^3^ First Faculty of Medicine Charles University Prague Czech Republic; ^4^ Department of Biology McMaster University Hamilton Ontario Canada; ^5^ Department of Cell Biology Faculty of Science Charles University Prague Czech Republic

**Keywords:** paternal age‐related subfertility, proteomic remodeling, SIRT1 insufficiency

## Abstract

**Background:**

Advanced paternal age is associated with reduced male fertility and testicular dysfunction. Among the molecular regulators involved in aging, SIRT1, a NAD^+^‐dependent deacetylase, plays a pivotal role in maintaining oxidative balance and cellular homeostasis. Although the age‐associated decline in SIRT1 levels is well established, the extent to which this reduction underlies testicular dysfunction and the specific proteomic alterations linked to it remain to be elucidated.

**Objective:**

This study aimed to determine whether testicular SIRT1 insufficiency contributes to testicular aging by promoting changes in the proteomic landscape and impairing male reproductive function.

**Materials and Methods:**

We employed a *Sirt1* ^+^/^−^ mouse model that mimics the partial SIRT1 decline observed during aging. Comparative analyses were conducted across wild type, aged wild type, and *Sirt1* ^+^/^−^ males. We assessed reproductive performance, testicular histology, sperm quality, and embryo development. In parallel, we performed proteomic profiling of testicular tissue to identify molecular pathways affected by aging and SIRT1 insufficiency.

**Results:**

*Sirt1* ^+^/^−^ males exhibited marked reproductive impairments, including reduced fertility, compromised embryo development, sperm morphological abnormalities, and increased testicular tubule degeneration. Proteomic analysis revealed substantial remodeling in both aged and *Sirt1* ^+^/^−^ testes, with overlapping alterations affecting proteins involved in oxidative stress responses, proteostasis, and chromatin regulation. Moreover, several proteins with recognized anti‐aging functions were undetectable in both aged and *Sirt1* ^+^/^−^ models yet consistently expressed in wild‐type testes. This deregulation reinforces the notion that testicular SIRT1 insufficiency recapitulates key features of the aging testis.

**Discussion:**

Our findings indicate that partial loss of SIRT1 is sufficient to trigger proteomic and functional hallmarks of testicular aging. In particular, we identified specific proteomic signatures linked to subfertility, including the loss of key capacitation‐related proteins regulated by SIRT1 in the testis, a pattern also observed in aged animals, which may represent a mechanistic model of SIRT1‐governed fertilization failure.

## 1 Introduction

In recent decades, profound demographic and sociocultural shifts, including increased life expectancy and delayed life milestones, have contributed to a significant postponement of parenthood in many societies. By 2022, the average paternal and maternal ages in the United States rose to 38.0 and 36.0 years [1], respectively, marking a significant increase compared to the paternal age of 27.4 years in 1972 and 30.9 years in 2015 [2]. In Europe, a similar trend was observed, with the average paternal age reaching 35.5 years in Italy by 2018 and 34.6 years in Germany by 2019 [3]. Although the impact of maternal age on fertility has long been recognized as a critical factor in reproductive planning, the contribution of paternal age has traditionally received less attention. However, accumulating clinical evidence now clearly implicates advanced paternal age in a range of adverse reproductive outcomes, including reduced testicular function [4, 5], fecundity [6], and embryo development [7, 8], prolonged time to pregnancy, and elevated risks of miscarriage, birth defects, and offspring diseases [9, 10], and even stillbirth [11]. These findings underscore the urgent need to elucidate the biological underpinnings of male reproductive aging and to identify the molecular pathways that drive age‐associated subfertility.

From a physiological perspective, aging in the male reproductive system is accompanied by progressive alterations in testicular structure and function [4]. With age, testicular volume and vascularization decline [12], accompanied by a reduction in the number and function of Leydig and Sertoli cells, leading to decreased testosterone production, impaired spermatogonia development, and reduced efficiency of spermatogenesis [12]. Consequently, daily sperm output is diminished [13, 14, 15]. Although men maintain spermatogenic capacity throughout life, advanced age is associated with an increased proportion of morphologically abnormal spermatozoa and subtle yet functionally relevant defects in sperm motility, chromatin integrity, and fertilization competence [4, 5, 15, 16]. Given these changes, a progressive decline in reproductive performance of older males becomes an expected outcome of aging, as cumulative alterations in testicular architecture, spermatogenic efficiency, and sperm quality converge to diminish male reproductive potential [15, 17, 18].

High‐throughput proteomic profiling has emerged as a powerful approach to investigate the molecular basis of testicular aging, offering comprehensive insight into global alterations in protein expression, post‐translational modifications, and regulatory networks. Among the molecular candidates increasingly implicated in reproductive aging, the NAD^+^‐dependent deacetylase SIRT1 has attracted significant interest because it is abundantly expressed in the testis, particularly in spermatogonia, Sertoli cells, and round spermatids [19, 20, 21], and its expression declines with age [21]. This suggests a potential causal link between reduced SIRT1 activity and testicular dysfunction. Previous studies have shown that complete *Sirt1* ablation results in profound testicular atrophy, defective spermatogenesis, and marked subfertility [20, 21, 22, 23, 24, 25]. Nevertheless, the extent to which more subtle, physiologically relevant reductions in SIRT1, such as those occurring during aging, contribute to testicular deterioration remains non defined, and their impact on the proteomic architecture of the aging testis is yet to be fully characterized.

In this study, the aim was double: first, to characterize the reproductive phenotype of animals experiencing testicular SIRT1 insufficiency rather than the total ablation of this protein, as partial insufficiency more closely reflects physiological conditions; and second, to compare these findings with the functional features of testicular aging. Using a *Sirt1* ^+/−^ mouse model and comparative proteomic profiling of young, aged, and SIRT1‐insufficient testes, we identified shared alterations in protein expression and pathway disruption. Our results showed that the reproductive phenotype of animals experiencing testicular SIRT1 insufficiency largely remains that of aged animals. Interestingly, similar proteomic signatures were observed in aged animals and those with testicular SIRT1 insufficiency, revealing molecular alterations driven by SIRT1 deficiency that may also underlie age‐associated testicular decline.

## 2 Materials and Methods

### 2.1 Chemicals and Reagents

All chemicals and reagents used for the experiments were purchased from Merck Millipore or Thermo Fischer Scientific, unless otherwise stated.

### 2.2 Animals

Mice used in the experiments were euthanized by cervical dislocation, in accordance with Act No. 246/1992, on the Protection of Animals against Cruelty, under supervision of the Animal Welfare Advisory Committee at the Charles University, Faculty of Medicine in Pilsen, and approved by the Animal Welfare Advisory Committee at the Ministry of Education, Youth and Sports of the Czech Republic (Ethics approval number: MSMT‐33798/2021‐4). The euthanasia method used in this study consists of CO_2_ narcosis followed by cervical dislocation. Anesthesia is not required for any of the experiments described. This work has been reported in accordance with the ARRIVE 2.0 guidelines. All mice used in the experiments were housed in cages at 21°C ± 2°C, under a 12:12 h light/dark cycle, with ad libitum access to food and water. The *Sirt1* ^+/−^ experimental males were generated as described earlier [26]. Briefly, *Sirt1* conditional knock‐out (Zp3‐Cre:*Sirt1*loxPloxP) females, with excised exon 5–7 (Sirt1^Δ5–7^) in oocytes, were mated with wild‐type (WT) C57Bl/6N males. F1 males were used for back‐cross with WT females, producing *Sirt1* ^+/−^ experimental males and *Sirt1* ^+/+^ siblings that were used as WT control. The 3.5‐month‐old WT and *Sirt1* ^+/−^ males were used in our experiments. For the evaluation of the aging effects on male reproductive performance, WT animals reaching 12 months were considered aged (Old WT).

### 2.3 Sample Collection of Testicular Tissue and Sperm Isolation

Testis and spermatozoa of *Sirt1* ^+/−^ and their WT siblings were sampled. After euthanasia, the body weight was recorded, and the testes were dissected and weighed. Tunica albuginea was removed from testis, and testicular lysate was prepared in RIPA buffer. Another testis was fixed in Bouin solution and used for histological assessment. For sperm isolation, epididymis was extracted in 0.5 mL Human Tubal Fluid medium (NaCl 101.6 mM; KCl 4.7 mM; KH_2_PO_4_ 0.4 mM; CaCl2·2H_2_O 2.0 mM; MgSO_4_·7H_2_O; NaHCO_3_ 25.0 mM; Na Lactate 40.6 mM), and spermatozoa were allowed to swim out for 15 min. Thereafter, both testicular tissue and spermatozoa were processed in accordance with the evaluations conducted.

### 2.4 Fertility Trial

Sexually mature 8‐ to 12‐week‐old WT females were mated overnight with either WT or *Sirt1* ^+/−^ males, and presumptive zygotes were collected the following day. Both hormonally stimulated and non‐stimulated females were used. For hormonal stimulation, pregnant mare serum gonadotropin was administered, followed by human chorionic gonadotropin (hCG) 48 h later. At 15–16 h post‐hCG injection, presumptive zygotes were recovered from the oviducts by flushing the fallopian tubes. One‐cell stage embryos were then isolated and cultured in EmbryoMax KSOM medium (MR‐121‐D; Millipore, USA; 0.1% w/v bovine serum albumin [BSA]) until the blastocyst stage (4 days post‐coitum). Several parameters were recorded at different times of embryo development. Cleavage rate referred to the percentage of zygotes reaching the 2‐cell stage (2 days post‐coitum), whereas blastocyst rate indicated the percentage of 2‐cell embryos that progressed to the blastocyst stage (4 days post‐coitum). Pregnancy rate was defined as the percentage of females from which at least one zygote was successfully recovered after natural mating and oviductal flushing.

### 2.5 Embryo Quality Assessment by Immunocytochemistry (ICC)

Embryo proteins were assessed via slightly modified ICC protocols [27]. Embryos were fixed, permeabilized, and blocked with PBS supplemented with paraformaldehyde (4% (w/v)), Triton‐X 100 (0.1% (v/v)), Tween‐20 (0.2% (v/v)), or BSA (5% (w/v)) before incubation with primary antibodies (mouse anti‐Cdx2 (bs1620R, Bioss) and rabbit anti‐Oct4 (ab217250, Abcam)). Secondary antibodies were used for visualization (anti‐mouse and anti‐rabbit Alexa Fluor 488 and 647), and non‐specific binding was tested by omitting primary antibodies in control samples. Stained embryos were mounted in Vectashield with DAPI, and then imaging was performed using an Olympus IX83 spinning disc confocal microscope. For blastocysts, Cdx2‐ and Oct4‐positive cells were recorded for trophectoderm (TE) and inner cell mass (ICM) evaluation, respectively.

### 2.6 Evaluation of Sperm Parameters

Several functional parameters of spermatozoa were assessed after swim‐out from the epididymis. Sperm concentration and motility were evaluated as previously described [28], whereas morphology was analyzed using SpermBlue staining (Microptic, Barcelona) following the manufacturer's instructions. A total of 100 spermatozoa per sample were examined, and morphological abnormalities, such as round or elongated heads, multiple heads or tails, and shortened tails, were recorded. Acrosome integrity and membrane integrity were evaluated by flow cytometry using the fluorochromes PNA‐FITC and propidium iodide (PI), respectively. Spermatozoa were incubated with the respective dyes for 5 min at room temperature. For acrosome integrity assessment, viable spermatozoa were identified by co‐staining with PI, allowing exclusion of membrane‐compromised cells. A FACSVerse flow cytometer (BD Biosciences), operated with BD FACSuite software, was used to acquire data from 10,000 events per sample. PNA‐FITC and PI were excited with a 488 nm blue laser; green fluorescence was detected using a 537/32 bandpass (BP) filter and red fluorescence with a 700/54 BP filter. Data were analyzed using WEASEL version 3 (WEHI), applying gating strategies based on forward and side scatter to isolate the sperm population. Sperm populations positive for PI and PNA‐FITC, indicative of membrane and acrosomal damage, respectively, are shown to highlight the extent of abnormalities observed in freshly retrieved, unprocessed spermatozoa.

### 2.7 Histology

Testes were fixed in Bouin solution for at least 4 weeks and embedded in paraffin wax. Specimens were sectioned into 10‐µm‐thick slides, and every fifth slide was stained with hematoxylin‐eosin, imaged, and analyzed as described earlier [28]. The germ epithelium volume was estimated by extracting the area of the lumen from the area of the tubule, according to Cavalieri's principle [29]. The fractions of spermatogenesis (I–VI, VII–VIII, and IX–XII) were distinguished using the point grid approach [30]. Qualitative analysis of seminiferous tubes was performed according to the methods described by the Society of Toxicologic Pathology [31] to assess the presence of degenerated tubules or retained spermatids in the lumen of tubule (spermiation failure). For this end, at least 100 seminiferous tubules were evaluated. More detailed information about the histological evaluation can be found in the Supporting Information.

### 2.8 Electrophoresis and Western Blot

Testes samples were lysed in RIPA buffer. Thereafter, the samples were mixed with Laemmli loading buffer, and electrophoresis and Western blot were applied as previously described [27]. Before incubating membranes with primary and secondary antibodies to target corresponding proteins, membranes were blocked. The proteins of interest were visualized using ECL Select Western blotting Detection Reagent (GE Healthcare Life Sciences, United Kingdom), and membranes were scanned on a ChemiDocTM MP System (Bio‐Rad, France). Images of membranes were processed using ImageLab 6.0.1 software (Bio‐Rad, France), and the “adjusted volume” of the bands was recorded. The α‐tubulin served as a loading control for whole‐tissue testicular lysates; total protein was used as a loading control for sperm lysates using stain‐free gels. For a detailed description of antibodies and procedure, refer to the Supporting Information.

### 2.9 Proteome Profiling of Testes by LC–MS

Testicular lysates in RIPA were digested in solution by trypsin overnight at 37°C. Samples were analyzed on nanoLC system coupled to a trapped ion mobility quadrupole time‐of‐flight mass spectrometer (timsTOF Pro, Bruker, USA). All samples (at least three animals per group) were analyzed in four technical replicates. Only proteins represented in three technical replicates and two biological replicates were used in further processing. Detailed description of LC–MS pipeline is contained in the Supporting Information.

### 2.10 Statistics

The data were processed with GraphPad Prism 8 (GraphPad Software Inc., San Diego, CA, USA). On the basis of Shapiro–Wilk normality distribution tests, paired parametric *t*‐test was and analysis of variance (anova) were used for normally distributed data, whereas Kruskal–Wallis tests were used for data that were not normally distributed. In cases of significant overall findings, differences between individual group pairs were assessed by Tukey's post hoc tests, respectively. Results with *p* less than 0.05 were considered statistically significant. Normally and non‐normally distributed data were expressed as means and medians, respectively.

## 3 Results

### 3.1 Generation of *Sirt1* ^+/−^ Males Led to Reduced Reproductive Outcomes

**FIGURE 1:**
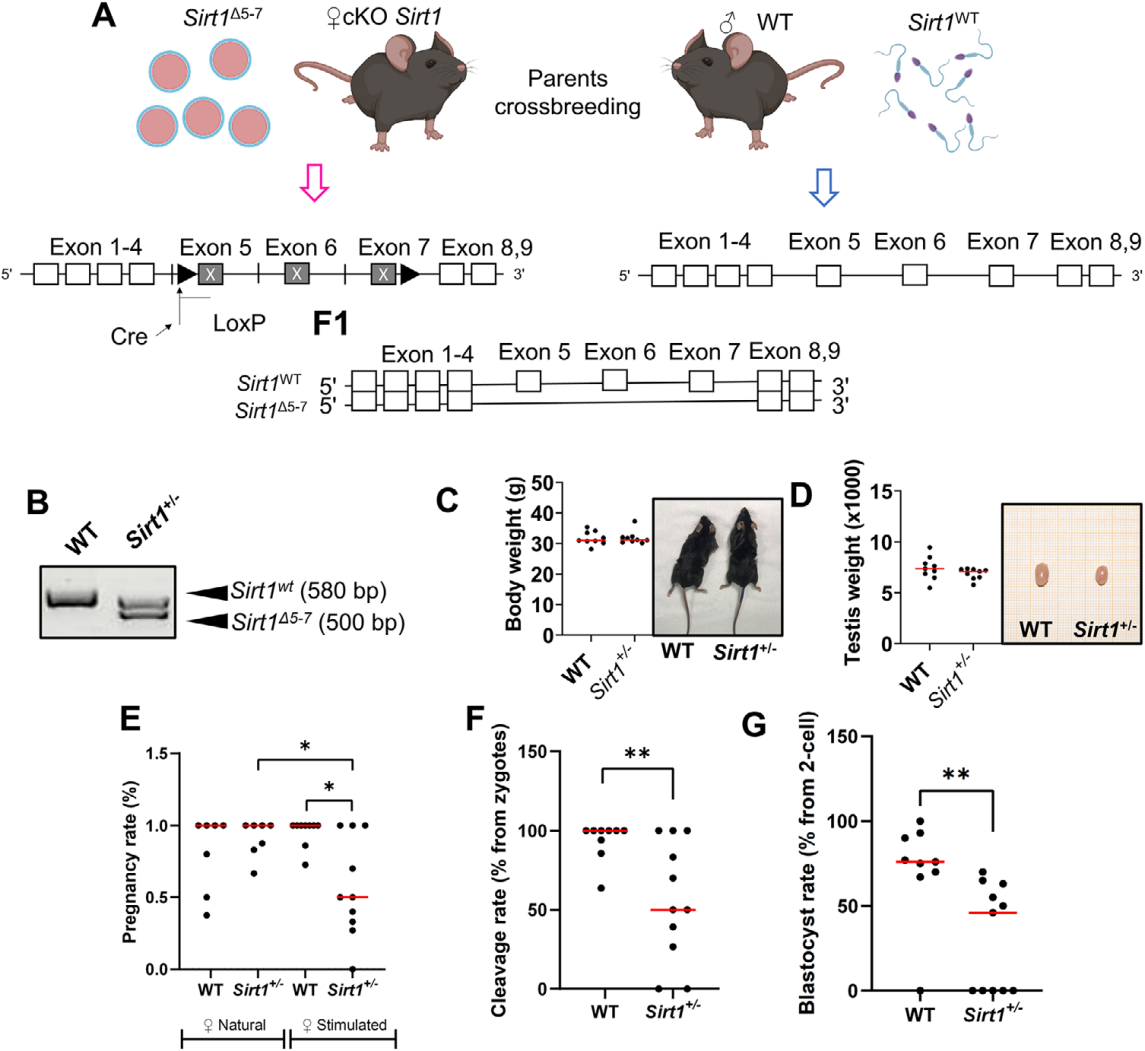
Generation and phenotype of *Sirt1* ^+/−^ male mutant animals. (A) Scheme of breeding and the production of *Sirt1* ^+/−^ animals in F1 offspring generation. Conditional knock‐out (cKO) females, that is, donors of *Sirt1* ^null^ oocytes, were mated with WT males. *Sirt1* ^+/−^ (F1) hemizygotes were genotyped. (B) Sperm genotyping and quantification of *Sirt1* ^+/−^ males via PCR and densitometry of bands belonging to *Sirt1* ^WT^ and *Sirt1* ^Δ5–7^ alleles. (C) Body weight and (D) relative testis weight (weight of both testes/body weight) of wild type (WT) and *Sirt1* ^+/−^ siblings were compared. Representative pictures of both mice models and corresponding testes are shown. (E) Pregnancy outcomes of WT and *Sirt1* ^+^/^−^ males after mating with either naturally cycling or hormonally stimulated WT females to assess the impact of SIRT1 insufficiency on fertilization performance. All females were stimulated using the same protocol to ensure consistency in oocyte quality and origin across groups. (F) Cleavage rate referred to the percentage of zygotes reaching the 2‐cell stage (2 days post‐coitum), whereas (G) blastocyst rate indicated the percentage of 2‐cell embryos that progressed to the blastocyst stage (4 days post‐coitum) as an indirect measure of early embryonic developmental quality. Dots represent individual trials, and lines show median. *p ≤ 0.05; **p ≤ 0.01.

To explore whether the characteristic features of testicular aging are associated with the natural decline in SIRT1 expression that occurs with age, we used a mouse model carrying a heterozygous deletion of the *Sirt1* gene (Figure 1A) that shows reduced SIRT1 protein levels in testicular sections [26]. The presence of the mutation was confirmed by PCR analysis of testicular genomic DNA (Figure 1B), in agreement with our previous findings [26]. Body weight and relative testis weight were comparable between *Sirt1* ^+/−^ males and their WT littermates (Figure 1C,D). To assess whether SIRT1 insufficiency affects the reproductive outcomes *Sirt1* ^+/−^ males, we first analyzed pregnancy outcomes after mating WT and Sirt1^+/−^ males with two types of female partners: naturally cycling females and hormonally stimulated females, which ovulate a higher number of oocytes and therefore pose a greater fertilization challenge [32, 33]. Although *Sirt1* ^+/−^ males achieved pregnancy rates comparable to WT males when mated with females in natural estrus, a marked reduction in the percentage of pregnancies was observed when *Sirt1* ^+/−^ males were mated with hormonally stimulated females, both in comparison to WT males and to *Sirt1* ^+/−^ males paired with non‐stimulated females (Figure 1E). This indicates that the fertility defect associated with SIRT1 insufficiency becomes more evident under high‐demand conditions. As all females were hormonally stimulated following the same protocol, oocytes were obtained under identical conditions and randomly distributed between experimental groups, ensuring comparable origin and expected quality. Therefore, the observed differences in pregnancy outcomes cannot be attributed to maternal factors but rather point to a reduced ability of *Sirt1* ^+/−^ spermatozoa to support embryogenesis after fertilization. These findings suggest that SIRT1 insufficiency compromises not only fertilization capacity as previously we found [26], but also the delivery of essential paternal components required for early embryo development. To assess this more directly, we flushed zygotes from the oviducts of hormonally stimulated females after mating with either WT or *Sirt1* ^+/−^ males and cultured the embryos in vitro. As shown in Figure 1F,G, both the ability of zygotes to progress to the 2‐cell stage and the capacity of 2‐cell embryos to develop into blastocysts were significantly reduced when fertilization was achieved using spermatozoa from *Sirt1* ^+/−^ males. These results indicate that SIRT1‐deficient spermatozoa are not only less efficient in achieving pregnancy under conditions of increased ovulatory pressure but may also have intrinsic limitations in supporting preimplantation embryo development.

### 3.2 SIRT1 Insufficiency Impairs Male Fertility Through Independent Effects on Spermatogenesis and Embryogenesis

**FIGURE 2:**
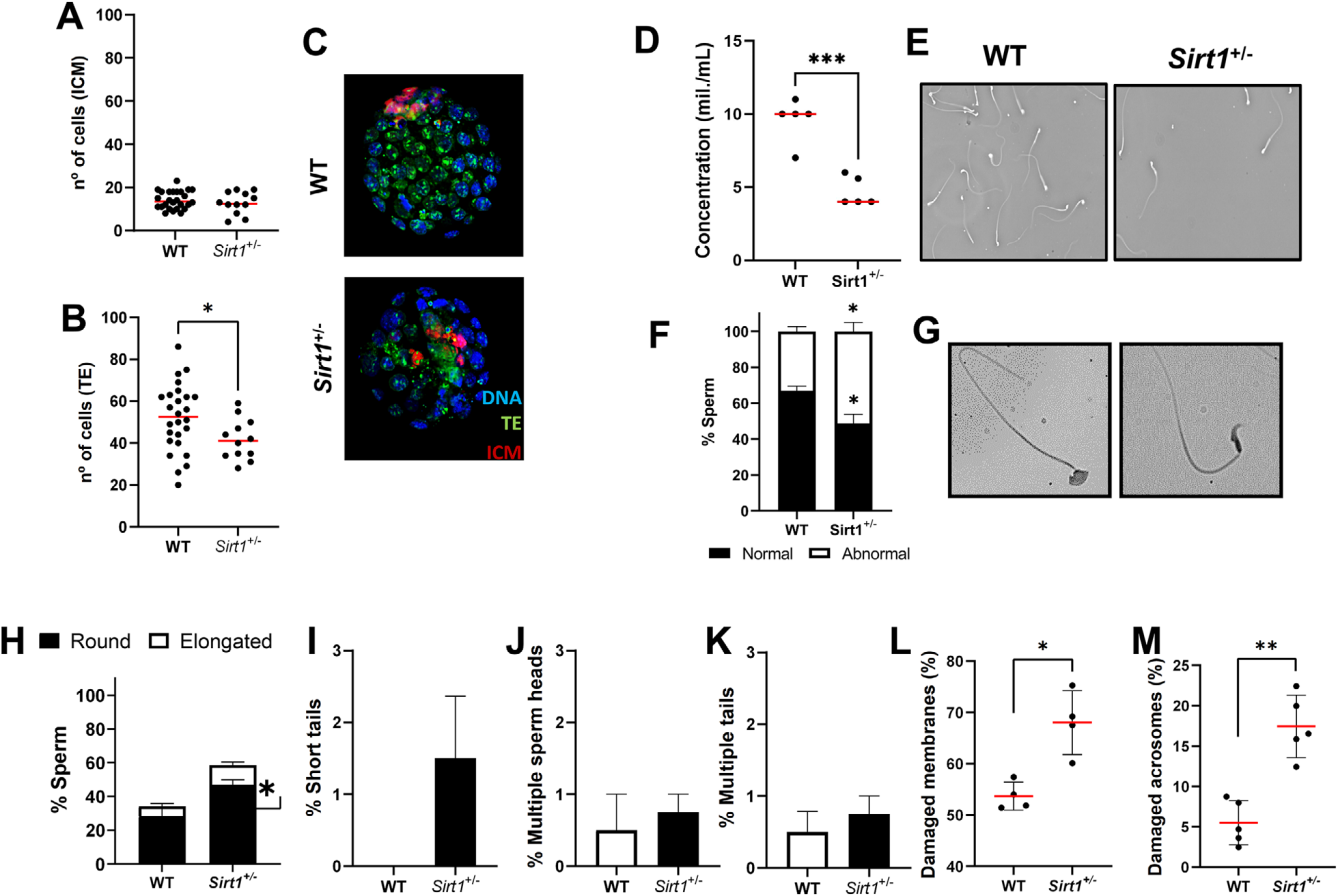
SIRT1 insufficiency impairs male fertility through distinct effects on both spermatogenesis and early embryogenesis. Embryos derived from wild‐type (WT) and *Sirt1* ^+/−^ males were evaluated by differential staining to assess blastocyst quality. The numbers of (A) inner cell mass (ICM) and (B) trophectoderm (TE) cells are shown, along with (C) representative images of stained blastocysts. Spermatogenic output in *Sirt1* ^+^/^−^ males was assessed by measuring (D) sperm concentration and (E) representative microscope fields illustrating sperm density, as well as (F) sperm morphological abnormalities. The most frequent abnormalities observed were round‐ and elongated‐headed spermatozoa, as shown in (G), and their incidence was quantified in WT and *Sirt1* ^+^/^−^ males (H). Less frequent defects, including (I) short tails, (J) multiple heads, and (K) multiple tails, were also documented. In addition, sperm (L) membrane damage and (M) acrosomal integrity after sperm isolation were evaluated only in viable spermatozoa, and results were expressed as percentage. Columns show the mean ± standard error of the mean (sem) of spermatozoa with different abnormalities. Stacked bars represent the percentage distribution of each abnormality per group. Dots represent individual embryos/males, and lines show the median. *p ≤ 0.05; **p ≤ 0.01; ***p ≤ 0.001.

Building on our initial finding that *Sirt1* ^+/−^ males fail to support normal embryo development, we first quantified blastocyst cell allocation by differential staining. Embryos fathered by *Sirt1* ^+/−^ males showed a significant reduction in TE cell number compared to WT controls (*p* < 0.05; Figure 2A–C), whereas the ICM remained unaffected, consistent with delayed development. Next, we analyzed key spermatogenesis indicators in the same cohort of males and performed correlation analyses to assess potential relationships between them and blastocyst cell allocation. Testicular SIRT1 insufficiency led to a marked decrease in sperm count (*p* < 0.05; Figure 2D,E) and a significant increase in morphological defects (*p* < 0.05; Figure 2F,G), particularly round‐headed spermatozoa, which were approximately twice as prevalent in *Sirt1* ^+/−^ samples compared to WT (*p* < 0.05; Figure 2H). Although short tails and multiple heads and tails were also observed, no statistically significant differences were detected among groups (*p* > 0.05; Figure 2J,K). Additionally, other sperm attributes were affected, and both membrane and acrosome integrity were compromised in *Sirt1* ^+/−^ spermatozoa (*p* < 0.05; Figure 2L,M). Despite these defects, correlation analyses revealed no significant association between the sperm parameters evaluated and the altered TE/ICM ratios in blastocysts (Table 1), suggesting that although both spermatogenesis and embryo development are impaired in the absence of full SIRT1 function, these outcomes likely reflect independent manifestations of SIRT1 insufficiency.

**TABLE 1:** Correlations among blastocyst quality and trophectoderm cells and spermatogenesis outputs.

	Spermatozoa								
No. cells	Spermatozoa/mL	Abnormal spermatozoa	Round heads	Elongated heads	Short tails	Multiple heads	Multiple tails	Damaged membranes	Damaged acrosomes
**Inner cell mass**	−0.641 *p = *0.22	0.796 *p = *0.92	0.570 *p = *0.72	0.683 *p = *0.49	−0.574 *p = *0.51	−0.017 *p = *0.98	0.960 *p = *0.73	0.796 *p = *0.78	0.497 *p = *0.87
**Trophectoderm**	0.896 *p = *0.79	−0.698 *p = *0.95	−0.831 *p = *0.86	−0.729 *p = *0.49	0.196 *p = *0.77	0.693 *p = *0.27	−0.964 *p = *0.75	−0.489 *p = *0.76	−0.345 *p = *0.97

### 3.3 Histological Evaluation Reveals Niche‐Level Impairments Underlying Defective Spermiation in *Sirt1* ^+/−^ Testes

**FIGURE 3:**
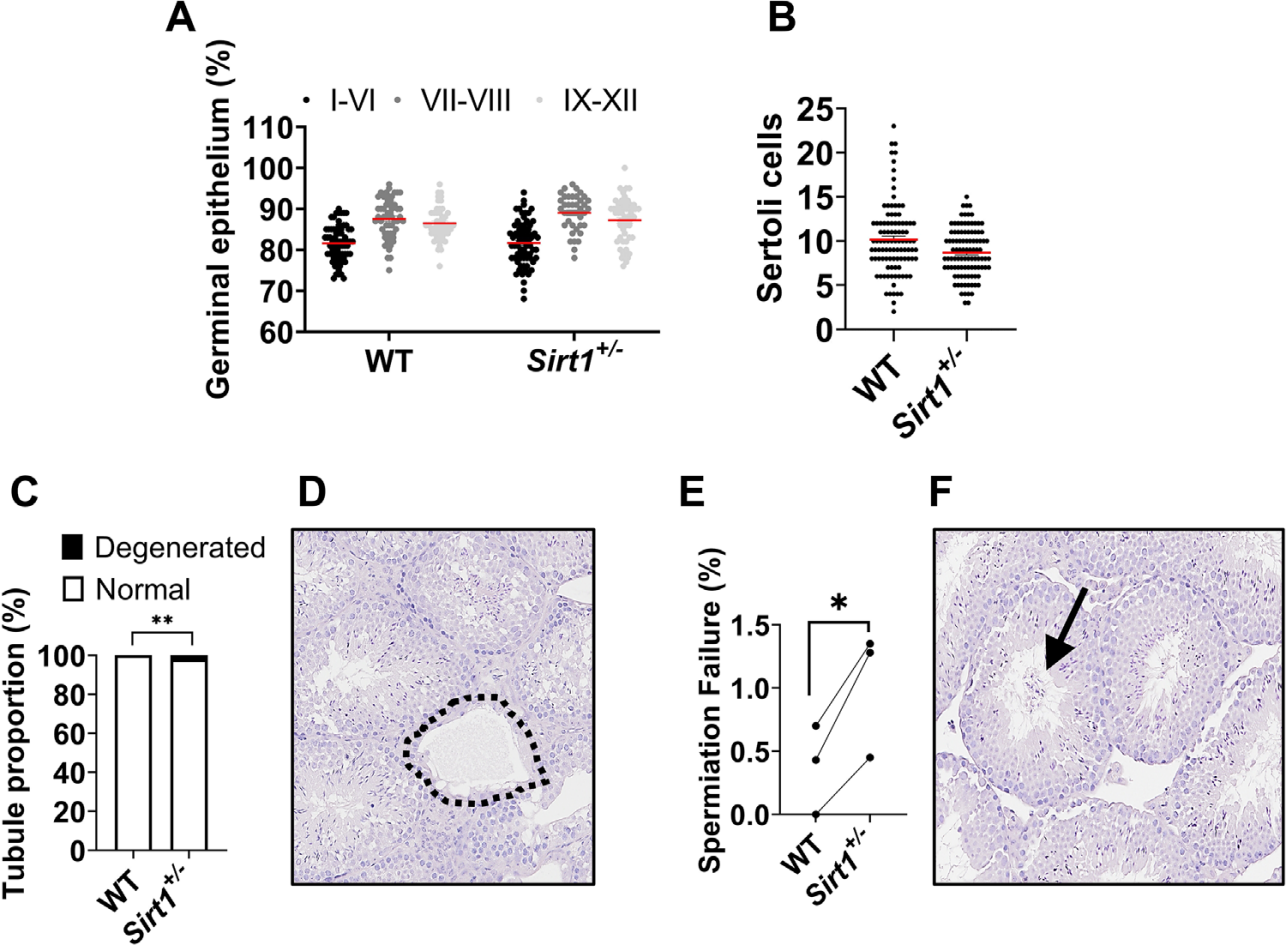
Assessment of somatic apparatus supporting spermatogenesis by counting the (A) proportion of germ epithelia (%) and (B) the number of Sertoli cells in the area of seminiferous tubules in different stages of spermatogenic waves: early (I–VI), middle (VII–VIII), and late (IX–XII) stages. 183 and 187 tubules of three WT and *Sirt1^+/−^* males were evaluated. Assessment of spermatogenesis via (C and D) counting retardation and (D–F) aberrant spermiation (%) while spermatozoa are released in inappropriate stage of spermiation wave (black arrow). Dashed line shows retarded tubule. Stacked columns show cumulative proportion (*p ≤ 0.05; **p ≤ 0.01). 751 and 756 preparations of three WT and *Sirt1^+/−^* males were evaluated. WT, wild type.

Although no evident continuity was observed between spermatogenic efficiency and subsequent embryonic development, our findings revealed a severe impairment in both sperm output and structural integrity. These results prompted us to further investigate testicular function in greater detail. Classical determinants of testicular performance, such as the integrity of seminiferous tubules, the organization of the germinal epithelium, and the abundance of Sertoli cells, are well‐established indicators of male fertility and have been consistently associated with favorable reproductive outcomes [26, 34, 35]. These factors contribute to reproductive competence by regulating spermatogenesis and maintaining the niche of developing germ cells. In light of this, the abnormalities observed in sperm morphology and functional integrity in *Sirt1* ^+/−^ males raised the possibility that such testicular components might be structurally or functionally compromised, potentially affecting the organization or maintenance of the spermatogenic niche function. To investigate this possibility, we conducted detailed histological evaluations of testicular architecture. Although measurements of germinal epithelium depth (Figure 3A) and the number of Sertoli cells per tubule did not reveal significant differences between genotypes (Figure 3B), a markedly higher proportion of degenerated seminiferous tubules was detected in *Sirt1* ^+/−^ testes (Figure 3D,E). Additionally, signs of defective spermiation, characterized by delayed or incomplete release of mature spermatids, were frequently observed in seminiferous tubules from *Sirt1* ^+/−^ testes, in comparison with their WT counterparts (Figure 3F), indicating disrupted coordination of the final stages of spermatogenesis in the absence of full SIRT1 function.

### 3.4 SIRT1 Haploinsufficiency Induces a Testicular Phenotype Resembling Aging

**FIGURE 4:**
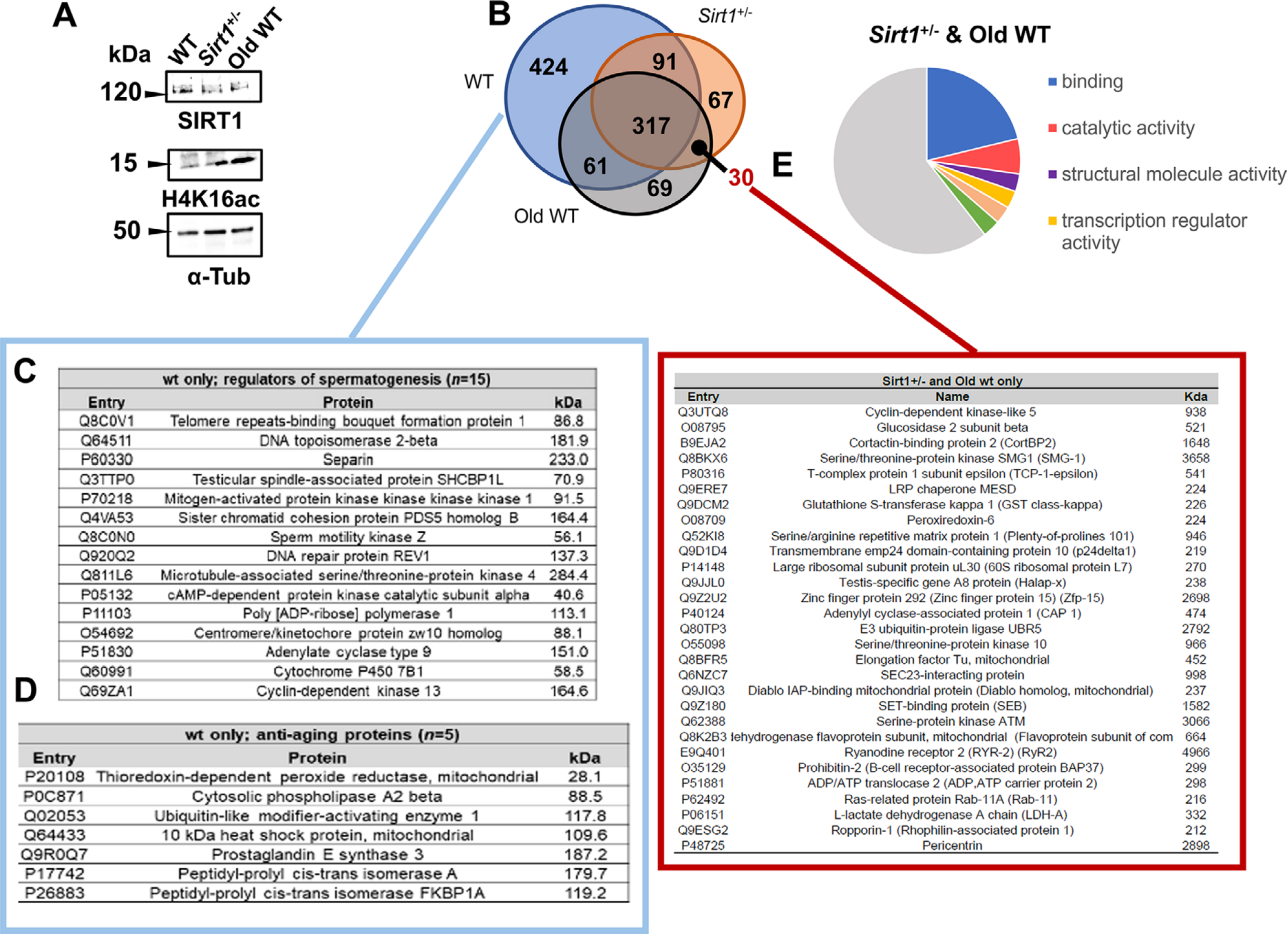
SIRT1 haploinsufficiency induces a testicular phenotype resembling aging. Testes from *Sirt1* ^+^/^−^ males were evaluated and compared to those of aged and wild‐type (WT) controls to assess molecular hallmarks and compared them with testicular aging. (A) Representative cropped images of the Western blot showing proteins presumably affected by aging, namely SIRT1 and its direct target H4K16ac. (B) Supervised principal component analysis (PCA) and clustering of experimental animals (*n* = 3) all in four technical replicates (i.e., *n* = 12). A Ven diagram is shown. Distribution of proteins uniquely expressed in testis of WT males associated to (C) regulators of spermatogenesis but also to sperm function. (D) Distribution of proteins uniquely expressed in testis WT with anti‐aging functions. (E) Distribution of proteins uniquely expressed in testis of *Sirt1* ^+/−^ and old WT males.

On the basis of the testicular alterations observed in *Sirt1* ^+/−^ males, which closely resemble those previously reported in aged animals, including increased tubule degeneration, defective spermiation, reduced sperm count, or increased morphoanomalies [4, 5, 16, 36], we hypothesized that common molecular marks might underlie both conditions. As a first step, we evaluated SIRT1 expression in the testis, along with the H4K16 acetylation, one of its known molecular targets, and compared them with those observed in a naturally aged (old WT) mouse model. Notably, some of assumed changes in both *Sirt1* ^+/−^ and old males were visually observed in both *Sirt1* ^+/−^ and aged males. In particular, in testicular tissue, SIRT1 exhibited reduced signal intensity in agreement with our previous findings showing reduced SIRT1 protein levels in testicular sections [26]. Meanwhile, H4K16ac showed a noticeably stronger signal, suggesting that the testicular environment in *Sirt1* ^+/−^ males may mimic molecular features typically associated with aging. Representative Western blot images are shown (Figure 4A). This prompted us to perform a comparative proteomic analysis to identify specific protein targets that are similarly altered in both *Sirt1* ^+/−^ and old WT mice. On the basis of the whole‐proteome analysis of 1045 proteins (Supporting Information Data S1), we identified 424 proteins only in WT and missing in both *Sirt1* ^+/−^ and aged males (Figure 4B,C; Supporting Information Data S2). Among them are several regulators of chromatin stability and repair, such as separin and DNA repair protein REV1, as well as proteins related to sperm function, including sperm motility kinase Z (SMOK) and the cAMP‐dependent protein kinase catalytic subunit alpha. In accordance with the assumption of aging‐like *Sirt1* ^+/−^ reproductive phenotype, we have found only in WT several remarkable “anti‐aging” proteins (Figure 4D; Supporting Information Data 3). Some of them belong to regulators of spermatogenesis in late stages (Supporting Information Data 4). Moreover, we found 30 proteins expressed exclusively in both *Sirt1* ^+/−^ and old WT (Figure 4E). These proteins particularly belong to protein groups with binding and catalytic activities (Supporting Information Data 5).

## 4 Discussion

Male aging is commonly associated with subfertility, characterized by reduced sperm quality, lower fertilization capacity, and compromised embryo viability. Among the molecular changes accompanying this decline is the reduced expression of SIRT1, an NAD^+^‐dependent deacetylase essential for testicular homeostasis, whose levels decrease in aged spermatids. Yet, the proteomic mechanisms linking SIRT1 insufficiency to testicular dysfunction and their connection to the aging process remain unclear. Here, we demonstrate that partial SIRT1 loss, akin to that occurring in aged males, disrupts testicular integrity and function while inducing a proteomic remodeling that mirrors key molecular features of aging, supporting a central role for SIRT1 in preserving testicular health.

Our findings reveal that testicular SIRT1 insufficiency gives rise to a latent subfertility phenotype. Under baseline conditions, *Sirt1* ^+/−^ males maintain fertility comparable to WT males; however, when subjected to increased reproductive demands, such as mating with hormonally stimulated females, their pregnancy success rates sharply decline. Consistently, embryos conceived with *Sirt1* ^+/−^ spermatozoa, despite developing in non‐stimulated, otherwise equivalent females, show reduced cleavage and blastocyst formation, confirming that the early development defect is intrinsic to the SIRT1‐deficient male gamete, not to maternal factors. This highlights a stress‐revealed subfertility phenotype: under baseline conditions the mutant testes cope, but when reproductive demand surges, or when the embryo relies on paternal inputs for early development, they fail to deliver high‐quality sperm output. These post‐fertilization findings reflect a continuous functional deficiency in those spermatozoa developed under SIRT1 insufficiency, whose impaired capacity becomes evident across sequential stages, from defective morphological development and compromised capacitation‐associated features, such as motility hyperactivation [37], to a reduced ability to support early embryonic development. This continuity underscores that molecular defects acquired prior to fertilization (during development) persist beyond gamete fusion, ultimately affecting early embryo competence.

Proteomic analyses further support this mechanistic model, revealing that only *Sirt1* ^+/−^ and aged WT testes, but not WT, accumulate oxidative stress‐ and metabolism‐related proteins, including PRDX6, GSTK1, LDH‐A, ATM kinase, ADP/ATP translocase ANT2, DIABLO/SMAC, and the E3 ubiquitin ligase UBR5 [38], hallmarks of tissue functioning at its redox and energetic limits under chronic cellular stress [39, 40, 41, 42, 43]. Interestingly, this profile also includes the accumulation of mitochondrial proteins such as SDH‐Fp and EF‐Tu in *Sirt1* ^+/−^ and aging testes, which may reflect compensatory mitochondrial remodeling or dysfunction. This proteomic signature aligns with previous observations showing that spermatozoa derived from *Sirt1* ^+/−^ males exhibit elevated mitochondrial ROS levels [37], potentially linking testicular proteomic alterations to downstream defects in sperm function. Moreover, the absence of key testicular proteins involved in acrosome biogenesis and membrane dynamics, including FKBP1A and PLA2G4B, likely underlies the impaired structural integrity of *Sirt1* ^+/−^ spermatozoa. FKBP1A stabilizes ryanodine‐receptor channels critical for the acrosome reaction [44], whereas PLA2G4B produces lipid mediators essential for sperm membrane fusion [45]. Additionally, the concurrent loss of several proteins involved in mitochondrial protection, protein homeostasis, and lipid signaling likely amplifies cellular dysfunction under SIRT1 insufficiency. The absence of mitochondrial guardians like PRDX3 and HSP10, as previously observed in aged models [46, 47], together with folding chaperones such as PPIA [48] and regulators of proteostasis and lipid metabolism, including UBA1, PLA2G4B, and PTGES3, disrupts key adaptive responses, contributing to oxidative stress, impaired protein folding, and defective membrane signaling [49, 50, 51].

Histological examination of *Sirt1* ^+/−^ testes reinforces the molecular evidence of chronic stress, revealing degeneration of seminiferous tubules and frequent spermiation failures [4, 5]. These structural lesions mirror the proteomic footprint shared by *Sirt1* ^+/−^ and aged WT testes. Concomitant enrichment of proteins involved in cytoskeletal remodeling (CortBP2) [52], ER‐Golgi trafficking (TMED10) [53], and Ca^2+^ handling (RyR2 [54], ANT2 [55], PHB2 [56]) may suggest impaired spermatogenesis [54] and enhanced apoptotic signaling (ANT2 [57]), which could ultimately compromise testicular architecture. Further evidence potentially indicative of meiotic failure and genomic instability may be inferred from the absence of cell‐cycle and DNA‐repair regulators (separin [58], REV1 [59]) together with the presence of pro‐apoptotic mediators (Diablo/Smac and ATM), which could be associated with the observed decrease in sperm concentration. The loss of key signaling effectors such as FKBP1A (Ca^2+^ flux) and PLA2G4B (lipid signaling) could contribute to defects in proteostasis and intercellular communication [49, 50, 51]. All these converging molecular and cellular alterations may suggest that even a partial reduction in SIRT1 could contribute to the development of a testicular phenotype resembling natural aging, supporting the use of the *Sirt1* ^+^/^−^ mouse as a potential model for investigating the early stages of male gonadal aging and for exploring SIRT1‐targeted strategies to preserve reproductive health.

Moreover, our proteomic analyses uncovered the selective loss of key regulators of post‐ejaculatory sperm maturation, namely, SMOK and PKA‐Cα, which govern capacitation events essential for fertilization, in both *Sirt1* ^+/−^ and aged WT testes, and which were exclusively detected in WT controls. SMOK, a sperm‐specific serine/threonine kinase, is essential for initiating and maintaining flagellar beating and progressive motility via phosphorylation cascades that sustain axonemal integrity and energy metabolism [60]. Likewise, the cAMP‐dependent protein kinase catalytic subunit alpha (PKA‐Cα) orchestrates key molecular events during sperm capacitation, including membrane hyperpolarization, activation of motility, and protein tyrosine phosphorylation required for fertilization competence [61, 62, 63, 64]. The absence of both kinases aligns with our previous findings of reduced capacity for hyperactivation in *Sirt1* ^+/−^ spermatozoa compared to WT [26], a phenotype that cannot be explained by conventional sperm parameters alone. Considering that mature spermatozoa are transcriptionally and translationally silent, their functional capacity depends entirely on the molecular composition acquired during spermatogenesis. In this context, the lack of capacitation‐related proteins in *Sirt1* ^+^/^−^ and aged testes supports the idea that testicular SIRT1 insufficiency generates an altered proteomic landscape that may imprint the spermatozoa during their formation. This testicular imprinting, directed by SIRT1, may underlie the reduced fertilization ability observed not only in *Sirt1* ^+^/^−^ males but also in physiologically SIRT1‐insufficient and aged animals, thereby providing a mechanistic framework that links SIRT1‐dependent testicular regulation with both aging and sperm functional competence. Building on this notion of testicular imprinting and given the well‐known epigenetic mode of action of SIRT1, we are currently investigating whether some of the molecular alterations observed in *Sirt1* ^+^/^−^ testes, such as the increase of H4K16ac, a canonical SIRT1 target, are transmitted to the spermatozoa and, ultimately, to the embryo. The hyperacetylation of H4K16 may represent an epigenetic signature potentially linked to the reduced developmental competence observed in embryos derived from *Sirt1* ^+^/^−^ spermatozoa. Interestingly, this phenotype does not correlate with traditional sperm quality parameters, supporting the idea that paternal molecular cues, beyond morphological or motility assessments, should play a pivotal role in embryonic programming. An altered acetylation landscape in sperm chromatin may interfere with proper chromatin compaction and the faithful transmission of epigenetic information required for early development. Ongoing studies in our laboratory aim to elucidate the extent to which these paternal defects are inherited by the embryo, potentially contributing to impaired blastocyst quality in a manner reminiscent of age‐related reproductive decline.

## 5 Conclusion

Collectively, our results suggest that SIRT1 haploinsufficiency may recapitulate several features of an aging‐associated phenotype likely through interconnected alterations affecting testicular structure, sperm performance, and the balance between protective and stress‐related proteins. Notably, these alterations also include the downregulation of key capacitation‐related proteins. Overall, our data indicate that even partial SIRT1 insufficiency may compromise testicular integrity and reproductive competence, being associated with molecular features that closely resemble those observed during reproductive aging.

## Author Contributions

Conceived and designed the experiments: Jan Nevoral and María Iniesta‐Cuerda. Performed the experiments: María Iniesta‐Cuerda and Jan Nevoral. Analyzed the data: María Iniesta‐Cuerda, Jan Nevoral, Jiřina Havránková, and Hedvika Řimnáčová. Contributed reagents/materials/analysis tools: Jan Nevoral, Martin Knytl, Jiří Moravec, František Liška, and Milena Králíčková. Wrote the article: María Iniesta‐Cuerda. Revised the article: Jan Nevoral and Martin Knytl.

## Funding

This work was supported by the Ministry of Education, Youth and Sports of the Czech Republic (MEYS CR): the Cooperatio Programme, research areas MED/DIAG and MED/IMMU of Charles University (J.N.); Project No. SVV 260 773; European Regional Development Fund ‐ Projet “Fighting INfectious Diseases” (No.CZ.02.1.01/0.0/0.0/16_019/00007 87) and the Technology Agency of the Czech Republic (grant No. QL24010123); P JAC project CZ.02.01.01/00/22_010/00029202 MSCA Fellowships CZ‐UK. Additional Funding was provided by the Regional Government of Castilla‐La Mancha (Spain; Project SBPLY‐23‐180225‐000184).

## Ethics Statement

All procedures involving animals were approved by the Animal Welfare Advisory Committee at Charles University, Faculty of Medicine in Pilsen, and by the Animal Welfare Advisory Committee of the Ministry of Education, Youth and Sports of the Czech Republic (Approval No. MSMT‐33798/2021‐4; April 2021). No human data were used.

## Consent

All authors confirm their consent for publication.

## Conflicts of Interest

The authors declare no conflicts of interest.

## Supporting information




**Supporting File 1**: andr70201‐sup‐0001‐SuppMat.docx

**Supporting File 2**: andr70201‐sup‐0002‐DataS1.pdf

**Supporting File 3**: andr70201‐sup‐0003‐DataS2.pdf

**Supporting File 4**: andr70201‐sup‐0004‐DataS3.pdf

**Supporting File 5**: andr70201‐sup‐0005‐DataS4.pdf

**Supporting File 6**: andr70201‐sup‐0006‐DataS5.pdf

## Data Availability

The wide‐proteome results obtained from testicular tissue via LC–MS, supporting the characterization of proteomic profiles in WT, *Sirt1* ^+/−^, and aged WT testes, have been included in Supporting Information. All remaining data generated or analyzed during this study are included in the manuscript.
