# Supplementary material for: Testicular SIRT1 Loss Reveals an Aging‐Like Proteomic Landscape and Precipitates Reproductive Deterioration
Source: Andrology. 2026 Mar 12;14(6):1579–89. doi: 10.1111/andr.70201 (PMC13432521; doi:10.1111/andr.70201)
Supplement: Supplementary file 2 — Supporting File 2: andr70201‐sup‐0002‐DataS1.pdf [file ANDR-14-1579-s003.pdf]

From	Entry	Reviewed	Entry Nam	Protein na	Gene Nam	Organism	Length
RL22_MOU	P67984	reviewed	RL22_MOU	Large ribos	Rpl22	Mus muscu	128
IF2H_MOU	Q9Z0N2	reviewed	IF2H_MOU	Eukaryotic	Eif2s3y	Mus muscu	472
CLH1_MOU	Q68FD5	reviewed	CLH1_MOU	Clathrin he	Cltc	Mus muscu	1675
SAE1_MOU	Q9R1T2	reviewed	SAE1_MOU	SUMO-acti	Sae1 Aos1	Mus muscu	350
ACBG1_MO	Q99PU5	reviewed	ACBG1_MO	Long-chair	Acsbg1 Kia	Mus muscu	721
SUCA_MOU	Q9WUM5	reviewed	SUCA_MOU	Succinate-	Suclg1	Mus muscu	346
IMB1_MOU	P70168	reviewed	IMB1_MOU	Importin su	Kpnb1 Imp	Mus muscu	876
RS3_MOUS	P62908	reviewed	RS3_MOUS	Small ribos	Rps3	Mus muscu	243
TCPH_MOU	P80313	reviewed	TCPH_MOU	T-complex	Cct7 Ccth	Mus muscu	544
CE290_MO	Q6A078	reviewed	CE290_MO	Centrosom	Cep290 Kia	Mus muscu	2472
PIWL1_MO	Q9JMB7	reviewed	PIWL1_MO	Piwi-like pr	Piwil1 Miw	Mus muscu	862
GD1L1_MO	Q8VE33	reviewed	GD1L1_MO	Gangliosid	Gdap1l1 G	Mus muscu	370
IPP2_MOU	Q9DCL8	reviewed	IPP2_MOU	Protein pho	Ppp1r2	Mus muscu	206
KI20B_MOU	Q80WE4	reviewed	KI20B_MOU	Kinesin-like	Kif20b Mph	Mus muscu	1774
ERH_MOUS	P84089	reviewed	ERH_MOUS	Enhancer c	Erh	Mus muscu	104
TET1_MOU	Q3URK3	reviewed	TET1_MOU	Methylcyto	Tet1 Cxxc6	Mus muscu	2039
FXR1_MOU	Q61584	reviewed	FXR1_MOU	RNA-bindir	Fxr1 Fxr1h	Mus muscu	677
RS12_MOU	P63323	reviewed	RS12_MOU	Small ribos	Rps12	Mus muscu	132
EF1A1_MO	P10126	reviewed	EF1A1_MO	Elongation	Eef1a1 Eef	Mus muscu	462
KI67_MOU	E9PVX6	reviewed	KI67_MOU	Proliferatic	Mki67	Mus muscu	3177
DYL1_MOU	P63168	reviewed	DYL1_MOU	Dynein ligh	Dynll1 Dlc1	Mus muscu	89
LDHB_MOU	P16125	reviewed	LDHB_MOU	L-lactate d	Ldhb Ldh-2	Mus muscu	334
ALDOA_MO	P05064	reviewed	ALDOA_MO	Fructose-b	Aldoa Aldo	Mus muscu	364
RS23_MOU	P62267	reviewed	RS23_MOU	Small ribos	Rps23	Mus muscu	143
CHD7_MO	A2AJK6	reviewed	CHD7_MO	Chromodo	Chd7	Mus muscu	2986
TCPD_MOU	P80315	reviewed	TCPD_MOU	T-complex	Cct4 Cctd	Mus muscu	539
EF2_MOUS	P58252	reviewed	EF2_MOUS	Elongation	Eef2	Mus muscu	858
DDX3L_MO	P16381	reviewed	DDX3L_MO	Putative AT	D1Pas1 Pl1	Mus muscu	660
BASI_MOU	P18572	reviewed	BASI_MOU	Basigin (Ba	Bsg	Mus muscu	389
PRDX1_MO	P35700	reviewed	PRDX1_MO	Peroxiredo	Prdx1 Msp2	Mus muscu	199
PRDX4_MO	O08807	reviewed	PRDX4_MO	Peroxiredo	Prdx4	Mus muscu	274
MRP_MOU	P28667	reviewed	MRP_MOU	MARCKS-re	Marcksl1 M	Mus muscu	200
REM1_MOU	O35929	reviewed	REM1_MOU	GTP-bindir	Rem1 Rem	Mus muscu	297
NASP_MOU	Q99MD9	reviewed	NASP_MOU	Nuclear au	Nasp	Mus muscu	773
KMT2C_MO	Q8BRH4	reviewed	KMT2C_MO	Histone-lys	Kmt2c Mll3	Mus muscu	4903
RS18_MOU	P62270	reviewed	RS18_MOU	Small ribos	Rps18	Mus muscu	152
HYOU1_MO	Q9JKR6	reviewed	HYOU1_MO	Hypoxia up	Hyou1 Grp	Mus muscu	999
ACE_MOUS	P09470	reviewed	ACE_MOUS	Angiotensi	Ace Dcp1	Mus muscu	1312
ULA1_MOU	Q8VBW6	reviewed	ULA1_MOU	NEDD8-act	Nae1 Appb	Mus muscu	534
SACA9_MO	Q7TPM5	reviewed	SACA9_MO	Sperm acro	Spaca9	Mus muscu	168
CENPC_MO	P49452	reviewed	CENPC_MO	Centromer	Cenpc Cen	Mus muscu	906
ROA2_MOU	O88569	reviewed	ROA2_MOU	Heterogen	Hnrnpa2b1	Mus muscu	353
VIME_MOU	P20152	reviewed	VIME_MOU	Vimentin	Vim	Mus muscu	466
PGP_MOUS	Q8CHP8	reviewed	PGP_MOUS	Glycerol-3-	Pgp	Mus muscu	321
TKFC_MOU	Q8VC30	reviewed	TKFC_MOU	Triokinase/	Tkfc Dak	Mus muscu	578
MACF1_MO	Q9QXZ0	reviewed	MACF1_MO	Microtubul	Macf1 Acf7	Mus muscu	7354
GLNA_MOU	P15105	reviewed	GLNA_MOU	Glutamine	Glul Glns	Mus muscu	373

VDAC2_MO	Q60930	reviewed	VDAC2_MO	Voltage-de	Vdac2 Vda	Mus muscu	295
PXL2A_MO	Q9CYH2	reviewed	PXL2A_MO	Peroxiredo	Prxl2a Fam	Mus muscu	218
SUMO2_M	P61957	reviewed	SUMO2_M	Small ubiq	Sumo2 Sm	Mus muscu	95
NRAP_MOI	Q80XB4	reviewed	NRAP_MOI	Nebulin-re	Nrap	Mus muscu	1728
PSB5_MOU	O55234	reviewed	PSB5_MOU	Proteasom	Psmb5	Mus muscu	264
RS3A_MOU	P97351	reviewed	RS3A_MOU	Small ribos	Rps3a Rps	Mus muscu	264
2AAA_MOU	Q76MZ3	reviewed	2AAA_MOU	Serine/thre	Ppp2r1a	Mus muscu	589
GTR3_MOU	P32037	reviewed	GTR3_MOU	Solute carr	Slc2a3 Glu	Mus muscu	493
PSB1_MOU	O09061	reviewed	PSB1_MOU	Proteasom	Psmb1	Mus muscu	240
RL3_MOUS	P27659	reviewed	RL3_MOUS	Large ribos	Rpl3	Mus muscu	403
RL4_MOUS	Q9D8E6	reviewed	RL4_MOUS	Large ribos	Rpl4	Mus muscu	419
FCL_MOUS	P23591	reviewed	FCL_MOUS	GDP-L-fuc	Gfus P35b	Mus muscu	321
HMCS2_M	P54869	reviewed	HMCS2_M	Hydroxyme	Hmgcs2	Mus muscu	508
TR150_MO	Q569Z6	reviewed	TR150_MO	Thyroid hor	Thrap3 Tra	Mus muscu	951
MATR3_MO	Q8K310	reviewed	MATR3_MO	Matrin-3	Matr3	Mus muscu	846
IF4A1_MOI	P60843	reviewed	IF4A1_MOI	Eukaryotic	Eif4a1 Ddx	Mus muscu	406
TCPZ_MOU	P80317	reviewed	TCPZ_MOU	T-complex	Cct6a Cct6	Mus muscu	531
TBB4B_MC	P68372	reviewed	TBB4B_MC	Tubulin bet	Tubb4b Tul	Mus muscu	445
HMGB1_M	P63158	reviewed	HMGB1_M	High mobil	Hmgb1 Hm	Mus muscu	215
PARK7_MC	Q99LX0	reviewed	PARK7_MC	Parkinson	Park7	Mus muscu	189
CLGN_MOI	P52194	reviewed	CLGN_MOI	Calmegin (	Clgn Meg1	Mus muscu	611
DHSO_MO	Q64442	reviewed	DHSO_MO	Sorbitol de	Sord Sdh1	Mus muscu	357
ANKAR_MO	A2RT91	reviewed	ANKAR_MO	Ankyrin and	Ankar	Mus muscu	1465
HYEP_MOU	Q9D379	reviewed	HYEP_MOU	Epoxide hy	Ephx1	Mus muscu	455
ODPB_MOI	Q9D051	reviewed	ODPB_MO	Pyruvate de	Pdhb	Mus muscu	359
TRDN_MOI	E9Q9K5	reviewed	TRDN_MOI	Triadin	Trdn	Mus muscu	693
COX6C_MO	Q9CPQ1	reviewed	COX6C_MO	Cytochrom	Cox6c	Mus muscu	76
CYB5_MOU	P56395	reviewed	CYB5_MOU	Cytochrom	Cyb5a Cyb	Mus muscu	134
IF2A_MOUS	Q6ZWX6	reviewed	IF2A_MOUS	Eukaryotic	Eif2s1 Eif2	Mus muscu	315
ATPD_MOU	Q9D3D9	reviewed	ATPD_MOU	ATP syntha	Atp5f1d At	Mus muscu	168
PCBP2_MO	Q61990	reviewed	PCBP2_MO	Poly(rC)-bi	Pcbp2 Cbp	Mus muscu	362
RP1L1_MO	Q8CGM2	reviewed	RP1L1_MO	Retinitis pi	Rp1l1 Rp1l	Mus muscu	1859
RL18A_MO	P62717	reviewed	RL18A_MO	Large ribos	Rpl18a	Mus muscu	176
ALRF2_MO	Q9JJW6	reviewed	ALRF2_MO	Aly/REF ex	Alyref2 Ref	Mus muscu	218
Q3ZBD7	Q3ZBD7	reviewed	G6PI_BOVI	Glucose-6-	GPI	Bos taurus	557
PPM1B_MO	P36993	reviewed	PPM1B_MO	Protein pho	Ppm1b Pp2	Mus muscu	390
FAS_MOUS	P19096	reviewed	FAS_MOUS	Fatty acid s	Fasn	Mus muscu	2504
NPM_MOU	Q61937	reviewed	NPM_MOU	Nucleopho	Npm1	Mus muscu	292
ACINU_MC	Q9JIX8	reviewed	ACINU_MC	Apoptotic c	Acin1 Acin	Mus muscu	1338
ASPM_MOI	Q8CJ27	reviewed	ASPM_MOI	Abnormal s	Aspm Caln	Mus muscu	3122
PDIA4_MO	P08003	reviewed	PDIA4_MO	Protein dis	Pdia4 Cai E	Mus muscu	638
RRBP1_MC	Q99PL5	reviewed	RRBP1_MC	Ribosome-	Rrbp1	Mus muscu	1605
HBA_MOUS	P01942	reviewed	HBA_MOUS	Hemoglobi	Hba Hba-a	Mus muscu	142
DYH2_MOI	P0C6F1	reviewed	DYH2_MOI	Dynein axo	Dnah2 Dna	Mus muscu	4456
FKBP4_MC	P30416	reviewed	FKBP4_MC	Peptidyl-pr	Fkbp4 Fkpl	Mus muscu	458
PSMD2_MO	Q8VDM4	reviewed	PSMD2_MO	26S protea	Psmd2	Mus muscu	908
ELOB_MOU	P62869	reviewed	ELOB_MOU	Elongin-B (	Elob Tceb2	Mus muscu	118
LDHC_MOI	P00342	reviewed	LDHC_MOI	L-lactate d	Ldhc Ldh-3	Mus muscu	332

AKA12_MC	Q9WTQ5	reviewed	AKA12_MC	A-kinase a	Akap12 Ga	Mus muscu	1684
EF1G_MOU	Q9D8N0	reviewed	EF1G_MOU	Elongation	Eef1g	Mus muscu	437
DYST_MOU	Q91ZU6	reviewed	DYST_MOU	Dystonin (I	Dst Bpag1	Mus muscu	7393
ODPAT_MC	P35487	reviewed	ODPAT_MC	Pyruvate d	Pdha2 Pdh	Mus muscu	391
NDUAC_M	Q7TMF3	reviewed	NDUAC_M	NADH deh	Ndufa12	Mus muscu	145
STT3A_MO	P46978	reviewed	STT3A_MO	Dolichyl-di	Stt3a Itm1	Mus muscu	705
PRC2C_MC	Q3TLH4	reviewed	PRC2C_MC	Protein PRI	Prrc2c Bat	Mus muscu	2846
RLA0_MOU	P14869	reviewed	RLA0_MOU	Large ribos	Rplp0 Arbp	Mus muscu	317
QCR2_MOU	Q9DB77	reviewed	QCR2_MOU	Cytochrom	Uqcrc2	Mus muscu	453
IPO5_MOU	Q8BKC5	reviewed	IPO5_MOU	Importin-5	Ipo5 Kpnb3	Mus muscu	1097
ARI5B_MO	Q8BM75	reviewed	ARI5B_MO	AT-rich inte	Arid5b Des	Mus muscu	1188
ARK72_MC	Q8CG76	reviewed	ARK72_MC	Aflatoxin B	Akr7a2 Afa	Mus muscu	367
RAN_MOUS	P62827	reviewed	RAN_MOUS	GTP-bindir	Ran Rasl2-	Mus muscu	216
HSP72_MC	P17156	reviewed	HSP72_MC	Heat shock	Hspa2 Hcp	Mus muscu	633
BD1L1_MC	E9Q6J5	reviewed	BD1L1_MC	Biorientati	Bod1l Kiaa	Mus muscu	3032
G3P_MOUS	P16858	reviewed	G3P_MOUS	Glyceralde	Gapdh Gap	Mus muscu	333
TBB5_MOU	P99024	reviewed	TBB5_MOU	Tubulin bet	Tubb5	Mus muscu	444
RL13_MOU	P47963	reviewed	RL13_MOU	Large ribos	Rpl13	Mus muscu	211
HMOX2_M	O70252	reviewed	HMOX2_M	Heme oxyg	Hmox2	Mus muscu	315
1433B_MC	Q9CQV8	reviewed	1433B_MC	14-3-3 prot	Ywhab	Mus muscu	246
MA7D3_MC	A2AEY4	reviewed	MA7D3_MC	MAP7 dom	Map7d3 M	Mus muscu	876
SRRM2_MC	Q8BTI8	reviewed	SRRM2_MC	Serine/argi	Srrm2 Kiaa	Mus muscu	2703
ADRM1_MC	Q9JKV1	reviewed	ADRM1_MC	Proteasom	Adrm1 Gp1	Mus muscu	407
MDHM_MC	P08249	reviewed	MDHM_MC	Malate deh	Mdh2 Mor1	Mus muscu	338
CAND1_MC	Q6ZQ38	reviewed	CAND1_MC	Cullin-asso	Cand1 D10	Mus muscu	1230
MY18A_MC	Q9JMH9	reviewed	MY18A_MC	Unconvent	Myo18a My	Mus muscu	2050
ASGL1_MC	Q8C0M9	reviewed	ASGL1_MC	Isoaspartyl	Asrgl1	Mus muscu	326
RP1_MOUS	P56716	reviewed	RP1_MOUS	Oxygen-reg	Rp1 Orp1 F	Mus muscu	2095
C8AP2_MC	Q9WUF3	reviewed	C8AP2_MC	CASP8-ass	Casp8ap2	Mus muscu	1962
STMN1_MC	P54227	reviewed	STMN1_MC	Stathmin (I	Stmn1 Lag	Mus muscu	149
F16P1_MO	Q9QXD6	reviewed	F16P1_MO	Fructose-1	Fbp1 Fbp F	Mus muscu	338
TKT_MOUS	P40142	reviewed	TKT_MOUS	Transketol	Tkt	Mus muscu	623
LAT1_MOU	Q9Z127	reviewed	LAT1_MOU	Large neut	Slc7a5 Lat	Mus muscu	512
ALDH2_MC	P47738	reviewed	ALDH2_MC	Aldehyde d	Aldh2 Ahd-	Mus muscu	519
DYLT1_MO	P51807	reviewed	DYLT1_MO	Dynein ligh	Dynlt1 Tcte	Mus muscu	113
MAOX_MO	P06801	reviewed	MAOX_MO	NADP-dep	Me1 Mod-1	Mus muscu	572
DDX5_MOU	Q61656	reviewed	DDX5_MOU	Probable A	Ddx5 Tnz2	Mus muscu	614
TXND2_MC	Q6P902	reviewed	TXND2_MC	Thioredoxi	Txndc2 Spt	Mus muscu	515
RL23A_MO	P62751	reviewed	RL23A_MO	Large ribos	Rpl23a	Mus muscu	156
PRS7_MOU	P46471	reviewed	PRS7_MOU	26S protea	Psmc2 Mss	Mus muscu	433
TERA_MOU	Q01853	reviewed	TERA_MOU	Transitiona	Vcp	Mus muscu	806
CISY_MOU	Q9CZU6	reviewed	CISY_MOU	Citrate syn	Cs	Mus muscu	464
TCF20_MO	Q9EPQ8	reviewed	TCF20_MO	Transcripti	Tcf20 Spbp	Mus muscu	1987
TYB10_MO	Q6ZWY8	reviewed	TYB10_MO	Thymosin b	Tmsb10 Pt	Mus muscu	44
HS74L_MC	P48722	reviewed	HS74L_MC	Heat shock	Hspa4l Apg	Mus muscu	838
ANR11_MC	E9Q4F7	reviewed	ANR11_MC	Ankyrin rep	Ankrd11	Mus muscu	2643
DDX4_MOU	Q61496	reviewed	DDX4_MOU	ATP-depen	Ddx4 Mvh V	Mus muscu	702
AK1A1_MC	Q9JII6	reviewed	AK1A1_MC	Aldo-keto r	Akr1a1 Akr	Mus muscu	325

RSPH1_MC	Q8VIG3	reviewed	RSPH1_MC	Radial spo	Rsph1 Tsga	Mus muscu	301
ACON_MO	Q99KI0	reviewed	ACON_MO	Aconitate h	Aco2	Mus muscu	780
MAP4_MOI	P27546	reviewed	MAP4_MOI	Microtubul	Map4 Mtap	Mus muscu	1125
DX39A_MC	Q8VDW0	reviewed	DX39A_MC	ATP-depen	Ddx39a Dd	Mus muscu	427
TLN1_MOL	P26039	reviewed	TLN1_MOL	Talin-1	Tln1 Tln	Mus muscu	2541
DESP_MOL	E9Q557	reviewed	DESP_MOL	Desmoplal	Dsp	Mus muscu	2883
RL5_MOUS	P47962	reviewed	RL5_MOUS	Large ribos	Rpl5	Mus muscu	297
HDAC6_M(	Q9Z2V5	reviewed	HDAC6_M(	Histone de	Hdac6	Mus muscu	1149
HNRPK_M(	P61979	reviewed	HNRPK_M(	Heterogen	Hnrnpk Hn	Mus muscu	463
XIRP2_MOI	Q4U4S6	reviewed	XIRP2_MOI	Xin actin-b	Xirp2 Cmya	Mus muscu	3784
GSTM2_M(	P15626	reviewed	GSTM2_M(	Glutathion	Gstm2	Mus muscu	218
1433T_MO	P68254	reviewed	1433T_MO	14-3-3 pro	Ywhaq	Mus muscu	245
HS90A_MC	P07901	reviewed	HS90A_MC	Heat shock	Hsp90aa1	Mus muscu	733
TRNK1_MC	Q8BV79	reviewed	TRNK1_MC	TPR and ar	Trank1 Gm	Mus muscu	2999
HS90B_MC	P11499	reviewed	HS90B_MC	Heat shock	Hsp90ab1	Mus muscu	724
NACAM_M	P70670	reviewed	NACAM_M	Nascent po	Naca Gm1	Mus muscu	2187
DYHC2_M(	Q45VK7	reviewed	DYHC2_M(	Cytoplasm	Dync2h1 D	Mus muscu	4306
CH60_MOI	P63038	reviewed	CH60_MOI	60 kDa hea	Hspd1 Hsp	Mus muscu	573
PGK2_MOL	P09041	reviewed	PGK2_MOL	Phosphogl	Pgk2 Pgk-2	Mus muscu	417
4F2_MOUS	P10852	reviewed	4F2_MOUS	Amino acic	Slc3a2 Md	Mus muscu	526
IVD_MOUS	Q9JHI5	reviewed	IVD_MOUS	Isovaleryl-(	Ivd	Mus muscu	424
ATPB_MOL	P56480	reviewed	ATPB_MOL	ATP syntha	Atp5f1b At	Mus muscu	529
TX101_MO	Q9JMI7	reviewed	TX101_MO	Testis-expr	Tex101	Mus muscu	250
CCDC7_M	Q9D541	reviewed	CCDC7_M	Coiled-coil	Ccdc7 Biot	Mus muscu	371
TITIN_MOL	A2ASS6	reviewed	TITIN_MOL	Titin (EC 2.	Ttn	Mus muscu	35213
AP2B1_MC	Q9DBG3	reviewed	AP2B1_MC	AP-2 comp	Ap2b1 Clap	Mus muscu	937
AMRP_MOI	P55302	reviewed	AMRP_MOI	Alpha-2-ma	Lrpap1	Mus muscu	360
TOM34_M(	Q9CYG7	reviewed	TOM34_M(	Mitochond	Tomm34	Mus muscu	309
MYH10_M(	Q61879	reviewed	MYH10_M(	Myosin-10	Myh10	Mus muscu	1976
TPIS_MOU	P17751	reviewed	TPIS_MOU	Triosephos	Tpi1 Tpi	Mus muscu	249
1433Z_MO	P63101	reviewed	1433Z_MO	14-3-3 pro	Ywhaz	Mus muscu	245
CABS1_MC	Q8C633	reviewed	CABS1_MC	Calcium-b	Cabs1	Mus muscu	391
SYCP2_MC	Q9CUU3	reviewed	SYCP2_MC	Synaptone	Sycp2 Scp2	Mus muscu	1500
ODFP2_M(	A3KGV1	reviewed	ODFP2_M(	Outer dens	Odf2 Odf84	Mus muscu	830
MYH11_M(	O08638	reviewed	MYH11_M(	Myosin-11	Myh11	Mus muscu	1972
RL6_MOUS	P47911	reviewed	RL6_MOUS	Large ribos	Rpl6	Mus muscu	296
MORC4_M	Q8BMD7	reviewed	MORC4_M	MORC fam	Morc4 Zcw	Mus muscu	928
CALR_MOL	P14211	reviewed	CALR_MOL	Calreticuli	Calr	Mus muscu	416
GRP75_MC	P38647	reviewed	GRP75_MC	Stress-70 p	Hspa9 Grp	Mus muscu	679
TBB3_MOL	Q9ERD7	reviewed	TBB3_MOL	Tubulin be	Tubb3	Mus muscu	450
TPP2_MOL	Q64514	reviewed	TPP2_MOL	Tripeptidyl	Tpp2	Mus muscu	1262
LANC1_M(	O89112	reviewed	LANC1_M(	Glutathion	Lancl1 Gpr	Mus muscu	399
VP13C_MC	Q8BX70	reviewed	VP13C_MC	Intermemb	Vps13c Kia	Mus muscu	3748
EIF3I_MOU	Q9QZD9	reviewed	EIF3I_MOU	Eukaryotic	Eif3i Eif3s2	Mus muscu	325
SCO2A_M(	Q9JJN4	reviewed	SCO2A_M(	Succinyl-C	Oxct2a	Mus muscu	520
SKT_MOUS	A2AQ25	reviewed	SKT_MOUS	Sickle tail p	Skt Etl4 Kia	Mus muscu	1946
MYL6B_MC	Q8CI43	reviewed	MYL6B_MC	Myosin ligh	Myl6b	Mus muscu	207
GSTM1_M(	P10649	reviewed	GSTM1_M(	Glutathion	Gstm1	Mus muscu	218

LMNB1_M(	P14733	reviewed	LMNB1_M(	Lamin-B1	Lmnb1	Mus muscı	588
BASP1_MC	Q91XV3	reviewed	BASP1_MC	Brain acid :	Basp1 Nap	Mus muscı	226
KPYM_MOI	P52480	reviewed	KPYM_MOI	Pyruvate ki	Pkm Pk3 Pl	Mus muscı	531
RL14_MOL	Q9CR57	reviewed	RL14_MOL	Large ribos	Rpl14	Mus muscı	217
TCPG_MOI	P80318	reviewed	TCPG_MOI	T-complex	Cct3 Cctg	Mus muscı	545
SFPQ_MOI	Q8VIJ6	reviewed	SFPQ_MOI	Splicing fa	Sfpq Psf	Mus muscı	699
DYH8_MOI	Q91XQ0	reviewed	DYH8_MOI	Dynein axo	Dnah8 Dna	Mus muscı	4731
PUR6_MOI	Q9DCL9	reviewed	PUR6_MOI	Bifunctiona	Paics	Mus muscı	425
GUAA_MOI	Q3THK7	reviewed	GUAA_MOI	GMP synth	Gmps	Mus muscı	693
LRC45_MC	Q8CIM1	reviewed	LRC45_MC	Leucine-ric	Lrrc45	Mus muscı	670
CX7A2_MC	P48771	reviewed	CX7A2_MC	Cytochrom	Cox7a2 Cc	Mus muscı	83
EVPL_MOL	Q9D952	reviewed	EVPL_MOL	Envoplakin	Evpl	Mus muscı	2035
SRSF1_MC	Q6PDM2	reviewed	SRSF1_MC	Serine/argi	Srsf1 Sfrs1	Mus muscı	248
PDCD5_M(	P56812	reviewed	PDCD5_M(	Programm	Pdcd5 Tfar	Mus muscı	126
SYNE2_MC	Q6ZWQ0	reviewed	SYNE2_MC	Nesprin-2 (	Syne2	Mus muscı	6874
ENOA_MOI	P17182	reviewed	ENOA_MOI	Alpha-enol	Eno1 Eno-:	Mus muscı	434
MTCH2_M(	Q791V5	reviewed	MTCH2_M(	Mitochond	Mtch2 Mim	Mus muscı	303
ALDR_MOI	P45376	reviewed	ALDR_MOI	Aldo-keto r	Akr1b1 Akr	Mus muscı	316
ZN638_MC	Q61464	reviewed	ZN638_MC	Zinc finger	Znf638 Np:	Mus muscı	1960
CY1_MOUS	Q9D0M3	reviewed	CY1_MOUS	Cytochrom	Cyc1	Mus muscı	325
GPX4_MOI	O70325	reviewed	GPX4_MOI	Phospholip	Gpx4	Mus muscı	197
ECHM_MO	Q8BH95	reviewed	ECHM_MO	Enoyl-CoA	Echs1	Mus muscı	290
HMGB2_M	P30681	reviewed	HMGB2_M	High mobil	Hmgb2 Hm	Mus muscı	210
KTN1_MOL	Q61595	reviewed	KTN1_MOL	Kinectin	Ktn1	Mus muscı	1327
ATPA_MOL	Q03265	reviewed	ATPA_MOL	ATP syntha	Atp5f1a At	Mus muscı	553
MINT_MOL	Q62504	reviewed	MINT_MOL	Msx2-inter	Spen Kiaa(	Mus muscı	3644
AKAP9_MC	Q70FJ1	reviewed	AKAP9_MC	A-kinase a	Akap9 Kiaa	Mus muscı	3797
IMPA1_MO	O55023	reviewed	IMPA1_MO	Inositol mc	Impa1	Mus muscı	277
RUVB2_MC	Q9WTM5	reviewed	RUVB2_MC	RuvB-like 2	Ruvbl2	Mus muscı	463
SF3B3_MC	Q921M3	reviewed	SF3B3_MC	Splicing fa	Sf3b3 Kiaa	Mus muscı	1217
DDAH1_M(	Q9CWS0	reviewed	DDAH1_M(	N(G),N(G)-	Ddah1	Mus muscı	285
CP17A_MC	P27786	reviewed	CP17A_MC	Steroid 17-	Cyp17a1 C	Mus muscı	507
EF1D_MOI	P57776	reviewed	EF1D_MOI	Elongation	Eef1d	Mus muscı	281
HBB1_MOI	P02088	reviewed	HBB1_MOI	Hemoglobi	Hbb-b1	Mus muscı	147
H4_MOUSI	P62806	reviewed	H4_MOUSI	Histone H4	H4c1 Hist1	Mus muscı	103
TRXR3_MO	Q99MD6	reviewed	TRXR3_MO	Thioredoxi	Txnrd3 Tgr	Mus muscı	652
AKAP4_MC	Q60662	reviewed	AKAP4_MC	A-kinase a	Akap4 Aka	Mus muscı	849
YBOX3_MC	Q9JKB3	reviewed	YBOX3_MC	Y-box-bind	Ybx3 Csda	Mus muscı	361
PRKDC_M(	P97313	reviewed	PRKDC_M(	DNA-depe	Prkdc Xrcc	Mus muscı	4128
ADT4_MOL	Q3V132	reviewed	ADT4_MOL	ADP/ATP tr	Slc25a31 /	Mus muscı	320
NDKA_MOI	P15532	reviewed	NDKA_MOI	Nucleoside	Nme1 Nm2	Mus muscı	152
LZTL1_MOI	Q9JHQ5	reviewed	LZTL1_MOI	Leucine zip	Lztfl1	Mus muscı	299
COF1_MOI	P18760	reviewed	COF1_MOI	Cofilin-1 (C	Cfl1	Mus muscı	166
GLO2_MOI	Q99KB8	reviewed	GLO2_MOI	Hydroxyac	Hagh Glo2	Mus muscı	309
ANXA2_MC	P07356	reviewed	ANXA2_MC	Annexin A2	Anxa2 Anx2	Mus muscı	339
TCPA_MOL	P11983	reviewed	TCPA_MOL	T-complex	Tcp1 Cct1	Mus muscı	556
ATAD5_MC	Q4QY64	reviewed	ATAD5_MC	ATPase far	Atad5 Frag	Mus muscı	1826
IDHC_MOL	O88844	reviewed	IDHC_MOL	Isocitrate c	Idh1	Mus muscı	414

GOGA4_M(	Q91VW5	reviewed	GOGA4_M(	Golgin sub	Golga4	Mus muscı	2238
RUVB1_MC	P60122	reviewed	RUVB1_MC	RuvB-like 1	Ruvbl1 Tip	Mus muscı	456
DHB4_MOI	P51660	reviewed	DHB4_MOI	Peroxisom	Hsd17b4 E	Mus muscı	735
K1210_MC	E9Q0C6	reviewed	K1210_MC	Acrosomal	Kiaa1210 (	Mus muscı	1637
UN13C_M(	Q8K0T7	reviewed	UN13C_M(	Protein unc	Unc13c	Mus muscı	2210
DYHC1_M(	Q9JHU4	reviewed	DYHC1_M(	Cytoplasm	Dync1h1 D	Mus muscı	4644
ENPL_MOL	P08113	reviewed	ENPL_MOL	Endoplasm	Hsp90b1 G	Mus muscı	802
PCLO_MOI	Q9QYX7	reviewed	PCLO_MOI	Protein pic	Pclo Acz	Mus muscı	5068
ODP2_MOI	Q8BMF4	reviewed	ODP2_MOI	Dihydrolipo	Dlat	Mus muscı	642
SPEF2_MO	Q8C9J3	reviewed	SPEF2_MO	Sperm flag	Spef2 Kpl2	Mus muscı	1724
TIF1B_MOI	Q62318	reviewed	TIF1B_MOI	Transcripti	Trim28 Kap	Mus muscı	834
IIGP5_MOL	Q8C262	reviewed	IIGP5_MOL	Interferon-	Irgc Gm11(	Mus muscı	412
NUMA1_M(	E9Q7G0	reviewed	NUMA1_M(	Nuclear mi	Numa1	Mus muscı	2094
RL31_MOL	P62900	reviewed	RL31_MOL	Large ribos	Rpl31	Mus muscı	125
RL7A_MOL	P12970	reviewed	RL7A_MOL	Large ribos	Rpl7a Surf-	Mus muscı	266
ROP1L_MC	Q9EQ00	reviewed	ROP1L_MC	Ropporin-1	Ropn1l Asp	Mus muscı	218
TCPB_MOL	P80314	reviewed	TCPB_MOL	T-complex	Cct2 Cctb	Mus muscı	535
TCPQ_MOI	P42932	reviewed	TCPQ_MOI	T-complex	Cct8 Cctq	Mus muscı	548
PLCH1_MC	Q4KWH5	reviewed	PLCH1_MC	1-phospha	Plch1 Kiaa	Mus muscı	1682
SETD2_MC	E9Q5F9	reviewed	SETD2_MC	Histone-lys	Setd2 Kiaa	Mus muscı	2537
GOGA3_M(	P55937	reviewed	GOGA3_M(	Golgin sub	Golga3 Me	Mus muscı	1487
STIP1_MOL	Q60864	reviewed	STIP1_MOL	Stress-indu	Stip1	Mus muscı	543
KCY_MOUS	Q9DBP5	reviewed	KCY_MOUS	UMP-CMP	Cmpk1 Cm	Mus muscı	196
MARE1_M(	Q61166	reviewed	MARE1_M(	Microtubul	Mapre1	Mus muscı	268
PDIA3_MO	P27773	reviewed	PDIA3_MO	Protein dis	Pdia3 Erp E	Mus muscı	505
SUCB1_M(	Q9Z2I9	reviewed	SUCB1_M(	Succinate-	Sucla2	Mus muscı	463
CO6A5_M(	A6H584	reviewed	CO6A5_M(	Collagen a	Col6a5 Co	Mus muscı	2640
HXK1_MOL	P17710	reviewed	HXK1_MOL	Hexokinase	Hk1	Mus muscı	974
THOP1_M(	Q8C1A5	reviewed	THOP1_M(	Thimet olig	Thop1	Mus muscı	687
SODM_MO	P09671	reviewed	SODM_MO	Superoxide	Sod2 Sod-2	Mus muscı	222
SODC_MO	P08228	reviewed	SODC_MO	Superoxide	Sod1	Mus muscı	154
ATPG_MOL	Q91VR2	reviewed	ATPG_MOL	ATP syntha	Atp5f1c Atp	Mus muscı	298
ACYP1_MC	P56376	reviewed	ACYP1_MC	Acylphospl	Acyp1 Acyp	Mus muscı	99
ALBU_MOL	P07724	reviewed	ALBU_MOL	Albumin	Alb Alb-1 A	Mus muscı	608
ECHB_MOI	Q99JY0	reviewed	ECHB_MOI	Trifunction	Hadhb	Mus muscı	475
PLEC_MOL	Q9QXS1	reviewed	PLEC_MOL	Plectin (PC	Plec Plec1	Mus muscı	4691
RS4X_MOL	P62702	reviewed	RS4X_MOL	Small ribos	Rps4x Rps4	Mus muscı	263
RL13A_MO	P19253	reviewed	RL13A_MO	Large ribos	Rpl13a P19	Mus muscı	203
UB2L3_MC	P68037	reviewed	UB2L3_MC	Ubiquitin-c	Ube2l3 Ub	Mus muscı	154
MAT1_MOL	P51949	reviewed	MAT1_MOL	CDK-activa	Mnat1 Mat	Mus muscı	309
PDIA1_MO	P09103	reviewed	PDIA1_MO	Protein dis	P4hb Pdia1	Mus muscı	509
TKTL2_MOI	Q9D4D4	reviewed	TKTL2_MOI	Transketola	Tktl2	Mus muscı	627
1433E_MO	P62259	reviewed	1433E_MO	14-3-3 pro	Ywhae	Mus muscı	255
PABP1_MC	P29341	reviewed	PABP1_MC	Polyadenyl	Pabpc1 Pa	Mus muscı	636
HNRPM_M	Q9D0E1	reviewed	HNRPM_M	Heterogen	Hnrnpm Hı	Mus muscı	729
HSP7C_M(	P63017	reviewed	HSP7C_M(	Heat shock	Hspa8 Hsc	Mus muscı	646
YBOX2_MC	Q9Z2C8	reviewed	YBOX2_MC	Y-box-bind	Ybx2 Msy2	Mus muscı	360
DNJA1_MC	P63037	reviewed	DNJA1_MC	DnaJ homo	Dnaja1 Dn	Mus muscı	397

ADGB_MO	G3UZ78	reviewed	ADGB_MO	Androglobi	Adgb Capn	Mus muscu	1657
THIM_MOU	Q8BWT1	reviewed	THIM_MOU	3-ketoacyl-	Acaa2	Mus muscu	397
CACP_MO	P47934	reviewed	CACP_MO	Carnitine C	Crat	Mus muscu	626
FSIP2_MO	A2ARZ3	reviewed	FSIP2_MO	Fibrous sh	Fsip2	Mus muscu	6995
WDR62_M	Q3U3T8	reviewed	WDR62_M	WD repeat	Wdr62	Mus muscu	1523
PDIA6_MO	Q922R8	reviewed	PDIA6_MO	Protein dis	Pdia6 Txnd	Mus muscu	440
SAHH_MO	P50247	reviewed	SAHH_MO	Adenosylh	Ahcy	Mus muscu	432
HSP74_MC	Q61316	reviewed	HSP74_MC	Heat shock	Hspa4 Apg	Mus muscu	841
SYNE1_MC	Q6ZWR6	reviewed	SYNE1_MC	Nesprin-1 (	Syne1	Mus muscu	8799
DNJA2_MC	Q9QYJ0	reviewed	DNJA2_MC	DnaJ homo	Dnaja2	Mus muscu	412
DMD_MOU	P11531	reviewed	DMD_MOU	Dystrophin	Dmd	Mus muscu	3678
FABP9_MC	O08716	reviewed	FABP9_MC	Fatty acid-l	Fabp9 Perf	Mus muscu	132
1433F_MO	P68510	reviewed	1433F_MO	14-3-3 prot	Ywhah	Mus muscu	246
RS11_MOU	P62281	reviewed	RS11_MOU	Small ribos	Rps11	Mus muscu	158
PGAM2_M	O70250	reviewed	PGAM2_M	Phosphogl	Pgam2	Mus muscu	253
RANG_MO	P34022	reviewed	RANG_MO	Ran-specif	Ranbp1 Ht	Mus muscu	203
PSA2_MOU	P49722	reviewed	PSA2_MOU	Proteasom	Psma2 Lm	Mus muscu	234
PTMA_MO	P26350	reviewed	PTMA_MO	Prothymos	Ptma	Mus muscu	111
SPT20_MO	Q80YT5	reviewed	SPT20_MO	Spermatog	Spata20 Ti	Mus muscu	790
SMC2_MO	Q8CG48	reviewed	SMC2_MO	Structural	Smc2 Cap	Mus muscu	1191
SYDC_MO	Q922B2	reviewed	SYDC_MO	Aspartate--	Dars1 Dars	Mus muscu	501
IDH3A_MO	Q9D6R2	reviewed	IDH3A_MO	Isocitrate c	Idh3a	Mus muscu	366
ACTB_MOU	P60710	reviewed	ACTB_MOU	Actin, cyto	Actb	Mus muscu	375
BIP_MOUS	P20029	reviewed	BIP_MOUS	Endoplasm	Hspa5 Grp	Mus muscu	655
PLCE1_MC	Q8K4S1	reviewed	PLCE1_MC	1-phospha	Plce1 Kiaa	Mus muscu	2282
RPN1_MO	Q91YQ5	reviewed	RPN1_MO	Dolichyl-di	Rpn1	Mus muscu	608
INO1_MOU	Q9JHU9	reviewed	INO1_MOU	Inositol-3-p	Isyna1 Ino1	Mus muscu	557
UBE2N_M	P61089	reviewed	UBE2N_M	Ubiquitin-c	Ube2n Blu	Mus muscu	152
AGRV1_MC	Q8VHN7	reviewed	AGRV1_MC	Adhesion C	Adgrv1 Gpr	Mus muscu	6298
LRC46_MC	Q9DAP0	reviewed	LRC46_MC	Leucine-ric	Lrrc46	Mus muscu	323
HARS1_MC	Q61035	reviewed	HARS1_MC	Histidine--	Hars1 Hars	Mus muscu	509
PRS10_MC	P62334	reviewed	PRS10_MC	26S protea	Psmc6 Sug	Mus muscu	389
REV3L_MO	Q61493	reviewed	REV3L_MO	DNA polym	Rev3l Polz	Mus muscu	3122
LIS1_MOU	P63005	reviewed	LIS1_MOU	Platelet-ac	Pafah1b1 L	Mus muscu	410
BRD4_MO	Q9ESU6	reviewed	BRD4_MO	Bromodom	Brd4 Mcap	Mus muscu	1400
AFF4_MOU	Q9ESC8	reviewed	AFF4_MOU	AF4/FMR2	Aff4 Alf4	Mus muscu	1160
PRC2A_MC	Q7TSC1	reviewed	PRC2A_MC	Protein PR	Prrc2a Bat	Mus muscu	2158
KMT2A_MC	P55200	reviewed	KMT2A_MC	Histone-ly	Kmt2a All1	Mus muscu	3966
RS20_MOU	P60867	reviewed	RS20_MOU	Small ribos	Rps20	Mus muscu	119
KDM5B_M	Q80Y84	reviewed	KDM5B_M	Lysine-spe	Kdm5b Jari	Mus muscu	1544
BAZ2B_MC	A2AUY4	reviewed	BAZ2B_MC	Bromodom	Baz2b	Mus muscu	2123
DHX15_M	O35286	reviewed	DHX15_M	ATP-depen	Dhx15 Ddx	Mus muscu	795
SCAM2_M	Q9ERN0	reviewed	SCAM2_M	Secretory c	Scamp2	Mus muscu	329
SMC6_MO	Q924W5	reviewed	SMC6_MO	Structural	Smc6 Kiaa	Mus muscu	1097
DYH1_MO	E9Q8T7	reviewed	DYH1_MO	Dynein axo	Dnah1 Dhc	Mus muscu	4250
MYO9A_M	Q8C170	reviewed	MYO9A_M	Unconvent	Myo9a Myr	Mus muscu	2542
GSTA1_MC	P13745	reviewed	GSTA1_MC	Glutathion	Gsta1 Gsta	Mus muscu	223
PEPL_MOU	Q9R269	reviewed	PEPL_MOU	Periplakin	Ppl	Mus muscu	1755

TEX15_MO	F8VPN2	reviewed	TEX15_MO	Testis-expr	Tex15		Mus muscu	2785
CENPE_M(	Q6RT24	reviewed	CENPE_M(	Centromer	Cenpe		Mus muscu	2474
RL9_MOUS	P51410	reviewed	RL9_MOUS	Large ribos	Rpl9		Mus muscu	192
KMT2D_M(	Q6PDK2	reviewed	KMT2D_M(	Histone-lys	Kmt2d Mll2		Mus muscu	5588
RBBP6_MC	P97868	reviewed	RBBP6_MC	E3 ubiquiti	Rbbp6 P2p		Mus muscu	1790
PRS8_MOL	P62196	reviewed	PRS8_MOL	26S protea	Psmc5 Sug		Mus muscu	406
CNTLN_M(	A2AM05	reviewed	CNTLN_M(	Centlein (C	Cntln		Mus muscu	1397
MTUS1_M(	Q5HZI1	reviewed	MTUS1_M(	Microtubul	Mtus1 Atbp		Mus muscu	1210
PRPS1_MC	Q9D7G0	reviewed	PRPS1_MC	Ribose-pho	Prps1		Mus muscu	318
KHNYN_M(	Q80U38	reviewed	KHNYN_M(	Protein KH	Khnyn Kiaa		Mus muscu	671
BDP1_MOL	Q571C7	reviewed	BDP1_MOL	Transcripti	Bdp1 Kiaa1		Mus muscu	2467
MPRIP_MC	P97434	reviewed	MPRIP_MC	Myosin pho	Mprip Kiaa		Mus muscu	1024
CE126_MC	Q0VBV7	reviewed	CE126_MC	Centrosom	Cep126 Kia		Mus muscu	1103
GLRB_MOL	P48168	reviewed	GLRB_MOL	Glycine rec	Glrb		Mus muscu	496
IF122_MOL	Q6NWV3	reviewed	IF122_MOL	Intraflagell	Ift122 Wdr		Mus muscu	1182
PPP5_MOL	Q60676	reviewed	PPP5_MOL	Serine/thre	Ppp5c		Mus muscu	499
DYH5_MOL	Q8VHE6	reviewed	DYH5_MOL	Dynein axo	Dnah5 Dna		Mus muscu	4621
MABP1_M(	Q6NS57	reviewed	MABP1_M(	Mitogen-ac	Mapkbp1 J		Mus muscu	1503
CE162_MC	Q6ZQ06	reviewed	CE162_MC	Centrosom	Cep162 Kia		Mus muscu	1403
NSD2_MOL	Q8BVE8	reviewed	NSD2_MOL	Histone-lys	Nsd2 Kiaa1		Mus muscu	1365
SACS_MOL	Q9JLC8	reviewed	SACS_MOL	Sacsin (Dn	Sacs Kiaa0		Mus muscu	4582
PSA7_MOL	Q9Z2U0	reviewed	PSA7_MOL	Proteasom	Psma7		Mus muscu	248
PSMD1_M(	Q3TXS7	reviewed	PSMD1_M(	26S protea	Psmd1		Mus muscu	953
NP1L1_MC	P28656	reviewed	NP1L1_MC	Nucleoson	Nap1l1 Nrp		Mus muscu	391
CBP_MOUS	P45481	reviewed	CBP_MOUS	Histone lys	Crebbp Cb		Mus muscu	2441
RIMS1_MO	Q99NE5	reviewed	RIMS1_MO	Regulating	Rims1 Kiaa		Mus muscu	1463
MEIOC_MC	A2AG06	reviewed	MEIOC_MC	Meiosis-sp	Meioc Gm1		Mus muscu	965
RAG1_MOL	P15919	reviewed	RAG1_MOL	V(D)J recor	Rag1		Mus muscu	1040
GABT_MOL	P61922	reviewed	GABT_MOL	4-aminobu	Abat Gaba		Mus muscu	500
UTP20_MC	Q5XG71	reviewed	UTP20_MC	Small subu	Utp20 Drin		Mus muscu	2788
APC_MOUS	Q61315	reviewed	APC_MOUS	Adenomato	Apc		Mus muscu	2845
NDUS1_M(	Q91VD9	reviewed	NDUS1_M(	NADH-ubic	Ndufs1		Mus muscu	727
UBR4_MOL	A2AN08	reviewed	UBR4_MOL	E3 ubiquiti	Ubr4 Kiaa0		Mus muscu	5180
EBP2_MOL	Q9D903	reviewed	EBP2_MOL	Probable rI	Ebna1bp2		Mus muscu	306
ARC1B_M(	Q9WV32	reviewed	ARC1B_M(	Actin-relat	Arpc1b		Mus muscu	372
SP17_MOL	Q62252	reviewed	SP17_MOL	Sperm surf	Spa17 Sp1		Mus muscu	149
SYCP3_MC	P70281	reviewed	SYCP3_MC	Synaptone	Sycp3 Scp3		Mus muscu	254
INCE_MOL	Q9WU62	reviewed	INCE_MOL	Inner centr	Incenp		Mus muscu	880
SCPDL_M(	Q8R127	reviewed	SCPDL_M(	Saccharop	Sccpdh		Mus muscu	429
RS15A_MC	P62245	reviewed	RS15A_MC	Small ribos	Rps15a		Mus muscu	130
RENT1_MC	Q9EPU0	reviewed	RENT1_MC	Regulator c	Upf1 Rent1		Mus muscu	1124
RS9_MOUS	Q6ZWN5	reviewed	RS9_MOUS	Small ribos	Rps9		Mus muscu	194
FRAS1_MC	Q80T14	reviewed	FRAS1_MC	Extracellul	Fras1 Kiaa		Mus muscu	4010
GASP1_MC	Q5U4C1	reviewed	GASP1_MC	G-protein c	Gprasp1 Ki		Mus muscu	1347
PFKAP_MC	Q9WUA3	reviewed	PFKAP_MC	ATP-depen	Pfkp Pfkc		Mus muscu	784
SMCA4_M(	Q3TKT4	reviewed	SMCA4_M(	Transcripti	Smarca4 B		Mus muscu	1613
RHG31_M(	A6X8Z5	reviewed	RHG31_M(	Rho GTPas	Arhgap31 (		Mus muscu	1425
NIPBL_MO	Q6KCD5	reviewed	NIPBL_MO	Nipped-B-l	Nipbl Scc2		Mus muscu	2798

KAD2_MOU	Q9WTP6	reviewed	KAD2_MOU	Adenylate	Ak2	Mus muscu	239
SEPT2_MO	P42208	reviewed	SEPT2_MO	Septin-2 (N	Septin2 Ne	Mus muscu	361
CC85C_MO	E9Q6B2	reviewed	CC85C_MO	Coiled-coil	Ccdc85c	Mus muscu	420
RPTN_MOU	P97347	reviewed	RPTN_MOU	Repetin	Rptn	Mus muscu	1118
TBL2_MOU	Q9R099	reviewed	TBL2_MOU	Transducin	Tbl2	Mus muscu	442
PSMD3_MO	P14685	reviewed	PSMD3_MO	26S protea	Psmd3 P91	Mus muscu	530
NCOR1_MO	Q60974	reviewed	NCOR1_MO	Nuclear re	Ncor1 Rxrip	Mus muscu	2453
ICE1_MOU	E9Q286	reviewed	ICE1_MOU	Little elong	Ice1 Kiaa09	Mus muscu	2241
CFA47_MO	A0A0G2JEI	reviewed	CFA47_MO	Cilia and fl	Cfap47	Mus muscu	3184
BACH_MOU	Q91V12	reviewed	BACH_MOU	Cytosolic a	Acot7 Bach	Mus muscu	381
ANK2_MOU	Q8C8R3	reviewed	ANK2_MOU	Ankyrin-2 (	Ank2 AnkB	Mus muscu	3898
IQGA1_MO	Q9JKF1	reviewed	IQGA1_MO	Ras GTPas	Iqgap1	Mus muscu	1657
CEBPZ_MO	P53569	reviewed	CEBPZ_MO	CCAAT/enh	Cebpz Cbf	Mus muscu	1052
COX5B_MO	P19536	reviewed	COX5B_MO	Cytochrom	Cox5b	Mus muscu	128
PSMA8_MO	Q9CWH6	reviewed	PSMA8_MO	Proteasom	Psma8 Psm	Mus muscu	250
RTRAF_MO	Q9CQE8	reviewed	RTRAF_MO	RNA transc	RTRAF	Mus muscu	244
CTRO_MOU	P49025	reviewed	CTRO_MOU	Citron Rho	Cit Crik	Mus muscu	2055
PTPRC_MO	P06800	reviewed	PTPRC_MO	Receptor-t	Ptprc Ly-5	Mus muscu	1293
TEX9_MOU	Q9D845	reviewed	TEX9_MOU	Testis-expr	Tex9	Mus muscu	387
A1AT1_MO	P07758	reviewed	A1AT1_MO	Alpha-1-an	Serpina1a	Mus muscu	413
TPR_MOUS	F6ZDS4	reviewed	TPR_MOUS	Nucleopro	Tpr	Mus muscu	2431
VEGFD_MO	P97946	reviewed	VEGFD_MO	Vascular e	Vegfd Figf	Mus muscu	358
TLN2_MOU	Q71LX4	reviewed	TLN2_MOU	Talin-2	Tln2	Mus muscu	2375
GNPTA_MO	Q69ZN6	reviewed	GNPTA_MO	N-acetylglu	Gnptab Gn	Mus muscu	1235
PIWL2_MO	Q8CDG1	reviewed	PIWL2_MO	Piwi-like pr	Piwil2 Mili	Mus muscu	971
EEA1_MOU	Q8BL66	reviewed	EEA1_MOU	Early endo	Eea1	Mus muscu	1411
APOA1_MO	Q00623	reviewed	APOA1_MO	Apolipopro	Apoa1	Mus muscu	264
FRY_MOUS	E9Q8I9	reviewed	FRY_MOUS	Protein fur	Fry Kiaa41	Mus muscu	3020
THIL_MOU	Q8QZT1	reviewed	THIL_MOU	Acetyl-CoA	Acat1	Mus muscu	424
PKDRE_MO	Q9Z0T6	reviewed	PKDRE_MO	Polycystin	Pkdrej	Mus muscu	2126
BRCA2_MO	P97929	reviewed	BRCA2_MO	Breast can	Brca2 Fanc	Mus muscu	3329
KIRR3_MO	Q8BR86	reviewed	KIRR3_MO	Kin of IRRE	Kirrel3 Kiaa	Mus muscu	778
UCHL3_MO	Q9JKB1	reviewed	UCHL3_MO	Ubiquitin c	Uchl3	Mus muscu	230
HUWE1_M	Q7TMY8	reviewed	HUWE1_M	E3 ubiquiti	Huwe1 Kia	Mus muscu	4377
PEBP1_MO	P70296	reviewed	PEBP1_MO	Phosphatid	Pebp1 Pbp	Mus muscu	187
RN213_MO	E9Q555	reviewed	RN213_MO	E3 ubiquiti	Rnf213 My	Mus muscu	5148
PSD11_MO	Q8BG32	reviewed	PSD11_MO	26S protea	Psmd11	Mus muscu	422
CE350_MO	E9Q309	reviewed	CE350_MO	Centrosom	Cep350 Kia	Mus muscu	3095
ABCAD_MO	Q5SSE9	reviewed	ABCAD_MO	ATP-bindin	Abca13	Mus muscu	5034
ASH1L_MO	Q99MY8	reviewed	ASH1L_MO	Histone-lys	Ash1l	Mus muscu	2958
OBSCN_MO	A2AAJ9	reviewed	OBSCN_MO	Obscurin (I	Obscn Gm	Mus muscu	8886
DOCK7_MO	Q8R1A4	reviewed	DOCK7_MO	Dedicator c	Dock7 Gm	Mus muscu	2130
TM205_MO	Q91XE8	reviewed	TM205_MO	Transmem	Tmem205	Mus muscu	189
SMC4_MO	Q8CG47	reviewed	SMC4_MO	Structural	Smc4 Capc	Mus muscu	1286
UB2V1_MO	Q9CZY3	reviewed	UB2V1_MO	Ubiquitin-c	Ube2v1 Cr	Mus muscu	147
NPHN_MO	Q9QZS7	reviewed	NPHN_MO	Nephrin (R	Nphs1 Nph	Mus muscu	1256
SMC1A_MO	Q9CU62	reviewed	SMC1A_MO	Structural	Smc1a Sb1	Mus muscu	1233
PHIP_MOU	Q8VDD9	reviewed	PHIP_MOU	PH-interac	Phip Ndrp	Mus muscu	1821

PLMN_MOI	P20918	reviewed	PLMN_MOI	Plasminog	Plg	Mus muscu	812
FA8_MOUS	Q06194	reviewed	FA8_MOUS	Coagulatio	F8 Cf8 F8c	Mus muscu	2319
CDK12_MC	Q14AX6	reviewed	CDK12_MC	Cyclin-dep	Cdk12 Crk	Mus muscu	1484
PSB6_MOL	Q60692	reviewed	PSB6_MOL	Proteasom	Psmb6 Lm	Mus muscu	238
MYO7B_MC	Q99MZ6	reviewed	MYO7B_MC	Unconvent	Myo7b	Mus muscu	2113
MYCB2_MC	Q7TPH6	reviewed	MYCB2_MC	E3 ubiquiti	Mycbp2 Pa	Mus muscu	4749
ROCK1_MC	P70335	reviewed	ROCK1_MC	Rho-assoc	Rock1	Mus muscu	1354
ORC2_MOI	Q60862	reviewed	ORC2_MOI	Origin reco	Orc2 Orc2l	Mus muscu	576
RS27A_MC	P62983	reviewed	RS27A_MC	Ubiquitin-r	Rps27a Ub	Mus muscu	156
ERC2_MOL	Q6PH08	reviewed	ERC2_MOL	ERC protei	Erc2 Cast1	Mus muscu	957
SPG17_MC	Q5S003	reviewed	SPG17_MC	Sperm-ass	Spag17	Mus muscu	2320
APOB_MOI	E9Q414	reviewed	APOB_MOI	Apolipopro	Apob	Mus muscu	4505
CPLN1_MC	Q8CE72	reviewed	CPLN1_MC	Ciliogenes	Cplane1 Jb	Mus muscu	3214
TBA1A_MO	P68369	reviewed	TBA1A_MO	Tubulin alp	Tuba1a Tul	Mus muscu	451
AL1A1_MO	P24549	reviewed	AL1A1_MO	Aldehyde c	Aldh1a1 Al	Mus muscu	501
LUZP1_MC	Q8R4U7	reviewed	LUZP1_MC	Leucine zip	Luzp1 Luzp	Mus muscu	1068
FHAD1_MC	A6PWD2	reviewed	FHAD1_MC	Forkhead-a	Fhad1	Mus muscu	1420
THOC2_MC	B1AZI6	reviewed	THOC2_MC	THO comp	Thoc2	Mus muscu	1594
HYDIN_MC	Q80W93	reviewed	HYDIN_MC	Hydroceph	Hydin Hy3	Mus muscu	5154
CCD18_MC	Q640L5	reviewed	CCD18_MC	Coiled-coil	Ccdc18	Mus muscu	1455
MGAP_MO	A2AWL7	reviewed	MGAP_MO	MAX gene-a	Mga Kiaa42	Mus muscu	3003
AT2B4_MO	Q6Q477	reviewed	AT2B4_MO	Plasma me	Atp2b4	Mus muscu	1205
AFG32_MC	Q8JZQ2	reviewed	AFG32_MC	AFG3-like p	Afg3l2	Mus muscu	802
GVIN1_MO	Q80SU7	reviewed	GVIN1_MO	Interferon-	Gvin1	Mus muscu	2427
GAK_MOUS	Q99KY4	reviewed	GAK_MOUS	Cyclin-G-a	Gak	Mus muscu	1305
SPTN1_MC	P16546	reviewed	SPTN1_MC	Spectrin al	Sptan1 Spr	Mus muscu	2472
HNRPU_MC	Q8VEK3	reviewed	HNRPU_MC	Heterogen	Hnrnpu Hn	Mus muscu	800
MYH9_MOI	Q8VDD5	reviewed	MYH9_MOI	Myosin-9 ((	Myh9	Mus muscu	1960
DYH17_MC	Q69Z23	reviewed	DYH17_MC	Dynein axo	Dnah17 Dr	Mus muscu	4481
AKP13_MC	E9Q394	reviewed	AKP13_MC	A-kinase ai	Akap13 Br	Mus muscu	2776
P04258	P04258	reviewed	CO3A1_BC	Collagen a	COL3A1	Bos taurus	1049
AN32B_MC	Q9EST5	reviewed	AN32B_MC	Acidic leuc	Anp32b Pa	Mus muscu	272
GLU2B_MC	O08795	reviewed	GLU2B_MC	Glucosidas	Prkcsh	Mus muscu	521
CTTB2_MO	B9EJA2	reviewed	CTTB2_MO	Cortactin-b	Cttnbp2 Ki	Mus muscu	1648
SMG1_MO	Q8BKX6	reviewed	SMG1_MO	Serine/thre	Smg1 Atx K	Mus muscu	3658
TCPE_MOL	P80316	reviewed	TCPE_MOL	T-complex	Cct5 Ccte	Mus muscu	541
MESD_MO	Q9ERE7	reviewed	MESD_MO	LRP chape	Mesd Mesd	Mus muscu	224
GSTK1_MC	Q9DCM2	reviewed	GSTK1_MC	Glutathion	Gstk1	Mus muscu	226
PRDX6_MC	O08709	reviewed	PRDX6_MC	Peroxiredo	Prdx6 Aop2	Mus muscu	224
SRRM1_MC	Q52KI8	reviewed	SRRM1_MC	Serine/argi	Srrm1 Pop	Mus muscu	946
TMEDA_MC	Q9D1D4	reviewed	TMEDA_MC	Transmem	Tmed10 Tn	Mus muscu	219
RL7_MOUS	P14148	reviewed	RL7_MOUS	Large ribos	Rpl7	Mus muscu	270
TSGA8_MC	Q9JJL0	reviewed	TSGA8_MC	Testis-spec	Tsga8 Hala	Mus muscu	238
ZN292_MC	Q9Z2U2	reviewed	ZN292_MC	Zinc finger	Zfp292 Zfp	Mus muscu	2698
CAP1_MOL	P40124	reviewed	CAP1_MOL	Adenylyl cy	Cap1 Cap	Mus muscu	474
UBR5_MOL	Q80TP3	reviewed	UBR5_MOL	E3 ubiquiti	Ubr5 Edd E	Mus muscu	2792
STK10_MO	O55098	reviewed	STK10_MO	Serine/thre	Stk10 Lok	Mus muscu	966
EFTU_MOL	Q8BFR5	reviewed	EFTU_MOL	Elongation	Tufm	Mus muscu	452

S23IP_MOI	Q6NZC7	reviewed	S23IP_MOI	SEC23-inte	Sec23ip	Mus muscı	998
DBLOH_M(	Q9JIQ3	reviewed	DBLOH_M(	Diablo IAP-	Diablo Sma	Mus muscı	237
SETBP_MO	Q9Z180	reviewed	SETBP_MO	SET-bindin	Setbp1 Kia	Mus muscı	1582
ATM_MOUS	Q62388	reviewed	ATM_MOUS	Serine-pro	Atm	Mus muscı	3066
SDHA_MOI	Q8K2B3	reviewed	SDHA_MOI	Succinate	Sdha	Mus muscı	664
RYR2_MOL	E9Q401	reviewed	RYR2_MOL	Ryanodine	Ryr2	Mus muscı	4966
PHB2_MOI	O35129	reviewed	PHB2_MOI	Prohibitin-:	Phb2 Bap E	Mus muscı	299
ADT2_MOL	P51881	reviewed	ADT2_MOL	ADP/ATP tr	Slc25a5 Aa	Mus muscı	298
RB11A_MC	P62492	reviewed	RB11A_MC	Ras-relate	Rab11a Ra	Mus muscı	216
LDHA_MOI	P06151	reviewed	LDHA_MOI	L-lactate d	Ldha Ldh-1	Mus muscı	332
ROP1_MOI	Q9ESG2	reviewed	ROP1_MOI	Ropporin-1	Ropn1	Mus muscı	212
PCNT_MOI	P48725	reviewed	PCNT_MOI	Pericentrin	Pcnt Pcnt2	Mus muscı	2898
RALYL_MO	Q8BTF8	reviewed	RALYL_MO	RNA-bindir	Ralyl	Mus muscı	293
MPH6_MO	Q9D1Q1	reviewed	MPH6_MO	M-phase pl	Mphosph6	Mus muscı	161
EFCB5_MC	A0JP43	reviewed	EFCB5_MC	EF-hand ca	Efcab5	Mus muscı	1406
PCGF1_M(	Q8R023	reviewed	PCGF1_M(	Polycomb ;	Pcgf1 Nspc	Mus muscı	259
MAP1B_M(	P14873	reviewed	MAP1B_M(	Microtubul	Map1b Mta	Mus muscı	2464
MTG16_M(	O54972	reviewed	MTG16_M(	Protein CB	Cbfa2t3 Cl	Mus muscı	620
SHOT1_M(	Q8K2Q9	reviewed	SHOT1_M(	Shootin-1 (	Shtn1 Kiaa	Mus muscı	631
AT1A1_MO	Q8VDN2	reviewed	AT1A1_MO	Sodium/pc	Atp1a1	Mus muscı	1023
H3C_MOU	P02301	reviewed	H3C_MOU	Histone H3	H3-5 Gm14	Mus muscı	136
KI3L1_MOI	P83555	reviewed	KI3L1_MOI	Killer cell ir	Kir3dl1	Mus muscı	432
VIP2_MOU	Q6ZQB6	reviewed	VIP2_MOU	Inositol he:	Ppip5k2 Hi	Mus muscı	1129
LRIQ3_MO	Q14DL3	reviewed	LRIQ3_MO	Leucine-ric	Lrriq3 Lrrc4	Mus muscı	633
ESPL1_MO	P60330	reviewed	ESPL1_MO	Separin (E(	Espl1 Esp1	Mus muscı	2118
SAHH2_M(	Q80SW1	reviewed	SAHH2_M(	S-adenosy	Ahcyl1 Irbi	Mus muscı	530
EHD1_MOI	Q9WVK4	reviewed	EHD1_MOI	EH domain	Ehd1 Past1	Mus muscı	534
CK5P2_MC	Q8K389	reviewed	CK5P2_MC	CDK5 regu	Cdk5rap2 I	Mus muscı	1822
SUN2_MOI	Q8BJS4	reviewed	SUN2_MOI	SUN doma	Sun2 Unc8	Mus muscı	731
DDX46_M(	Q569Z5	reviewed	DDX46_M(	Probable A	Ddx46 Kiaa	Mus muscı	1032
FRPD1_MC	A2AKB4	reviewed	FRPD1_MC	FERM and I	Frmpd1 Kia	Mus muscı	1549
DEN2A_M(	Q8C4S8	reviewed	DEN2A_M(	DENN dom	Dennd2a	Mus muscı	1000
UT14B_MC	Q6EJB6	reviewed	UT14B_MC	U3 small n	Utp14b Jsc	Mus muscı	756
EPHA6_M(	Q62413	reviewed	EPHA6_M(	Ephrin type	Epha6 Ehk	Mus muscı	1035
MAP1A_M(	Q9QYR6	reviewed	MAP1A_M(	Microtubul	Map1a Mta	Mus muscı	2776
FANCI_MO	Q8K368	reviewed	FANCI_MO	Fanconi an	Fanci	Mus muscı	1330
UBA1_MOI	Q02053	reviewed	UBA1_MOI	Ubiquitin-l	Uba1 Sbx U	Mus muscı	1058
TCP4_MOL	P11031	reviewed	TCP4_MOL	Activated F	Sub1 Pc4 F	Mus muscı	127
RS5_MOUS	P97461	reviewed	RS5_MOUS	Small ribos	Rps5	Mus muscı	204
DGKH_MO	D3YXJ0	reviewed	DGKH_MO	Diacylglyce	Dgkh	Mus muscı	1211
DEND3_M(	A2RT67	reviewed	DEND3_M(	DENN dom	Dennd3 Kia	Mus muscı	1274
WDR76_M(	A6PWY4	reviewed	WDR76_M(	WD repeat	Wdr76	Mus muscı	622
ZCPW1_M(	Q6IR42	reviewed	ZCPW1_M(	Zinc finger	Zcwpw1 Gı	Mus muscı	630
TTF2_MOU	Q5NC05	reviewed	TTF2_MOU	Transcripti	Ttf2	Mus muscı	1138
KMT5A_MC	Q2YDW7	reviewed	KMT5A_MC	N-lysine m	Kmt5a Setc	Mus muscı	349
I12R1_MOI	Q60837	reviewed	I12R1_MOI	Interleukin	Il12rb1 Il12	Mus muscı	738
GRDN_MO	Q5SNZ0	reviewed	GRDN_MO	Girdin (Akt	Ccdc88a G	Mus muscı	1873
RL15_MOL	Q9CZM2	reviewed	RL15_MOL	Large ribos	Rpl15	Mus muscı	204

MED13_MO	Q5SWW4	reviewed	MED13_MO	Mediator o	Med13 Kiaa	Mus muscu	2171
DYH12_MO	Q3V0Q1	reviewed	DYH12_MO	Dynein axo	Dnah12 Dn	Mus muscu	3086
KI16B_MOU	B1AVY7	reviewed	KI16B_MOU	Kinesin-like	Kif16b Kiaa	Mus muscu	1312
CING_MOU	P59242	reviewed	CING_MOU	Cingulin	Cgn Kiaa13	Mus muscu	1191
DZIP3_MO	Q7TPV2	reviewed	DZIP3_MO	E3 ubiquiti	Dzip3 Kiaa	Mus muscu	1204
WAPL_MOU	Q65Z40	reviewed	WAPL_MOU	Wings apar	Wapl Kiaa0	Mus muscu	1200
SMCA1_MO	Q6PGB8	reviewed	SMCA1_MO	Probable g	Smarca1 S	Mus muscu	1046
UN45B_MO	Q8CGY6	reviewed	UN45B_MO	Protein unc	Unc45b Cr	Mus muscu	931
SF3A1_MO	Q8K4Z5	reviewed	SF3A1_MO	Splicing fac	Sf3a1	Mus muscu	791
RAI1_MOU	Q61818	reviewed	RAI1_MOU	Retinoic ac	Rai1 Kiaa1	Mus muscu	1889
US6NL_MO	Q80XC3	reviewed	US6NL_MO	USP6 N-ter	Usp6nl Kia	Mus muscu	819
EIF3H_MO	Q91WK2	reviewed	EIF3H_MO	Eukaryotic	Eif3h Eif3s	Mus muscu	352
CLK1_MOU	P22518	reviewed	CLK1_MOU	Dual speci	Clk1 Clk St	Mus muscu	483
Q6KB66-1	Q6KB66	reviewed	K2C80_HU	Keratin, typ	KRT80 KB2	Homo sapi	452
TULP4_MO	Q9JIL5	reviewed	TULP4_MO	Tubby-rela	Tulp4 Tusp	Mus muscu	1547
FLNA_MOU	Q8BTM8	reviewed	FLNA_MOU	Filamin-A (	Flna Fln Fln	Mus muscu	2647
CCD93_MO	Q7TQK5	reviewed	CCD93_MO	Coiled-coil	Ccdc93	Mus muscu	629
LIMD2_MO	Q8BGB5	reviewed	LIMD2_MO	LIM domain	Limd2	Mus muscu	128
SCNAA_MO	Q6QIY3	reviewed	SCNAA_MO	Sodium ch	Scn10a Sn	Mus muscu	1958
AMOL1_MO	Q9D4H4	reviewed	AMOL1_MO	Angiomotin	Amotl1	Mus muscu	968
Q2KJ83	Q2KJ83	reviewed	CBPN_BOV	Carboxype	CPN1	Bos taurus	462
CLPX_MOU	Q9JHS4	reviewed	CLPX_MOU	ATP-depen	Clpx	Mus muscu	634
EHBP1_MO	Q69ZW3	reviewed	EHBP1_MO	EH domain	Ehbp1 Kiaa	Mus muscu	1231
DLG5_MOU	E9Q9R9	reviewed	DLG5_MOU	Disks large	Dlg5	Mus muscu	1921
EMAL5_MO	Q8BQM8	reviewed	EMAL5_MO	Echinoderm	Eml5	Mus muscu	1977
AMPL_MOU	Q9CPY7	reviewed	AMPL_MOU	Cytosol am	Lap3 Lape	Mus muscu	519
DIDO1_MO	Q8C9B9	reviewed	DIDO1_MO	Death-indu	Dido1 Datf	Mus muscu	2256
PDS5B_MO	Q4VA53	reviewed	PDS5B_MO	Sister chro	Pds5b Apri	Mus muscu	1446
CCD66_MO	Q6NS45	reviewed	CCD66_MO	Coiled-coil	Ccdc66	Mus muscu	935
ERLN1_MO	Q91X78	reviewed	ERLN1_MO	Erlin-1 (En	Erlin1 Keo4	Mus muscu	348
VIGLN_MO	Q8VDJ3	reviewed	VIGLN_MO	Vigilin (Hig	Hdlbp	Mus muscu	1268
HERC2_MO	Q4U2R1	reviewed	HERC2_MO	E3 ubiquiti	Herc2 Jdf2	Mus muscu	4836
5NTC_MOU	Q3V1L4	reviewed	5NTC_MOU	Cytosolic p	Nt5c2	Mus muscu	560
RL36A_MO	P83882	reviewed	RL36A_MO	Large ribos	Rpl36a Rpl	Mus muscu	106
RPC2_MOU	P59470	reviewed	RPC2_MOU	DNA-direct	Polr3b	Mus muscu	1133
SLAI1_MOU	Q68FF7	reviewed	SLAI1_MOU	SLAIN moti	Slain1	Mus muscu	579
RHGBA_MO	Q80Y19	reviewed	RHGBA_MO	Rho GTPas	Arhgap11a	Mus muscu	987
L2GL2_MO	Q3TJ91	reviewed	L2GL2_MO	LLGL scribl	Llgl2 Llglh2	Mus muscu	1027
DZIP1_MO	Q8BMD2	reviewed	DZIP1_MO	Cilium ass	Dzip1 Kiaa	Mus muscu	852
ABCA3_MO	Q8R420	reviewed	ABCA3_MO	Phospholip	Abca3	Mus muscu	1704
BAZ1B_MO	Q9Z277	reviewed	BAZ1B_MO	Tyrosine-p	Baz1b Wbs	Mus muscu	1479
DNAI3_MO	B2RY71	reviewed	DNAI3_MO	Dynein axo	Dnai3 Wdr	Mus muscu	923
H12_MOUS	P15864	reviewed	H12_MOUS	Histone H1	H1-2 H1f2	Mus muscu	212
TRRAP_MO	Q80YV3	reviewed	TRRAP_MO	Transforma	Trrap	Mus muscu	2565
CAPS2_MO	Q8BYR5	reviewed	CAPS2_MO	Calcium-de	Cadps2 Ca	Mus muscu	1297
TEKT5_MO	G5E8A8	reviewed	TEKT5_MO	Tektin-5	Tekt5	Mus muscu	557
RPA1_MOU	O35134	reviewed	RPA1_MOU	DNA-direct	Polr1a Rpa	Mus muscu	1717
NFX1_MOU	B1AY10	reviewed	NFX1_MOU	Transcripti	Nfx1	Mus muscu	1114

SCOT1_MO	Q9D0K2	reviewed	SCOT1_MO	Succinyl-C	Oxct1 Oxct	Mus muscu	520
CTNA1_MO	P26231	reviewed	CTNA1_MO	Catenin alp	Ctnna1 Ca	Mus muscu	906
KCNQ2_MO	Q9Z351	reviewed	KCNQ2_MO	Potassium	Kcnq2 Kqt2	Mus muscu	759
PLVAP_MO	Q91VC4	reviewed	PLVAP_MO	Plasmalem	Plvap Pv1	Mus muscu	438
XPO2_MOU	Q9ERK4	reviewed	XPO2_MOU	Exportin-2	Cse1l Xpo2	Mus muscu	971
SPTB2_MO	Q62261	reviewed	SPTB2_MO	Spectrin be	Sptbn1 Elf	Mus muscu	2363
MYO5B_MO	P21271	reviewed	MYO5B_MO	Unconvent	Myo5b Kiaa	Mus muscu	1818
LARP1_MO	Q6ZQ58	reviewed	LARP1_MO	La-related	Larp1 Kiaa	Mus muscu	1072
INT6_MOU	Q6PCM2	reviewed	INT6_MOU	Integrator c	Ints6 Dbi1	Mus muscu	883
MED14_MO	A2ABV5	reviewed	MED14_MO	Mediator o	Med14 Crs	Mus muscu	1459
SMKZ_MOU	Q8C0N0	reviewed	SMKZ_MOU	Sperm mot	Gm4922	Mus muscu	497
SPT2_MOU	Q68FG3	reviewed	SPT2_MOU	Protein SPT	Spty2d1	Mus muscu	682
BRWD1_M	Q921C3	reviewed	BRWD1_M	Bromodom	Brwd1 Wdr	Mus muscu	2304
GCN1_MO	E9PVA8	reviewed	GCN1_MO	Stalled ribo	Gcn1 Gcn1	Mus muscu	2671
HELZ2_MO	E9QAM5	reviewed	HELZ2_MO	3'-5' exorib	Helz2	Mus muscu	2947
RN19B_MO	A2A7Q9	reviewed	RN19B_MO	E3 ubiquiti	Rnf19b Ibr	Mus muscu	732
M4K1_MOU	P70218	reviewed	M4K1_MOU	Mitogen-ac	Map4k1 Hp	Mus muscu	827
CMYA5_MO	Q70KF4	reviewed	CMYA5_MO	Cardiomyo	Cmya5 Sr5	Mus muscu	3739
ANR26_MO	Q811D2	reviewed	ANR26_MO	Ankyrin rep	Ankrd26 Ki	Mus muscu	1581
PA24B_MO	P0C871	reviewed	PA24B_MO	Cytosolic p	Pla2g4b	Mus muscu	782
TULP1_MO	Q9Z273	reviewed	TULP1_MO	Tubby-rela	Tulp1	Mus muscu	543
GON4L_MO	Q9DB00	reviewed	GON4L_MO	GON-4-like	Gon4l Gon	Mus muscu	2260
TM87A_MO	Q8BXN9	reviewed	TM87A_MO	Transmem	Tmem87a	Mus muscu	555
PHLP1_MO	Q8CHE4	reviewed	PHLP1_MO	PH domain	Phlpp1 Kia	Mus muscu	1687
DNMT1_MO	P13864	reviewed	DNMT1_MO	DNA (cytos	Dnmt1 Dnr	Mus muscu	1620
NINL_MOU	Q6ZQ12	reviewed	NINL_MOU	Ninein-like	Ninl Kiaa09	Mus muscu	1394
NCAM2_MO	O35136	reviewed	NCAM2_MO	Neural cell	Ncam2 Oc	Mus muscu	837
DHX30_MO	Q99PU8	reviewed	DHX30_MO	ATP-depen	Dhx30 Help	Mus muscu	1217
ZN706_MO	Q9D115	reviewed	ZN706_MO	Zinc finger	Znf706 Zfp	Mus muscu	76
JARD2_MO	Q62315	reviewed	JARD2_MO	Protein Jun	Jarid2 Jmj	Mus muscu	1234
TOP2B_MO	Q64511	reviewed	TOP2B_MO	DNA topois	Top2b	Mus muscu	1612
RS6_MOUS	P62754	reviewed	RS6_MOUS	Small ribos	Rps6	Mus muscu	249
PARP1_MO	P11103	reviewed	PARP1_MO	Poly [ADP-r	Parp1 Adpr	Mus muscu	1013
PPIA_MOU	P17742	reviewed	PPIA_MOU	Peptidyl-pr	Ppia	Mus muscu	164
RPGR1_MO	Q9EPQ2	reviewed	RPGR1_MO	X-linked re	Rpgrip1	Mus muscu	1331
RHG35_MO	Q91YM2	reviewed	RHG35_MO	Rho GTPas	Arhgap35 (	Mus muscu	1499
BIG1_MOU	G3X9K3	reviewed	BIG1_MOU	Brefeldin A	Arfgef1	Mus muscu	1846
LRC40_MO	Q9CRC8	reviewed	LRC40_MO	Leucine-ric	Lrrc40	Mus muscu	602
UN45A_MO	Q99KD5	reviewed	UN45A_MO	Protein unc	Unc45a Sn	Mus muscu	944
RBP1_MOU	Q62172	reviewed	RBP1_MOU	RalA-bindin	Ralbp1 Rip	Mus muscu	648
ZN609_MO	Q8BZ47	reviewed	ZN609_MO	Zinc finger	Znf609 Kia	Mus muscu	1413
APC7_MOU	Q9WVM3	reviewed	APC7_MOU	Anaphase-	Anapc7 Ap	Mus muscu	565
CC178_MO	Q8CDV0	reviewed	CC178_MO	Coiled-coil	Ccdc178	Mus muscu	866
LATS1_MO	Q8BYR2	reviewed	LATS1_MO	Serine/thre	Lats1 Wart	Mus muscu	1129
FYV1_MOU	Q9Z1T6	reviewed	FYV1_MOU	1-phospha	Pikfyve Fab	Mus muscu	2097
ZFAT_MOU	Q7TS63	reviewed	ZFAT_MOU	Zinc finger	Zfat Gm92	Mus muscu	1237
PIPNA_MO	P53810	reviewed	PIPNA_MO	Phosphatic	Pitpna Pitp	Mus muscu	271
AF9_MOUS	A2AM29	reviewed	AF9_MOUS	Protein AF-	Mllt3 Af9	Mus muscu	569

SYTL2_MO	Q99N50	reviewed	SYTL2_MO	Synaptotag	Sytl2 Slp2	Mus muscu	950
ZW10_MOI	O54692	reviewed	ZW10_MOI	Centromer	Zw10	Mus muscu	779
RL38_MOU	Q9JJI8	reviewed	RL38_MOU	Large ribos	Rpl38	Mus muscu	70
LC7L2_MO	Q7TNC4	reviewed	LC7L2_MO	Putative RN	Luc7l2	Mus muscu	392
ALPK3_MO	Q924C5	reviewed	ALPK3_MO	Alpha-prot	Alpk3 Kiaa	Mus muscu	1680
UACA_MOI	Q8CGB3	reviewed	UACA_MOI	Uveal auto	Uaca Kiaa1	Mus muscu	1411
ERLN2_MC	Q8BFZ9	reviewed	ERLN2_MC	Erlin-2 (En	Erlin2 Spfh	Mus muscu	340
K0825_MO	Q3UPC7	reviewed	K0825_MO	Uncharact		Mus muscu	1272
SHP1L_MC	Q3TTP0	reviewed	SHP1L_MC	Testicular	Shcbp1l	Mus muscu	639
TNR6C_MC	Q3UHC0	reviewed	TNR6C_MC	Trinucleoti	Tnrc6c Kia	Mus muscu	1690
RESF1_MO	Q5DTW7	reviewed	RESF1_MC	Retroelem	Resf1 Kiaa	Mus muscu	1521
SAFB2_MC	Q80YR5	reviewed	SAFB2_MC	Scaffold at	Safb2	Mus muscu	991
STRBP_MC	Q91WM1	reviewed	STRBP_MC	Spermatid	Strbp Spnr	Mus muscu	672
FAT3_MOU	Q8BNA6	reviewed	FAT3_MOU	Protocadh	Fat3 Gm11	Mus muscu	4555
SCN9A_MC	Q62205	reviewed	SCN9A_MC	Sodium ch	Scn9a Kiaa	Mus muscu	1984
WDR48_M	Q8BH57	reviewed	WDR48_M	WD repeat	Wdr48 Kia	Mus muscu	676
VP33B_MC	P59016	reviewed	VP33B_MC	Vacuolar p	Vps33b	Mus muscu	617
BAZ2A_MC	Q91YE5	reviewed	BAZ2A_MC	Bromodom	Baz2a Kiaa	Mus muscu	1889
AKAP1_MC	O08715	reviewed	AKAP1_MC	A-kinase a	Akap1 Aka	Mus muscu	857
ZFHX2_MC	Q2MHN3	reviewed	ZFHX2_MC	Zinc finger	Zfhx2 Kiaa	Mus muscu	2562
CP7B1_MC	Q60991	reviewed	CP7B1_MC	Cytochrom	Cyp7b1	Mus muscu	507
SAM9L_MC	Q69Z37	reviewed	SAM9L_MC	Sterile alpl	Samd9l Kia	Mus muscu	1561
CHD8_MO	Q09XV5	reviewed	CHD8_MO	Chromodo	Chd8 Kiaa	Mus muscu	2582
SPTB1_MO	P15508	reviewed	SPTB1_MO	Spectrin be	Sptb Spnb-	Mus muscu	2128
UIF_MOUS	Q91Z49	reviewed	UIF_MOUS	UAP56-inte	Fyttd1 Uif	Mus muscu	317
K2C1B_MC	Q6IFZ6	reviewed	K2C1B_MC	Keratin, typ	Krt77 Krt1b	Mus muscu	572
CCD73_MC	Q8CDM4	reviewed	CCD73_MC	Coiled-coil	Ccdc73	Mus muscu	1066
SPB1_MOU	Q9DBE9	reviewed	SPB1_MOU	pre-rRNA 2	Ftsj3	Mus muscu	838
RBM27_MC	Q5SFM8	reviewed	RBM27_MC	RNA-bindir	Rbm27 Kia	Mus muscu	1060
SBP1_MOU	P17563	reviewed	SBP1_MOU	Methaneth	Selenbp1 L	Mus muscu	472
AFF3_MOU	P51827	reviewed	AFF3_MOU	AF4/FMR2	Aff3 Laf4	Mus muscu	1254
MSH3_MO	P13705	reviewed	MSH3_MO	DNA mism	Msh3 Rep-	Mus muscu	1091
DEK_MOUS	Q7TNV0	reviewed	DEK_MOUS	Protein DE	Dek	Mus muscu	380
TCOF_MOU	O08784	reviewed	TCOF_MOU	Treacle pro	Tcof1	Mus muscu	1320
KANL3_MC	A2RSY1	reviewed	KANL3_MC	KAT8 regul	Kansl3 Kia	Mus muscu	903
F10A1_MO	Q99L47	reviewed	F10A1_MO	Hsc70-inte	St13 Fam1	Mus muscu	371
BPNT1_MC	Q9Z0S1	reviewed	BPNT1_MC	3'(2'),5'-bis	Bpnt1	Mus muscu	308
RL40_MOU	P62984	reviewed	RL40_MOU	Ubiquitin-r	Uba52 Ubc	Mus muscu	128
PSPC1_MC	Q8R326	reviewed	PSPC1_MC	Paraspeck	Pspc1 Psp	Mus muscu	523
2A5G_MOU	Q60996	reviewed	2A5G_MOU	Serine/thre	Ppp2r5c	Mus muscu	524
CCD13_MC	D3YV10	reviewed	CCD13_MC	Coiled-coil	Ccdc13	Mus muscu	709
NDUA7_MC	Q9Z1P6	reviewed	NDUA7_MC	NADH deh	Ndufa7	Mus muscu	113
JKIP3_MOU	Q5DTN8	reviewed	JKIP3_MOU	Janus kina	Jakmip3 Ki	Mus muscu	844
COPA_MOI	Q8CIE6	reviewed	COPA_MOI	Coatomer	Copa	Mus muscu	1224
CTCF_MOU	Q61164	reviewed	CTCF_MOU	Transcripti	Ctcf	Mus muscu	736
TET3_MOU	Q8BG87	reviewed	TET3_MOU	Methylcyto	Tet3	Mus muscu	1803
DYRK4_MC	Q8BI55	reviewed	DYRK4_MC	Dual speci	Dyrk4	Mus muscu	632
LRP1B_MC	Q9JI18	reviewed	LRP1B_MC	Low-densit	Lrp1b Lrpd	Mus muscu	4599

GA2L3_MC	Q3UWW6	reviewed	GA2L3_MC	GAS2-like p	Gas2l3	Mus muscu	683
GEMI5_MC	Q8BX17	reviewed	GEMI5_MC	Gem-assoc	Gemin5	Mus muscu	1502
CNTRL_MC	A2AL36	reviewed	CNTRL_MC	Centriolin (	Cntrl Cep1	Mus muscu	2334
SUCO_MO	Q8C341	reviewed	SUCO_MO	SUN doma	Suco Opt	Mus muscu	1250
GANP_MOI	Q9WUU9	reviewed	GANP_MOI	Germinal-c	Mcm3ap G	Mus muscu	1971
CC186_MC	Q8C9S4	reviewed	CC186_MC	Coiled-coil	Ccdc186 C	Mus muscu	917
CSN4_MOI	O88544	reviewed	CSN4_MOI	COP9 signa	Cops4 Csn	Mus muscu	406
S22AM_MC	Q8R0S9	reviewed	S22AM_MC	Solute carr	Slc22a22 (	Mus muscu	554
NBEA_MOI	Q9EPN1	reviewed	NBEA_MOI	Neurobeac	Nbea Lyst2	Mus muscu	2936
SAP18_MC	O55128	reviewed	SAP18_MC	Histone de	Sap18	Mus muscu	153
CO6A1_MC	Q04857	reviewed	CO6A1_MC	Collagen a	Col6a1	Mus muscu	1025
SAPC2_MC	Q9D818	reviewed	SAPC2_MC	Suppresso	Sapcd2	Mus muscu	391
PCM1_MO	Q9R0L6	reviewed	PCM1_MO	Pericentric	Pcm1	Mus muscu	2025
BCDO1_M	Q9JJS6	reviewed	BCDO1_M	Beta,beta-	Bco1 Bcdo	Mus muscu	566
CPIN1_MO	Q8WTY4	reviewed	CPIN1_MO	Anamorsin	Ciapin1	Mus muscu	309
NSE3_MOI	Q9CPR8	reviewed	NSE3_MOI	Non-struct	Nsmce3 M	Mus muscu	279
NSD3_MOI	Q6P2L6	reviewed	NSD3_MOI	Histone-lys	Nsd3 Whs	Mus muscu	1439
FANCM_M	Q8BGE5	reviewed	FANCM_M	Fanconi an	Fancm Kia	Mus muscu	2021
DEN4B_MC	Q3U1Y4	reviewed	DEN4B_MC	DENN dom	Dennd4b E	Mus muscu	1499
IQGA2_MC	Q3UQ44	reviewed	IQGA2_MC	Ras GTPas	Iqgap2	Mus muscu	1575
TBCD1_MC	Q60949	reviewed	TBCD1_MC	TBC1 doma	Tbc1d1 Kia	Mus muscu	1255
RBP2_MOI	Q9ERU9	reviewed	RBP2_MOI	E3 SUMO-p	Ranbp2	Mus muscu	3053
KIF23_MOI	E9Q5G3	reviewed	KIF23_MOI	Kinesin-like	Kif23	Mus muscu	953
FETUA_MC	P29699	reviewed	FETUA_MC	Alpha-2-HS	Ahsg Fetua	Mus muscu	345
CO4B_MOI	P01029	reviewed	CO4B_MOI	Compleme	C4b C4	Mus muscu	1738
CFA45_MC	Q9D9U9	reviewed	CFA45_MC	Cilia- and f	Cfap45 Cc	Mus muscu	551
INO80_MO	Q6ZPV2	reviewed	INO80_MO	Chromatin	Ino80 Inoc	Mus muscu	1559
VIP1_MOU	A2ARP1	reviewed	VIP1_MOU	Inositol he	Ppip5k1 Hi	Mus muscu	1436
IF140_MOI	E9PY46	reviewed	IF140_MOI	Intraflagell	Ift140 WD1	Mus muscu	1464
SNUT1_MC	Q9Z315	reviewed	SNUT1_MC	U4/U6.U5 t	Sart1 Haf	Mus muscu	806
APAF_MOL	O88879	reviewed	APAF_MOL	Apoptotic p	Apaf1	Mus muscu	1249
PLCB1_MC	Q9Z1B3	reviewed	PLCB1_MC	1-phospha	Plcb1 Plcb	Mus muscu	1216
PDE6A_MC	P27664	reviewed	PDE6A_MC	Rod cGMP-	Pde6a Mpa	Mus muscu	859
RYR1_MOL	E9PZQ0	reviewed	RYR1_MOL	Ryanodine	Ryr1	Mus muscu	5035
ZEP1_MOL	Q03172	reviewed	ZEP1_MOL	Zinc finger	Hivep1 Cry	Mus muscu	2688
EP15R_MC	Q60902	reviewed	EP15R_MC	Epidermal	Eps15l1 Ep	Mus muscu	907
ERCC6_MC	F8VPZ5	reviewed	ERCC6_MC	DNA excisi	Ercc6 Csb	Mus muscu	1481
FUMH_MO	P97807	reviewed	FUMH_MO	Fumarate h	Fh Fh1	Mus muscu	507
WASC4_M	Q3UMB9	reviewed	WASC4_M	WASH com	Washc4 Ki	Mus muscu	1173
TLL1_MOU	Q62381	reviewed	TLL1_MOU	Tolloid-like	Tll1 Tll	Mus muscu	1013
APCL_MOL	Q9Z1K7	reviewed	APCL_MOL	Adenomato	Apc2	Mus muscu	2274
CO6A2_MC	Q02788	reviewed	CO6A2_MC	Collagen a	Col6a2	Mus muscu	1034
IGFN1_MO	Q3KNY0	reviewed	IGFN1_MO	Immunoglo	Igfn1	Mus muscu	2849
TSP2_MOL	Q03350	reviewed	TSP2_MOL	Thrombosp	Thbs2 Tsp2	Mus muscu	1172
TEX10_MO	Q3URQ0	reviewed	TEX10_MO	Testis-expr	Tex10	Mus muscu	928
ARI4A_MO	F8VPQ2	reviewed	ARI4A_MO	AT-rich inte	Arid4a Rbb	Mus muscu	1261
FLIP1_MOI	Q9CS72	reviewed	FLIP1_MOI	Filamin-A-i	Filip1	Mus muscu	1214
PLSI_MOU	Q3V0K9	reviewed	PLSI_MOU	Plastin-1	Pls1	Mus muscu	630

HD_MOUS	P42859	reviewed	HD_MOUS	Huntingtin	Htt Hd Hdh	Mus muscu	3119
CMTA1_MC	A2A891	reviewed	CMTA1_MC	Calmoduli	Camta1 Kia	Mus muscu	1682
PAR3L_MC	Q9CSB4	reviewed	PAR3L_MC	Partitioning	Pard3b Als	Mus muscu	1203
BCAS3_MC	Q8CCN5	reviewed	BCAS3_MC	BCAS3 mic	Bcas3	Mus muscu	928
RADI_MOU	P26043	reviewed	RADI_MOU	Radixin (ES	Rdx	Mus muscu	583
ABCAC_MC	E9Q876	reviewed	ABCAC_MC	Glucosylce	Abca12	Mus muscu	2595
PMFBP_MC	Q9WVQ0	reviewed	PMFBP_MC	Polyamine	Pmfbp1 Sta	Mus muscu	1022
DLGP5_MC	Q8K4R9	reviewed	DLGP5_MC	Disks large	Dlgap5 Dlg	Mus muscu	808
OTU7B_MC	B2RUR8	reviewed	OTU7B_MC	OTU doma	Otud7b	Mus muscu	840
K1143_MO	Q8K039	reviewed	K1143_MO	Uncharacte		Mus muscu	155
MARF1_MC	Q8BJ34	reviewed	MARF1_MC	Meiosis reg	Marf1 Kiaa	Mus muscu	1730
NALCN_MC	Q8BXR5	reviewed	NALCN_MC	Sodium lea	Nalcn Vgcr	Mus muscu	1738
MA7D2_MC	A2AG50	reviewed	MA7D2_MC	MAP7 dom	Map7d2 M	Mus muscu	781
TEX11_MO	Q14AT2	reviewed	TEX11_MO	Testis-expr	Tex11 Zip4	Mus muscu	947
ZFHX4_MC	Q9JJN2	reviewed	ZFHX4_MC	Zinc finger	Zfhx4 Zfh4	Mus muscu	3550
CRCC2_MC	F6XLV1	reviewed	CRCC2_MC	Ciliary root	Crocc2	Mus muscu	1638
NWD1_MC	A6H603	reviewed	NWD1_MC	NACHT dor	Nwd1	Mus muscu	1563
PRDX3_MC	P20108	reviewed	PRDX3_MC	Thioredoxi	Prdx3 Aop1	Mus muscu	257
PHF2_MOU	Q9WTU0	reviewed	PHF2_MOU	Lysine-spe	Phf2 Kiaa0	Mus muscu	1096
AN34B_MC	Q3UUF8	reviewed	AN34B_MC	Ankyrin rep	Ankrd34b I	Mus muscu	508
UNC5C_M	O08747	reviewed	UNC5C_M	Netrin rece	Unc5c Rcn	Mus muscu	931
CH10_MOI	Q64433	reviewed	CH10_MOI	10 kDa hea	Hspe1	Mus muscu	102
HNRPL_MC	Q8R081	reviewed	HNRPL_MC	Heterogen	Hnrnpl Hnr	Mus muscu	586
TEFM_MOU	Q5SSK3	reviewed	TEFM_MOU	Transcripti	Tefm	Mus muscu	364
NAA16_MC	Q9DBB4	reviewed	NAA16_MC	N-alpha-ac	Naa16 Nar	Mus muscu	864
YTDC2_MC	B2RR83	reviewed	YTDC2_MC	3'-5' RNA h	Ythdc2	Mus muscu	1445
TALD3_MO	E9PV87	reviewed	TALD3_MO	Protein TAL	Talpid3	Mus muscu	1520
EME1_MOI	Q8BJW7	reviewed	EME1_MOI	Crossover	Eme1	Mus muscu	570
ANLN_MOI	Q8K298	reviewed	ANLN_MOI	Anillin	Anln	Mus muscu	1121
SPTA1_MO	P08032	reviewed	SPTA1_MO	Spectrin al	Spta1 Spna	Mus muscu	2415
SVIL_MOU	Q8K4L3	reviewed	SVIL_MOU	Supervillin	Svil	Mus muscu	2170
NUB1_MOI	P54729	reviewed	NUB1_MOI	NEDD8 ulti	Nub1 Nyre	Mus muscu	614
TOPB1_MC	Q6ZQF0	reviewed	TOPB1_MC	DNA topois	Topbp1 Kia	Mus muscu	1515
CC187_MC	Q8C5V8	reviewed	CC187_MC	Coiled-coil	Ccdc187	Mus muscu	958
UBP8_MOU	Q80U87	reviewed	UBP8_MOU	Ubiquitin c	Usp8 Kiaa(	Mus muscu	1080
CFA54_MC	Q8C6S9	reviewed	CFA54_MC	Cilia- and f	Cfap54	Mus muscu	3106
NOG2_MO	Q99LH1	reviewed	NOG2_MO	Nucleolar (	Gnl2	Mus muscu	728
BCLF1_MC	Q8K019	reviewed	BCLF1_MC	Bcl-2-asso	Bclaf1 Btf I	Mus muscu	919
ZN318_MC	Q99PP2	reviewed	ZN318_MC	Zinc finger	Znf318 Tzf	Mus muscu	2237
PRP4B_MC	Q61136	reviewed	PRP4B_MC	Serine/thre	Prpf4b Cbp	Mus muscu	1007
SC11A_MC	Q9R0P6	reviewed	SC11A_MC	Signal pept	Sec11a Se	Mus muscu	179
BGH3_MOI	P82198	reviewed	BGH3_MOI	Transformi	Tgfbi	Mus muscu	683
TGFB3_MC	P17125	reviewed	TGFB3_MC	Transformi	Tgfb3	Mus muscu	410
FREM1_MC	Q684R7	reviewed	FREM1_MC	FRAS1-rela	Frem1	Mus muscu	2191
CPSF2_MC	O35218	reviewed	CPSF2_MC	Cleavage a	Cpsf2 Cps	Mus muscu	782
PROF1_MC	P62962	reviewed	PROF1_MC	Profilin-1 (I	Pfn1	Mus muscu	140
VP13A_MC	Q5H8C4	reviewed	VP13A_MC	Intermemb	Vps13a Ch	Mus muscu	3166
TERB1_MO	Q8C0V1	reviewed	TERB1_MO	Telomere r	Terb1 Ccd	Mus muscu	768

CO4A3_M(	Q9QZS0	reviewed	CO4A3_M(	Collagen a	Col4a3	Mus muscı	1669
ADCY9_M(	P51830	reviewed	ADCY9_M(	Adenylate (	Adcy9	Mus muscı	1353
GSTP1_MC	P19157	reviewed	GSTP1_MC	Glutathion	Gstp1 Gstp	Mus muscı	210
PHIPL_MO	Q8BGT8	reviewed	PHIPL_MO	Phytanoyl-	Phyhipl	Mus muscı	375
CIP2A_MO	Q8BWY9	reviewed	CIP2A_MO	Protein CIF	Cip2a Kiaa	Mus muscı	907
IL18R_MOI	Q61098	reviewed	IL18R_MOI	Interleukin	Il18r1	Mus muscı	537
CCD80_M(	Q8R2G6	reviewed	CCD80_M(	Coiled-coil	Ccdc80 Ur	Mus muscı	949
DBIL5_MO	O09035	reviewed	DBIL5_MO	Diazepam-	Dbil5	Mus muscı	87
LCA5_MOI	Q80ST9	reviewed	LCA5_MOI	Lebercilin (	Lca5	Mus muscı	704
TMTC3_M(	Q8BRH0	reviewed	TMTC3_M(	Protein O-r	Tmtc3	Mus muscı	920
RWDD1_M	Q9CQK7	reviewed	RWDD1_M	RWD doma	Rwdd1 Dfr	Mus muscı	243
PAXB1_MC	P58501	reviewed	PAXB1_MC	PAX3- and	Paxbp1 Gc	Mus muscı	919
VCIP1_MO	Q8CDG3	reviewed	VCIP1_MO	Deubiquiti	Vcpip1 Vci	Mus muscı	1220
PA1B2_MC	Q61206	reviewed	PA1B2_MC	Platelet-ac	Pafah1b2 F	Mus muscı	229
SZT2_MOU	A2A9C3	reviewed	SZT2_MOU	KICSTOR c	Szt2	Mus muscı	3431
ERC6L_MC	Q8BHK9	reviewed	ERC6L_MC	DNA excisi	Ercc6l	Mus muscı	1240
MED1_MOI	Q925J9	reviewed	MED1_MOI	Mediator o	Med1 Crsp	Mus muscı	1575
PBX2_MOI	O35984	reviewed	PBX2_MOI	Pre-B-cell I	Pbx2	Mus muscı	430
LRBA_MOI	Q9ESE1	reviewed	LRBA_MOI	Lipopolysa	Lrba Bgl Lb	Mus muscı	2856
THIKA_MOI	Q921H8	reviewed	THIKA_MOI	3-ketoacyl-	Acaa1a Ac	Mus muscı	424
LKHA4_MC	P24527	reviewed	LKHA4_MC	Leukotrien	Lta4h	Mus muscı	611
FAN1_MOI	Q69ZT1	reviewed	FAN1_MOI	Fanconi-as	Fan1 Kiaa1	Mus muscı	1020
NAV3_MOI	Q80TN7	reviewed	NAV3_MOI	Neuron na	Nav3 Kiaa(	Mus muscı	2359
WFS1_MOI	P56695	reviewed	WFS1_MOI	Wolframin	Wfs1	Mus muscı	890
CO6A6_M(	Q8C6K9	reviewed	CO6A6_M(	Collagen a	Col6a6	Mus muscı	2265
ANR31_MC	A0A140LI8	reviewed	ANR31_MC	Ankyrin rep	Ankrd31	Mus muscı	1857
PDIP3_MO	Q8BG81	reviewed	PDIP3_MO	Polymeras	Poldip3	Mus muscı	420
TSP50_MO	Q8BLH5	reviewed	TSP50_MO	Probable tl	Prss50 Tsp	Mus muscı	439
NMUR1_M	O55040	reviewed	NMUR1_M	Neuromed	Nmur1 Gpı	Mus muscı	428
REXO4_MC	Q6PAQ4	reviewed	REXO4_MC	RNA exonu	Rexo4 Gm:	Mus muscı	432
ITPR1_MOI	P11881	reviewed	ITPR1_MOI	Inositol 1,4	Itpr1 Insp3	Mus muscı	2749
CIRBP_MO	P60824	reviewed	CIRBP_MO	Cold-induc	Cirbp Cirp	Mus muscı	172
GAS2_MOI	P11862	reviewed	GAS2_MOI	Growth arr	Gas2 Gas-:	Mus muscı	314
CDC5L_M(	Q6A068	reviewed	CDC5L_M(	Cell divisio	Cdc5l Kiaa	Mus muscı	802
GCC2_MO	Q8CHG3	reviewed	GCC2_MO	GRIP and c	Gcc2 Kiaa(	Mus muscı	1680
UNC80_M(	Q8BLN6	reviewed	UNC80_M(	Protein unc	Unc80 Kiaa	Mus muscı	3326
NEDD1_M(	P33215	reviewed	NEDD1_M(	Protein NE	Nedd1 Nec	Mus muscı	660
ANM9_MO	Q3U3W5	reviewed	ANM9_MO	Protein arg	Prmt9 Prm	Mus muscı	846
MORC3_M	F7BJB9	reviewed	MORC3_M	MORC fam	Morc3 Nxp	Mus muscı	942
HRH1_MOI	P70174	reviewed	HRH1_MOI	Histamine	Hrh1 Bphs	Mus muscı	488
CE170_MC	Q6A065	reviewed	CE170_MC	Centrosom	Cep170 Kia	Mus muscı	1588
ZN521_MC	Q6KAS7	reviewed	ZN521_MC	Zinc finger	Znf521 Evi:	Mus muscı	1311
TUT7_MOL	Q5BLK4	reviewed	TUT7_MOL	Terminal u	Tut7 Kiaa1	Mus muscı	1491
FAT4_MOU	Q2PZL6	reviewed	FAT4_MOU	Protocadh	Fat4 Fatj	Mus muscı	4981
PREX1_MC	Q69ZK0	reviewed	PREX1_MC	Phosphatic	Prex1 Kiaa	Mus muscı	1650
TARA_MOL	Q99KW3	reviewed	TARA_MOL	TRIO and F	Triobp Kiaa	Mus muscı	2014
KAPCA_MC	P05132	reviewed	KAPCA_MC	cAMP-depe	Prkaca Pka	Mus muscı	351
H2AW_MO	Q8CCK0	reviewed	H2AW_MO	Core histoı	Macroh2a2	Mus muscı	372

KCNH5_M(	Q920E3	reviewed	KCNH5_M(	Potassium	Kcnh5 Eag:	Mus muscı	988
DPOLQ_M(	Q8CGS6	reviewed	DPOLQ_M(	DNA polym	Polq Chao:	Mus muscı	2544
MYOME_M	Q80YT7	reviewed	MYOME_M	Myomegali	Pde4dip Ki	Mus muscı	2224
CDYL2_MC	Q9D5D8	reviewed	CDYL2_MC	Chromodo	Cdyl2	Mus muscı	503
FYCO1_MC	Q8VDC1	reviewed	FYCO1_MC	FYVE and c	Fyco1	Mus muscı	1437
RBM28_M(	Q8CGC6	reviewed	RBM28_M(	RNA-bindir	Rbm28	Mus muscı	750
MCM9_MC	Q2KHI9	reviewed	MCM9_MC	DNA helica	Mcm9 Mcn	Mus muscı	1134
CO4A4_M(	Q9QZR9	reviewed	CO4A4_M(	Collagen a	Col4a4	Mus muscı	1682
TEBP_MOL	Q9R0Q7	reviewed	TEBP_MOL	Prostaglan	Ptges3 Sid:	Mus muscı	160
ADT1_MOL	P48962	reviewed	ADT1_MOL	ADP/ATP tr	Slc25a4 Aa	Mus muscı	298
RED2_MOL	Q9JI20	reviewed	RED2_MOL	Double-str	Adarb2 Ada	Mus muscı	745
FKB1A_MC	P26883	reviewed	FKB1A_MC	Peptidyl-pr	Fkbp1a Fkl	Mus muscı	108
TT21A_MO	Q8C0S4	reviewed	TT21A_MO	Tetratricop	Ttc21a Thn	Mus muscı	1314
CDK13_M(	Q69ZA1	reviewed	CDK13_M(	Cyclin-dep	Cdk13 Cdc	Mus muscı	1511
RASD1_MC	O35626	reviewed	RASD1_MC	Dexametha	Rasd1 Dex	Mus muscı	280
SGO2_MOI	Q7TSY8	reviewed	SGO2_MOI	Shugoshin	Sgo2 Sgol2	Mus muscı	1164
IDHP_MOL	P54071	reviewed	IDHP_MOL	Isocitrate c	Idh2	Mus muscı	452
TSR1_MOL	Q5SWD9	reviewed	TSR1_MOL	Pre-rRNA-p	Tsr1 Kiaa1	Mus muscı	803
CCD71_M(	Q8VEG0	reviewed	CCD71_M(	Coiled-coil	Ccdc71	Mus muscı	433
CISD1_MO	Q91WS0	reviewed	CISD1_MO	CDGSH iro	Cisd1 D10	Mus muscı	108
E41L2_MO	O70318	reviewed	E41L2_MO	Band 4.1-li	Epb41l2 Ep	Mus muscı	988
SURF6_MC	P70279	reviewed	SURF6_MC	Surfeit locı	Surf6 Surf-	Mus muscı	355
RHG20_M(	Q6IFT4	reviewed	RHG20_M(	Rho GTPas	Arhgap20 I	Mus muscı	1182
ACOT9_MC	Q9R0X4	reviewed	ACOT9_MC	Acyl-coenz	Acot9 Acat	Mus muscı	439
PHF23_MC	Q8BSN5	reviewed	PHF23_MC	PHD finger	Phf23	Mus muscı	401
MAST4_MC	Q811L6	reviewed	MAST4_MC	Microtubul	Mast4	Mus muscı	2618
PACS1_MC	Q8K212	reviewed	PACS1_MC	Phosphofu	Pacs1	Mus muscı	961
P3C2A_MC	Q61194	reviewed	P3C2A_MC	Phosphatic	Pik3c2a Cp	Mus muscı	1686
PHB1_MOI	P67778	reviewed	PHB1_MOI	Prohibitin 1	Phb1 Phb	Mus muscı	272
SBNO1_M(	Q689Z5	reviewed	SBNO1_M(	Protein stra	Sbno1 Sno	Mus muscı	1390
3HIDH_MC	Q99L13	reviewed	3HIDH_MC	3-hydroxyis	Hibadh	Mus muscı	335
MPP8_MOI	Q3TYA6	reviewed	MPP8_MOI	M-phase pl	Mphosph8	Mus muscı	858
NCOR2_M(	Q9WU42	reviewed	NCOR2_M(	Nuclear rec	Ncor2 Smr	Mus muscı	2472
LRP2_MOL	A2ARV4	reviewed	LRP2_MOL	Low-densit	Lrp2	Mus muscı	4660
MA2A1_M(	P27046	reviewed	MA2A1_M(	Alpha-man	Man2a1 Ma	Mus muscı	1150
DLGP1_MC	Q9D415	reviewed	DLGP1_MC	Disks large	Dlgap1 Gka	Mus muscı	992
TCEA3_MC	P23881	reviewed	TCEA3_MC	Transcripti	Tcea3 Tfiis	Mus muscı	347
ABCA1_MC	P41233	reviewed	ABCA1_MC	Phospholip	Abca1 Abc	Mus muscı	2261
GNPAT_MC	P98192	reviewed	GNPAT_MC	Dihydroxya	Gnpat Dha	Mus muscı	678
USH1C_M(	Q9ES64	reviewed	USH1C_M(	Harmonin (	Ush1c	Mus muscı	910
UN13A_M(	Q4KUS2	reviewed	UN13A_M(	Protein unc	Unc13a	Mus muscı	1712
FNBP1_MC	Q80TY0	reviewed	FNBP1_MC	Formin-bin	Fnbp1 Fbp	Mus muscı	616
RL8_MOUS	P62918	reviewed	RL8_MOUS	Large ribos	Rpl8	Mus muscı	257
1433G_MC	P61982	reviewed	1433G_MC	14-3-3 prot	Ywhag	Mus muscı	247
NKTR_MOL	P30415	reviewed	NKTR_MOL	NK-tumor r	Nktr	Mus muscı	1453
STAR9_MO	Q80TF6	reviewed	STAR9_MO	StAR-relate	Stard9 Kiaa	Mus muscı	4561
ZN821_MC	Q6PD05	reviewed	ZN821_MC	Zinc finger	Znf821 Zfp	Mus muscı	413
WDR1_MO	O88342	reviewed	WDR1_MO	WD repeat	Wdr1	Mus muscı	606

FSTL5_MO	Q8BFR2	reviewed	FSTL5_MO	Follistatin-	Fstl5 Kiaa1	Mus muscı	847
TADBP_MC	Q921F2	reviewed	TADBP_MC	TAR DNA-b	Tardbp Tdp	Mus muscı	414
HAIR_MOU	Q61645	reviewed	HAIR_MOU	Lysine-spe	Hr	Mus muscı	1182
PYR1_MOL	B2RQC6	reviewed	PYR1_MOL	Multifuncti	Cad	Mus muscı	2225
SLF2_MOU	Q6P9P0	reviewed	SLF2_MOU	SMC5-SMC	Slf2 Fam17	Mus muscı	1278
ITPR2_MOI	Q9Z329	reviewed	ITPR2_MOI	Inositol 1,4	Itpr2 Itpr5	Mus muscı	2701
ARHG4_M(	Q7TNR9	reviewed	ARHG4_M(	Rho guanir	Arhgef4 Kia	Mus muscı	484
TGO1_MOI	Q8BI84	reviewed	TGO1_MOI	Transport a	Mia3 Kiaa0	Mus muscı	1930
CSF3_MOL	P09920	reviewed	CSF3_MOL	Granulocyt	Csf3 Csfg	Mus muscı	208
OXR1_MOI	Q4KMM3	reviewed	OXR1_MOI	Oxidation r	Oxr1 C7 Gr	Mus muscı	866
MRCKA_M(	Q3UU96	reviewed	MRCKA_M(	Serine/thre	Cdc42bpa	Mus muscı	1719
PHRF1_MC	A6H619	reviewed	PHRF1_MC	PHD and R	Phrf1 Kiaa1	Mus muscı	1682
VWA3A_M(	Q3UVV9	reviewed	VWA3A_M(	von Willebı	Vwa3a	Mus muscı	1148
PPR26_MC	Q6A025	reviewed	PPR26_MC	Protein pho	Ppp1r26 G	Mus muscı	1163
KCNH3_M(	Q9WVJ0	reviewed	KCNH3_M(	Potassium	Kcnh3 Elk2	Mus muscı	1095
ITSN2_MOI	Q9Z0R6	reviewed	ITSN2_MOI	Intersectin	Itsn2 Ese2	Mus muscı	1659
PTN14_MC	Q62130	reviewed	PTN14_MC	Tyrosine-pı	Ptpn14	Mus muscı	1189
RREB1_MC	Q3UH06	reviewed	RREB1_MC	Ras-respor	Rreb1	Mus muscı	1700
SF3B1_MC	Q99NB9	reviewed	SF3B1_MC	Splicing fa	Sf3b1 Sap1	Mus muscı	1304
H2AV_MOI	Q3THW5	reviewed	H2AV_MOI	Histone H2	H2az2 H2a	Mus muscı	128
AFF1_MOL	O88573	reviewed	AFF1_MOL	AF4/FMR2	Aff1 Mllt2 M	Mus muscı	1216
SASH1_MC	P59808	reviewed	SASH1_MC	SAM and S	Sash1	Mus muscı	1230
SHRM2_M(	A2ALU4	reviewed	SHRM2_M(	Protein Shı	Shroom2 A	Mus muscı	1481
KIF15_MOI	Q6P9L6	reviewed	KIF15_MOI	Kinesin-lik	Kif15 Klp2	Mus muscı	1387
CEFIP_MO	D3Z1D3	reviewed	CEFIP_MO	Cardiac-er	CEFIP	Mus muscı	1412
FIL1L_MOI	Q6P6L0	reviewed	FIL1L_MOI	Filamin A-i	Filip1l Doc	Mus muscı	1131
RIMS2_MO	Q9EQZ7	reviewed	RIMS2_MO	Regulating	Rims2 Rab	Mus muscı	1530
ROAA_MOI	Q99020	reviewed	ROAA_MOI	Heterogen	Hnrnpab C	Mus muscı	285
SPAG1_MC	Q80ZX8	reviewed	SPAG1_MC	Sperm-ass	Spag1 Tpis	Mus muscı	901
IGS10_MO	Q3V1M1	reviewed	IGS10_MO	Immunoglo	Igsf10	Mus muscı	2594
PSA3_MOL	O70435	reviewed	PSA3_MOL	Proteasom	Psma3	Mus muscı	255
ESF1_MOL	Q3V1V3	reviewed	ESF1_MOL	ESF1 homo	Esf1 Abtap	Mus muscı	845
MAP7_MOI	O88735	reviewed	MAP7_MOI	Ensconsin	Map7 Mtap	Mus muscı	730
SDCG8_M(	Q80UF4	reviewed	SDCG8_M(	Serologica	Sdccag8 C	Mus muscı	717
NCKX1_M(	Q91WD8	reviewed	NCKX1_M(	Sodium/pc	Slc24a1 Nc	Mus muscı	1130
PTPA_MOL	P58389	reviewed	PTPA_MOL	Serine/thre	Ptpa Ppp2ı	Mus muscı	323
CETN4_MC	Q8K4K1	reviewed	CETN4_MC	Centrin-4 (	Cetn4 Cen	Mus muscı	168
DACT2_MC	Q7TN08	reviewed	DACT2_MC	Dapper hoı	Dact2 Dpr2	Mus muscı	757
N6MT1_M(	Q6SKR2	reviewed	N6MT1_M(	Methyltran	N6amt1 He	Mus muscı	214
REV1_MOL	Q920Q2	reviewed	REV1_MOL	DNA repair	Rev1 Rev1l	Mus muscı	1249
CE112_MC	Q5PR68	reviewed	CE112_MC	Centrosom	Cep112 Cc	Mus muscı	954
CLAP1_MC	Q80TV8	reviewed	CLAP1_MC	CLIP-asso	Clasp1 Kia	Mus muscı	1535
ASXL3_MO	Q8C4A5	reviewed	ASXL3_MO	Putative Pc	Asxl3	Mus muscı	2259
SHIP1_MO	Q9ES52	reviewed	SHIP1_MO	Phosphatic	Inpp5d 7a3	Mus muscı	1191
NUSAP_M(	Q9ERH4	reviewed	NUSAP_M(	Nucleolar a	Nusap1	Mus muscı	427
RGPA1_MC	Q6GYP7	reviewed	RGPA1_MC	Ral GTPase	Ralgapa1 (	Mus muscı	2035
CSN1_MOI	Q99LD4	reviewed	CSN1_MOI	COP9 signa	Gps1 Cops	Mus muscı	471
PRS6A_MC	O88685	reviewed	PRS6A_MC	26S protea	Psmc3 Tbp	Mus muscı	442

UBC12_M(	P61082	reviewed	UBC12_M(	NEDD8-co	Ube2m Ub	Mus muscı	183
SL9A3_MO	G3X939	reviewed	SL9A3_MO	Sodium/hy	Slc9a3	Mus muscı	829
2AAB_MOL	Q7TNP2	reviewed	2AAB_MOL	Serine/thre	Ppp2r1b	Mus muscı	601
FLNC_MOL	Q8VHX6	reviewed	FLNC_MOL	Filamin-C (	Flnc Abpl F	Mus muscı	2726
HELZ_MOL	Q6DFV5	reviewed	HELZ_MOL	Probable h	Helz Kiaa0	Mus muscı	1964
RYR3_MOL	A2AGL3	reviewed	RYR3_MOL	Ryanodine	Ryr3	Mus muscı	4863
HFM1_MOI	D3Z4R1	reviewed	HFM1_MOI	Probable A	Hfm1	Mus muscı	1434
CUL3_MOL	Q9JLV5	reviewed	CUL3_MOL	Cullin-3 (C	Cul3	Mus muscı	768
CP096_MC	E9QMW4	reviewed	CP096_MC	Uncharacte		Mus muscı	1145
K1C9_MOL	Q6RHW0	reviewed	K1C9_MOL	Keratin, typ	Krt9 K9 Krt	Mus muscı	743
PSA5_MOL	Q9Z2U1	reviewed	PSA5_MOL	Proteasom	Psma5	Mus muscı	241
CAC1D_M(	Q99246	reviewed	CAC1D_M(	Voltage-de	Cacna1d C	Mus muscı	2179
SMC1B_M(	Q920F6	reviewed	SMC1B_M(	Structural ı	Smc1b Sm	Mus muscı	1248
DOCK9_M(	Q8BIK4	reviewed	DOCK9_M(	Dedicator (	Dock9 D14	Mus muscı	2055
NUCL_MOI	P09405	reviewed	NUCL_MOI	Nucleolin (	Ncl Nuc	Mus muscı	707
ZN516_MC	Q7TSH3	reviewed	ZN516_MC	Zinc finger	Znf516 Kia	Mus muscı	1157
ITA7_MOU!	Q61738	reviewed	ITA7_MOU!	Integrin alp	Itga7	Mus muscı	1179
RAI14_MOI	Q9EP71	reviewed	RAI14_MOI	Ankycorbir	Rai14 Kiaa	Mus muscı	979
CCNB3_M(	Q810T2	reviewed	CCNB3_M(	G2/mitotic	Ccnb3 Cyc	Mus muscı	1396
IF1AX_MOL	Q8BMJ3	reviewed	IF1AX_MOL	Eukaryotic	Eif1ax Eif1a	Mus muscı	144
PUS7L_MC	Q8CE46	reviewed	PUS7L_MC	Pseudouric	Pus7l	Mus muscı	702
CREL2_MC	Q9CYA0	reviewed	CREL2_MC	Protein dis	Creld2	Mus muscı	350
AMER1_M(	Q7TS75	reviewed	AMER1_M(	APC memb	Amer1 Fan	Mus muscı	1132
NF1_MOUS	Q04690	reviewed	NF1_MOUS	Neurofibro	Nf1	Mus muscı	2841
SR140_MC	Q6NV83	reviewed	SR140_MC	U2 snRNP-	U2surp Sr1	Mus muscı	1029
IF2P_MOU!	Q05D44	reviewed	IF2P_MOU!	Eukaryotic	Eif5b If2	Mus muscı	1216
SYPL1_MO	O09117	reviewed	SYPL1_MO	Synaptoph	Sypl1 Pphr	Mus muscı	261
DHX8_MOI	A2A4P0	reviewed	DHX8_MOI	ATP-depen	Dhx8	Mus muscı	1244
PPID_MOU	Q9CR16	reviewed	PPID_MOU	Peptidyl-pr	Ppid	Mus muscı	370
NOS2_MOI	P29477	reviewed	NOS2_MOI	Nitric oxide	Nos2 Inosl	Mus muscı	1144
ATS5_MOL	Q9R001	reviewed	ATS5_MOL	A disintegri	Adamts5	Mus muscı	930
HMCN1_M	D3YXG0	reviewed	HMCN1_M	Hemicentiı	Hmcn1	Mus muscı	5634
CD054_M(	E0CYV9	reviewed	CD054_M(	Uncharacte		Mus muscı	1786
QSER1_M(	A0A338P6I	reviewed	QSER1_M(	Glutamine	Qser1	Mus muscı	1788
IF4G1_MO	Q6NZJ6	reviewed	IF4G1_MO	Eukaryotic	Eif4g1	Mus muscı	1600
SRS10_MC	Q9R0U0	reviewed	SRS10_MC	Serine/argi	Srsf10 Fus	Mus muscı	262
AP3B1_MC	Q9Z1T1	reviewed	AP3B1_MC	AP-3 comp	Ap3b1	Mus muscı	1105
NEK1_MOL	P51954	reviewed	NEK1_MOL	Serine/thre	Nek1	Mus muscı	1203
WDFY3_M(	Q6VNB8	reviewed	WDFY3_M(	WD repeat	Wdfy3	Mus muscı	3508
FERM2_M(	Q8CIB5	reviewed	FERM2_M(	Fermitin fa	Fermt2 Ple	Mus muscı	680
DDX21_M(	Q9JIK5	reviewed	DDX21_M(	Nucleolar I	Ddx21	Mus muscı	851
NCHL1_M(	P70232	reviewed	NCHL1_M(	Neural cell	Chl1 Call	Mus muscı	1209
ZN786_MC	Q8BV42	reviewed	ZN786_MC	Zinc finger	Znf786 Zfp	Mus muscı	777
PDS5A_M(	Q6A026	reviewed	PDS5A_M(	Sister chro	Pds5a Kiaa	Mus muscı	1332
RBCC1_M(	Q9ESK9	reviewed	RBCC1_M(	RB1-induci	Rb1cc1 Cc	Mus muscı	1588
CAC1A_M(	P97445	reviewed	CAC1A_M(	Voltage-de	Cacna1a C	Mus muscı	2368
RRP5_MOL	Q6NS46	reviewed	RRP5_MOL	Protein RRI	Pdcd11 Alg	Mus muscı	1862
ZN445_MC	Q8R2V3	reviewed	ZN445_MC	Zinc finger	Znf445 Zfp	Mus muscı	986

WWC2_MO	Q6NXJ0	reviewed	WWC2_MO	Protein WW	Wwc2 D8E	Mus muscu	1187
NRIP1_MO	Q8CBD1	reviewed	NRIP1_MO	Nuclear rec	Nrip1	Mus muscu	1161
RS19_MOU	Q9CZX8	reviewed	RS19_MOU	Small ribos	Rps19	Mus muscu	145
RTF1_MOU	A2AQ19	reviewed	RTF1_MOU	RNA polym	Rtf1	Mus muscu	715
TDRD6_MO	P61407	reviewed	TDRD6_MO	Tudor dom	Tdrd6	Mus muscu	2134
PARVA_MO	Q9EPC1	reviewed	PARVA_MO	Alpha-parv	Parva Actp	Mus muscu	372
Z518B_MO	B2RRE4	reviewed	Z518B_MO	Zinc finger	Znf518b Ki	Mus muscu	1077
TNR6A_MO	Q3UHK8	reviewed	TNR6A_MO	Trinucleoti	Tnrc6a Kia	Mus muscu	1896
ZO2_MOUS	Q9Z0U1	reviewed	ZO2_MOUS	Tight juncti	Tjp2 Zo2	Mus muscu	1167
U17PA_MO	Q61068	reviewed	U17PA_MO	Ubiquitin c	Usp17la D	Mus muscu	526
TASO2_MO	Q5DTT3	reviewed	TASO2_MO	Protein TAS	Tasor2 Fan	Mus muscu	2382
CAND2_MO	Q6ZQ73	reviewed	CAND2_MO	Cullin-asso	Cand2 Kiaa	Mus muscu	1235
PB1_MOUS	Q8BSQ9	reviewed	PB1_MOUS	Protein pol	Pbrm1 Baf	Mus muscu	1634
TRIO_MOU	Q0KL02	reviewed	TRIO_MOU	Triple func	Trio	Mus muscu	3102
DC1L2_MO	Q6PDL0	reviewed	DC1L2_MO	Cytoplasm	Dync1li2 D	Mus muscu	492
OCRL_MOI	Q6NVF0	reviewed	OCRL_MOI	Inositol pol	Ocrl Ocrl1	Mus muscu	900
QCR1_MOI	Q9CZ13	reviewed	QCR1_MOI	Cytochrom	Uqcrc1	Mus muscu	480
AMOT_MOI	Q8VHG2	reviewed	AMOT_MOI	Angiomotir	Amot Kiaa1	Mus muscu	1126
CHD6_MO	A3KFM7	reviewed	CHD6_MO	Chromodo	Chd6 Kiaa1	Mus muscu	2711
CE295_MO	Q8BQ48	reviewed	CE295_MO	Centrosom	Cep295 Kia	Mus muscu	2412
UBF1_MOU	P25976	reviewed	UBF1_MOU	Nucleolar t	Ubtf Tcfub1	Mus muscu	765
ARG28_MO	P97433	reviewed	ARG28_MO	Rho guanir	Arhgef28 K	Mus muscu	1700
CC110_MO	Q3V125	reviewed	CC110_MO	Coiled-coil	Ccdc110 G	Mus muscu	848
ECHA_MOI	Q8BMS1	reviewed	ECHA_MOI	Trifunction	Hadha	Mus muscu	763
BLM_MOUS	O88700	reviewed	BLM_MOUS	RecQ-like I	Blm	Mus muscu	1416
TNC18_MO	Q80WC3	reviewed	TNC18_MO	Trinucleoti	Tnrc18 Kia	Mus muscu	2878
UBR1_MOU	O70481	reviewed	UBR1_MOU	E3 ubiquiti	Ubr1	Mus muscu	1757
TTLL4_MOI	Q80UG8	reviewed	TTLL4_MOI	Tubulin mc	Ttll4 Kiaa0	Mus muscu	1193
ZFP37_MO	P17141	reviewed	ZFP37_MO	Zinc finger	Zfp37 Zfp-3	Mus muscu	594
SPEG_MOU	Q62407	reviewed	SPEG_MOU	Striated mu	Speg Apeg	Mus muscu	3262
PRC2B_MO	Q7TPM1	reviewed	PRC2B_MO	Protein PRI	Prrc2b Bat	Mus muscu	1486
AL1B1_MO	Q9CZS1	reviewed	AL1B1_MO	Aldehyde d	Aldh1b1 Al	Mus muscu	519
CEP72_MO	Q9D3R3	reviewed	CEP72_MO	Centrosom	Cep72 Kiaa	Mus muscu	646
RECQ5_MO	Q8VID5	reviewed	RECQ5_MO	ATP-depen	Recql5	Mus muscu	982
CIC_MOUS	Q924A2	reviewed	CIC_MOUS	Protein cap	Cic Kiaa03	Mus muscu	2510
PLCB2_MO	A3KGF7	reviewed	PLCB2_MO	1-phospha	Plcb2	Mus muscu	1181
CLSPN_MO	Q80YR7	reviewed	CLSPN_MO	Claspin	Clspn	Mus muscu	1315
BICRA_MO	F8VPZ9	reviewed	BICRA_MO	BRD4-inter	Bicra Gltsc	Mus muscu	1578
GDN_MOU	Q07235	reviewed	GDN_MOU	Glia-derive	Serpine2 P	Mus muscu	397
KI26B_MOI	Q7TNC6	reviewed	KI26B_MOI	Kinesin-like	Kif26b	Mus muscu	2112
TRIPC_MO	G5E870	reviewed	TRIPC_MO	E3 ubiquiti	Trip12	Mus muscu	2025
H18_MOUS	Q8VIK3	reviewed	H18_MOUS	Histone H1	H1.8 H1f8	Mus muscu	304
MPI_MOUS	Q924M7	reviewed	MPI_MOUS	Mannose-6	Mpi Mpi1 P	Mus muscu	423
ATRX_MOU	Q61687	reviewed	ATRX_MOU	Transcripti	Atrx Hp1bp	Mus muscu	2476
UHRF1_MO	Q8VDF2	reviewed	UHRF1_MO	E3 ubiquiti	Uhrf1 Np95	Mus muscu	782
MYLK_MOU	Q6PDN3	reviewed	MYLK_MOU	Myosin ligh	Mylk	Mus muscu	1941
SRC8_MOU	Q60598	reviewed	SRC8_MOU	Src substra	Cttn Ems1	Mus muscu	546
AFAD_MOU	Q9QZQ1	reviewed	AFAD_MOU	Afadin (Afa	Afdn Af6 M	Mus muscu	1820

AF1L2_MO	Q5DTU0	reviewed	AF1L2_MO	Actin filam	Afap1l2 Kia	Mus muscu	825
SYVC_MOU	Q9Z1Q9	reviewed	SYVC_MOU	Valine--tRN	Vars1 Bat6	Mus muscu	1263
BAZ1A_MC	O88379	reviewed	BAZ1A_MC	Bromodom	Baz1a Cbp	Mus muscu	1555
FMN1_MOI	Q05860	reviewed	FMN1_MOI	Formin-1 (I	Fmn1 Fmn	Mus muscu	1466
BMP2K_M(	Q91Z96	reviewed	BMP2K_M(	BMP-2-ind	Bmp2k Bik	Mus muscu	1138
RL28_MOL	P41105	reviewed	RL28_MOL	Large ribos	Rpl28	Mus muscu	137
SLK_MOUS	O54988	reviewed	SLK_MOUS	STE20-like	Slk Kiaa02	Mus muscu	1233
MYO3A_M(	Q8K3H5	reviewed	MYO3A_M(	Myosin-IIIa	Myo3a	Mus muscu	1613
MTAP2_MC	P20357	reviewed	MTAP2_MC	Microtubul	Map2 Mtap	Mus muscu	1828
WNK3_MO	Q80XP9	reviewed	WNK3_MO	Serine/thre	Wnk3	Mus muscu	1757
TALDO_MC	Q93092	reviewed	TALDO_MC	Transaldol	Taldo1 Tal	Mus muscu	337
NKX11_MC	G3UXB3	reviewed	NKX11_MC	NK1 transc	Nkx1-1 Sax	Mus muscu	440
ZZZ3_MOU	Q6KAQ7	reviewed	ZZZ3_MOU	ZZ-type zin	Zzz3	Mus muscu	910
SP16H_MC	Q920B9	reviewed	SP16H_MC	FACT comp	Supt16h Fa	Mus muscu	1047
CA2D3_M(	Q9Z1L5	reviewed	CA2D3_M(	Voltage-de	Cacna2d3	Mus muscu	1091
CROCC_M	Q8CJ40	reviewed	CROCC_M	Rootletin ((	Crocc Kiaa	Mus muscu	2009
ITSN1_MOI	Q9Z0R4	reviewed	ITSN1_MOI	Intersectin	Itsn1 Ese1	Mus muscu	1714
CFA58_MC	B2RW38	reviewed	CFA58_MC	Cilia- and f	Cfap58	Mus muscu	873
P20L1_MO	Q8CCJ9	reviewed	P20L1_MO	PHD finger	Phf20l1	Mus muscu	1013
SPART_MC	Q8R1X6	reviewed	SPART_MC	Spartin	Spart Kiaa(	Mus muscu	671
NUTM1_M(	Q8BHP2	reviewed	NUTM1_M(	NUT family	Nutm1 Nut	Mus muscu	1126
PKP1_MOL	P97350	reviewed	PKP1_MOL	Plakophilir	Pkp1	Mus muscu	728
PLPL8_MO	Q8K1N1	reviewed	PLPL8_MO	Calcium-ir	Pnpla8 Ipla	Mus muscu	776
ITPR3_MOI	P70227	reviewed	ITPR3_MOI	Inositol 1,4	Itpr3	Mus muscu	2670
CBX3_MOI	P23198	reviewed	CBX3_MOI	Chromobo	Cbx3	Mus muscu	183
P02676	P02676	reviewed	FIBB_BOVI	Fibrinogen	FGB	Bos taurus	468
KINH_MOL	Q61768	reviewed	KINH_MOL	Kinesin-1 h	Kif5b Khcs	Mus muscu	963
CENPJ_MC	Q569L8	reviewed	CENPJ_MC	Centromer	Cenpj	Mus muscu	1344
BSN_MOU	O88737	reviewed	BSN_MOU	Protein bas	Bsn Kiaa04	Mus muscu	3942
WNT2B_M(	O70283	reviewed	WNT2B_M(	Protein Wn	Wnt2b Wn	Mus muscu	389
SEPT9_MO	Q80UG5	reviewed	SEPT9_MO	Septin-9 (S	Septin9 Kia	Mus muscu	583
DNPEP_M(	Q9Z2W0	reviewed	DNPEP_M(	Aspartyl ar	Dnpep	Mus muscu	473
SENP6_MC	Q6P7W0	reviewed	SENP6_MC	Sentrin-sp	Senp6 Kiaa	Mus muscu	1132
RGAP1_MC	Q9WVM1	reviewed	RGAP1_MC	Rac GTPas	Racgap1 M	Mus muscu	628
U520_MOL	Q6P4T2	reviewed	U520_MOL	U5 small n	Snrnp200 I	Mus muscu	2136
FGF8_MOL	P37237	reviewed	FGF8_MOL	Fibroblast	Fgf8 Aigf	Mus muscu	268
WNK1_MO	P83741	reviewed	WNK1_MO	Serine/thre	Wnk1 Hsn2	Mus muscu	2377
