# Supplementary material for: Testicular SIRT1 Loss Reveals an Aging‐Like Proteomic Landscape and Precipitates Reproductive Deterioration
Source: Andrology. 2026 Mar 12;14(6):1579–89. doi: 10.1111/andr.70201 (PMC13432521; doi:10.1111/andr.70201)
Supplement: Supplementary file 3 — Supporting File 3: andr70201‐sup‐0003‐DataS2.pdf [file ANDR-14-1579-s006.pdf]

From	Entry	Reviewed	Entry Nam	Protein nar	Gene Nam	Organism	Length
SMC3_MO	Q9CW03	reviewed	SMC3_MO	Structural	Smc3 Bam	Mus muscu	1217
RALYL_MO	Q8BTF8	reviewed	RALYL_MO	RNA-bindir	Ralyl	Mus muscu	293
MPH6_MO	Q9D1Q1	reviewed	MPH6_MO	M-phase p	Mphosph6	Mus muscu	161
EFCB5_MC	A0JP43	reviewed	EFCB5_MC	EF-hand ca	Efcab5	Mus muscu	1406
PCGF1_MC	Q8R023	reviewed	PCGF1_MC	Polycomb	Pcgf1 Nsp	Mus muscu	259
MAP1B_M	P14873	reviewed	MAP1B_M	Microtubul	Map1b Mta	Mus muscu	2464
MTG16_M	O54972	reviewed	MTG16_M	Protein CB	Cbfa2t3 Cb	Mus muscu	620
SHOT1_M	Q8K2Q9	reviewed	SHOT1_M	Shootin-1 (	Shtn1 Kiaa	Mus muscu	631
AT1A1_MO	Q8VDN2	reviewed	AT1A1_MO	Sodium/pc	Atp1a1	Mus muscu	1023
H3C_MOU	P02301	reviewed	H3C_MOU	Histone H3	H3-5 Gm1	Mus muscu	136
KI3L1_MO	P83555	reviewed	KI3L1_MO	Killer cell i	Kir3dl1	Mus muscu	432
VIP2_MOU	Q6ZQB6	reviewed	VIP2_MOU	Inositol he	Ppip5k2 Hi	Mus muscu	1129
LRIQ3_MO	Q14DL3	reviewed	LRIQ3_MO	Leucine-ric	Lrriq3 Lrrc	Mus muscu	633
ESPL1_MO	P60330	reviewed	ESPL1_MO	Separin (E	Espl1 Esp1	Mus muscu	2118
SAHH2_M	Q80SW1	reviewed	SAHH2_M	S-adenosy	Ahcyl1 Irbi	Mus muscu	530
EHD1_MO	Q9WVK4	reviewed	EHD1_MO	EH domain	Ehd1 Past1	Mus muscu	534
CK5P2_MC	Q8K389	reviewed	CK5P2_MC	CDK5 regu	Cdk5rap2	Mus muscu	1822
SUN2_MO	Q8BJS4	reviewed	SUN2_MO	SUN doma	Sun2 Unc8	Mus muscu	731
DDX46_M	Q569Z5	reviewed	DDX46_M	Probable A	Ddx46 Kiaa	Mus muscu	1032
FRPD1_MC	A2AKB4	reviewed	FRPD1_MC	FERM and	Frmpd1 Kia	Mus muscu	1549
DEN2A_M	Q8C4S8	reviewed	DEN2A_M	DENN dom	Dennd2a	Mus muscu	1000
UT14B_MC	Q6EJB6	reviewed	UT14B_MC	U3 small n	Utp14b Jsc	Mus muscu	756
EPHA6_M	Q62413	reviewed	EPHA6_M	Ephrin type	Epha6 Ehk	Mus muscu	1035
MAP1A_M	Q9QYR6	reviewed	MAP1A_M	Microtubul	Map1a Mta	Mus muscu	2776
FANCI_MO	Q8K368	reviewed	FANCI_MO	Fanconi an	Fanci	Mus muscu	1330
UBA1_MO	Q02053	reviewed	UBA1_MO	Ubiquitin-l	Uba1 Sbx U	Mus muscu	1058
TCP4_MOL	P11031	reviewed	TCP4_MOL	Activated F	Sub1 Pc4 F	Mus muscu	127
RS5_MOUS	P97461	reviewed	RS5_MOUS	Small ribos	Rps5	Mus muscu	204
DGKH_MO	D3YXJ0	reviewed	DGKH_MO	Diacylglyce	Dgkh	Mus muscu	1211
DEND3_M	A2RT67	reviewed	DEND3_M	DENN dom	Dennd3 Kia	Mus muscu	1274
WDR76_M	A6PWY4	reviewed	WDR76_M	WD repeat	Wdr76	Mus muscu	622
ZCPW1_M	Q6IR42	reviewed	ZCPW1_M	Zinc finger	Zcwpw1 Gr	Mus muscu	630
TTF2_MOU	Q5NC05	reviewed	TTF2_MOU	Transcripti	Ttf2	Mus muscu	1138
KMT5A_MC	Q2YDW7	reviewed	KMT5A_MC	N-lysine m	Kmt5a Set	Mus muscu	349
I12R1_MO	Q60837	reviewed	I12R1_MO	Interleukin	Il12rb1 Il12	Mus muscu	738
GRDN_MO	Q5SNZ0	reviewed	GRDN_MO	Girdin (Akt	Ccdc88a G	Mus muscu	1873
RL15_MOL	Q9CZM2	reviewed	RL15_MOL	Large ribos	Rpl15	Mus muscu	204
MED13_M	Q5SWW4	reviewed	MED13_M	Mediator o	Med13 Kia	Mus muscu	2171
DYH12_MC	Q3V0Q1	reviewed	DYH12_MC	Dynein axo	Dnah12 Dr	Mus muscu	3086
KI16B_MO	B1AVY7	reviewed	KI16B_MO	Kinesin-lik	Kif16b Kiaa	Mus muscu	1312
CING_MOL	P59242	reviewed	CING_MOL	Cingulin	Cgn Kiaa1	Mus muscu	1191
DZIP3_MO	Q7TPV2	reviewed	DZIP3_MO	E3 ubiquiti	Dzip3 Kiaa	Mus muscu	1204
WAPL_MO	Q65Z40	reviewed	WAPL_MO	Wings apa	Wapl Kiaa	Mus muscu	1200
SMCA1_M	Q6PGB8	reviewed	SMCA1_M	Probable g	Smarca1 S	Mus muscu	1046
UN45B_M	Q8CGY6	reviewed	UN45B_M	Protein un	Unc45b Cr	Mus muscu	931
SF3A1_MO	Q8K4Z5	reviewed	SF3A1_MO	Splicing fa	Sf3a1	Mus muscu	791
RAI1_MOU	Q61818	reviewed	RAI1_MOU	Retinoic ac	Rai1 Kiaa1	Mus muscu	1889

Transcripti

From
NCOR2_M
ZFHX4_MC
NFX1_MOU
ZN821_MC
RREB1_MC
MED13_M
BCLF1_MC
ZFHX2_MC
MED1_MO
CDYL2_MC
ZEP1_MOU
GON4L_M
SAP18_MC
HAIR_MOU
PHF2_MOU
MED14_M
MTG16_M
TCP4_MOU
CTCF_MOU
CMTA1_M
ZFAT_MOU

Translation

From
EIF3H_MO

ATP-depen

From
KIF23_MOU
CHD8_MO
DYH12_MC
KI16B_MOU
CLPX_MOU
INO80_MC
HELZ2_MC
ABCA3_MC
MYO5B_M
ABCAC_M
AT1A1_MO
DHX30_M
TTF2_MOU
UBA1_MOU
MCM9_MC
ABCA1_MC
KIF15_MOU
SMCA1_M

US6NL_MC	Q80XC3	reviewed	US6NL_MC	USP6 N-tei	Usp6nl Kia	Mus muscı	819	YTDC2_MC
EIF3H_MOI	Q91WK2	reviewed	EIF3H_MO	Eukaryotic	Eif3h Eif3s	Mus muscı	352	
CLK1_MOU	P22518	reviewed	CLK1_MOU	Dual speci	Clk1 Clk St	Mus muscı	483	
Q6KB66-1	Q6KB66	reviewed	K2C80_HU	Keratin, typ	KRT80 KB2	Homo sapi	452	**p53 pathw**
TULP4_MO	Q9JIL5	reviewed	TULP4_MO	Tubby-rela	Tulp4 Tusp	Mus muscı	1547	From
FLNA_MOU	Q8BTM8	reviewed	FLNA_MOU	Filamin-A (	Flna Fln Flı	Mus muscı	2647	P3C2A_MC
CCD93_MC	Q7TQK5	reviewed	CCD93_MC	Coiled-coil	Ccdc93	Mus muscı	629	APAF_MOU
LIMD2_MO	Q8BGB5	reviewed	LIMD2_MO	LIM domaiı	Limd2	Mus muscı	128	
SCNAA_MC	Q6QIY3	reviewed	SCNAA_MC	Sodium ch	Scn10a Sn	Mus muscı	1958	antioxidant
AMOL1_MC	Q9D4H4	reviewed	AMOL1_MC	Angiomotir	Amotl1	Mus muscı	968	From
Q2KJ83	Q2KJ83	reviewed	CBPN_BOV	Carboxype	CPN1	Bos taurus	462	PRDX3_MC
CLPX_MOU	Q9JHS4	reviewed	CLPX_MOU	ATP-depen	Clpx	Mus muscı	634	
EHBP1_MC	Q69ZW3	reviewed	EHBP1_MC	EH domain	Ehbp1 Kiaa	Mus muscı	1231	oxidative st
DLG5_MOU	E9Q9R9	reviewed	DLG5_MOU	Disks large	Dlg5	Mus muscı	1921	From
EMAL5_MC	Q8BQM8	reviewed	EMAL5_MC	Echinoderı	Eml5	Mus muscı	1977	PA24B_MC
AMPL_MOU	Q9CPY7	reviewed	AMPL_MOU	Cytosol an	Lap3 Lape	Mus muscı	519	
DIDO1_MC	Q8C9B9	reviewed	DIDO1_MC	Death-indı	Dido1 Datf	Mus muscı	2256	ubiquitin-p
PDS5B_MC	Q4VA53	reviewed	PDS5B_MC	Sister chro	Pds5b Apri	Mus muscı	1446	From
CCD66_MC	Q6NS45	reviewed	CCD66_MC	Coiled-coil	Ccdc66	Mus muscı	935	UBA1_MOU
ERLN1_MC	Q91X78	reviewed	ERLN1_MC	Erlin-1 (En	Erlin1 Keo	Mus muscı	348	
VIGLN_MO	Q8VDJ3	reviewed	VIGLN_MO	Vigilin (Hig	Hdlbp	Mus muscı	1268	chaperone
HERC2_MC	Q4U2R1	reviewed	HERC2_MC	E3 ubiquiti	Herc2 Jdf2	Mus muscı	4836	From
5NTC_MOU	Q3V1L4	reviewed	5NTC_MOU	Cytosolic p	Nt5c2	Mus muscı	560	CH10_MOU
RL36A_MO	P83882	reviewed	RL36A_MO	Large ribos	Rpl36a Rpl	Mus muscı	106	TEBP_MOU
RPC2_MOU	P59470	reviewed	RPC2_MOU	DNA-direct	Polr3b	Mus muscı	1133	PPIA_MOU
SLAI1_MOU	Q68FF7	reviewed	SLAI1_MOU	SLAIN moti	Slain1	Mus muscı	579	FKB1A_MC
RHGBA_MC	Q80Y19	reviewed	RHGBA_MC	Rho GTPas	Arhgap11a	Mus muscı	987	
L2GL2_MC	Q3TJ91	reviewed	L2GL2_MC	LLGL scribl	Llgl2 Llglh2	Mus muscı	1027	
DZIP1_MO	Q8BMD2	reviewed	DZIP1_MO	Cilium ass	Dzip1 Kiaa	Mus muscı	852	
ABCA3_MC	Q8R420	reviewed	ABCA3_MC	Phospholip	Abca3	Mus muscı	1704	
BAZ1B_MC	Q9Z277	reviewed	BAZ1B_MC	Tyrosine-pı	Baz1b Wbs	Mus muscı	1479	
DNAI3_MO	B2RY71	reviewed	DNAI3_MO	Dynein axo	Dnai3 Wdr	Mus muscı	923	
H12_MOUS	P15864	reviewed	H12_MOUS	Histone H1	H1-2 H1f2	Mus muscı	212	
TRRAP_MC	Q80YV3	reviewed	TRRAP_MC	Transforma	Trrap	Mus muscı	2565	
CAPS2_MC	Q8BYR5	reviewed	CAPS2_MC	Calcium-d	Cadps2 Ca	Mus muscı	1297	
TEKT5_MO	G5E8A8	reviewed	TEKT5_MO	Tektin-5	Tekt5	Mus muscı	557	
RPA1_MOU	O35134	reviewed	RPA1_MOU	DNA-direct	Polr1a Rpa	Mus muscı	1717	
NFX1_MOU	B1AY10	reviewed	NFX1_MOU	Transcripti	Nfx1	Mus muscı	1114	
SCOT1_MC	Q9D0K2	reviewed	SCOT1_MC	Succinyl-C	Oxct1 Oxct	Mus muscı	520	
CTNA1_MC	P26231	reviewed	CTNA1_MC	Catenin alı	Ctnna1 Ca	Mus muscı	906	
KCNQ2_MC	Q9Z351	reviewed	KCNQ2_MC	Potassium	Kcnq2 Kqt2	Mus muscı	759	
PLVAP_MC	Q91VC4	reviewed	PLVAP_MC	Plasmalen	Plvap Pv1	Mus muscı	438	
XPO2_MOU	Q9ERK4	reviewed	XPO2_MOU	Exportin-2	Cse1l Xpo2	Mus muscı	971	
SPTB2_MO	Q62261	reviewed	SPTB2_MO	Spectrin be	Sptbn1 Elf	Mus muscı	2363	
MYO5B_MC	P21271	reviewed	MYO5B_MC	Unconvent	Myo5b Kiaa	Mus muscı	1818	
LARP1_MC	Q6ZQ58	reviewed	LARP1_MC	La-related	Larp1 Kiaa	Mus muscı	1072	
INT6_MOU	Q6PCM2	reviewed	INT6_MOU	Integrator (	Ints6 Dbi1	Mus muscı	883	
MED14_MC	A2ABV5	reviewed	MED14_MC	Mediator o	Med14 Crs	Mus muscı	1459	

SMKZ_MOU	Q8C0N0	reviewed	SMKZ_MOU	Sperm mot	Gm4922	Mus muscu	497
SPT2_MOL	Q68FG3	reviewed	SPT2_MOL	Protein SPT	Spty2d1	Mus muscu	682
BRWD1_M	Q921C3	reviewed	BRWD1_M	Bromodom	Brwd1 Wdr	Mus muscu	2304
GCN1_MO	E9PVA8	reviewed	GCN1_MO	Stalled ribo	Gcn1 Gcn1	Mus muscu	2671
HELZ2_MC	E9QAM5	reviewed	HELZ2_MC	3'-5' exorib	Helz2	Mus muscu	2947
RN19B_MC	A2A7Q9	reviewed	RN19B_MC	E3 ubiquiti	Rnf19b Ibr	Mus muscu	732
M4K1_MOU	P70218	reviewed	M4K1_MOU	Mitogen-ac	Map4k1 Hp	Mus muscu	827
CMYA5_MC	Q70KF4	reviewed	CMYA5_MC	Cardiomyo	Cmya5 Sr5	Mus muscu	3739
ANR26_MC	Q811D2	reviewed	ANR26_MC	Ankyrin rep	Ankrd26 Ki	Mus muscu	1581
PA24B_MC	P0C871	reviewed	PA24B_MC	Cytosolic p	Pla2g4b	Mus muscu	782
TULP1_MO	Q9Z273	reviewed	TULP1_MO	Tubby-rela	Tulp1	Mus muscu	543
GON4L_MC	Q9DB00	reviewed	GON4L_MC	GON-4-like	Gon4l Gon	Mus muscu	2260
TM87A_MC	Q8BXN9	reviewed	TM87A_MC	Transmem	Tmem87a	Mus muscu	555
PHLP1_MC	Q8CHE4	reviewed	PHLP1_MC	PH domain	Phlpp1 Kia	Mus muscu	1687
DNMT1_MC	P13864	reviewed	DNMT1_MC	DNA (cytos	Dnmt1 Dnr	Mus muscu	1620
NINL_MOU	Q6ZQ12	reviewed	NINL_MOU	Ninein-like	Ninl Kiaa09	Mus muscu	1394
NCAM2_M	O35136	reviewed	NCAM2_M	Neural cell	Ncam2 Oc	Mus muscu	837
DHX30_MC	Q99PU8	reviewed	DHX30_MC	ATP-depen	Dhx30 Hel	Mus muscu	1217
ZN706_MC	Q9D115	reviewed	ZN706_MC	Zinc finger	Znf706 Zfp	Mus muscu	76
JARD2_MO	Q62315	reviewed	JARD2_MO	Protein Jun	Jarid2 Jmj	Mus muscu	1234
TOP2B_MC	Q64511	reviewed	TOP2B_MC	DNA topois	Top2b	Mus muscu	1612
RS6_MOUS	P62754	reviewed	RS6_MOUS	Small ribos	Rps6	Mus muscu	249
PARP1_MC	P11103	reviewed	PARP1_MC	Poly [ADP-r	Parp1 Adpr	Mus muscu	1013
PPIA_MOU	P17742	reviewed	PPIA_MOU	Peptidyl-pr	Ppia	Mus muscu	164
RPGR1_MC	Q9EPQ2	reviewed	RPGR1_MC	X-linked re	Rpgrip1	Mus muscu	1331
RHG35_MC	Q91YM2	reviewed	RHG35_MC	Rho GTPas	Arhgap35 (	Mus muscu	1499
BIG1_MOU	G3X9K3	reviewed	BIG1_MOU	Brefeldin A	Arfgef1	Mus muscu	1846
LRC40_MC	Q9CRC8	reviewed	LRC40_MC	Leucine-ric	Lrrc40	Mus muscu	602
UN45A_MC	Q99KD5	reviewed	UN45A_MC	Protein unc	Unc45a Sn	Mus muscu	944
RBP1_MOU	Q62172	reviewed	RBP1_MOU	RalA-bindi	Ralbp1 Rip	Mus muscu	648
ZN609_MC	Q8BZ47	reviewed	ZN609_MC	Zinc finger	Znf609 Kia	Mus muscu	1413
APC7_MOU	Q9WVM3	reviewed	APC7_MOU	Anaphase-	Anapc7 Ap	Mus muscu	565
CC178_MC	Q8CDV0	reviewed	CC178_MC	Coiled-coil	Ccdc178	Mus muscu	866
LATS1_MO	Q8BYR2	reviewed	LATS1_MO	Serine/thre	Lats1 Wart	Mus muscu	1129
FYV1_MOL	Q9Z1T6	reviewed	FYV1_MOL	1-phospha	Pikfyve Fab	Mus muscu	2097
ZFAT_MOU	Q7TS63	reviewed	ZFAT_MOU	Zinc finger	Zfat Gm92	Mus muscu	1237
PIPNA_MO	P53810	reviewed	PIPNA_MO	Phosphatid	Pitpna Pitp	Mus muscu	271
AF9_MOUS	A2AM29	reviewed	AF9_MOUS	Protein AF-	Mllt3 Af9	Mus muscu	569
SYTL2_MO	Q99N50	reviewed	SYTL2_MO	Synaptotag	Sytl2 Slp2	Mus muscu	950
ZW10_MOU	O54692	reviewed	ZW10_MOU	Centromer	Zw10	Mus muscu	779
RL38_MOL	Q9JJI8	reviewed	RL38_MOL	Large ribos	Rpl38	Mus muscu	70
LC7L2_MO	Q7TNC4	reviewed	LC7L2_MO	Putative RN	Luc7l2	Mus muscu	392
ALPK3_MO	Q924C5	reviewed	ALPK3_MO	Alpha-prot	Alpk3 Kiaa	Mus muscu	1680
UACA_MOU	Q8CGB3	reviewed	UACA_MOU	Uveal auto	Uaca Kiaa1	Mus muscu	1411
ERLN2_MC	Q8BFZ9	reviewed	ERLN2_MC	Erlin-2 (En	Erlin2 Spfh	Mus muscu	340
K0825_MO	Q3UPC7	reviewed	K0825_MO	Uncharact		Mus muscu	1272
SHP1L_MC	Q3TTP0	reviewed	SHP1L_MC	Testicular	Shcbp1l	Mus muscu	639
TNR6C_MC	Q3UHC0	reviewed	TNR6C_MC	Trinucleoti	Tnrc6c Kia	Mus muscu	1690

RESF1_MC	Q5DTW7	reviewed	RESF1_MC	Retroelem	Resf1 Kiaa	Mus muscu	1521
SAFB2_MC	Q80YR5	reviewed	SAFB2_MC	Scaffold at	Safb2	Mus muscu	991
STRBP_MC	Q91WM1	reviewed	STRBP_MC	Spermatid	Strbp Spnr	Mus muscu	672
FAT3_MOU	Q8BNA6	reviewed	FAT3_MOU	Protocadh	Fat3 Gm11	Mus muscu	4555
SCN9A_MC	Q62205	reviewed	SCN9A_MC	Sodium ch	Scn9a Kiaa	Mus muscu	1984
WDR48_M	Q8BH57	reviewed	WDR48_M	WD repeat	Wdr48 Kiaa	Mus muscu	676
VP33B_MC	P59016	reviewed	VP33B_MC	Vacuolar p	Vps33b	Mus muscu	617
BAZ2A_MC	Q91YE5	reviewed	BAZ2A_MC	Bromodom	Baz2a Kiaa	Mus muscu	1889
AKAP1_MC	O08715	reviewed	AKAP1_MC	A-kinase a	Akap1 Akap	Mus muscu	857
ZFHX2_MC	Q2MHN3	reviewed	ZFHX2_MC	Zinc finger	Zfhx2 Kiaa	Mus muscu	2562
CP7B1_MC	Q60991	reviewed	CP7B1_MC	Cytochrom	Cyp7b1	Mus muscu	507
SAM9L_MC	Q69Z37	reviewed	SAM9L_MC	Sterile alph	Samd9l Kia	Mus muscu	1561
CHD8_MO	Q09XV5	reviewed	CHD8_MO	Chromodo	Chd8 Kiaa	Mus muscu	2582
SPTB1_MO	P15508	reviewed	SPTB1_MO	Spectrin be	Sptb Spnb-	Mus muscu	2128
UIF_MOUS	Q91Z49	reviewed	UIF_MOUS	UAP56-inte	Fyttd1 Uif	Mus muscu	317
K2C1B_MC	Q6IFZ6	reviewed	K2C1B_MC	Keratin, typ	Krt77 Krt1b	Mus muscu	572
CCD73_M	Q8CDM4	reviewed	CCD73_M	Coiled-coil	Ccdc73	Mus muscu	1066
SPB1_MOU	Q9DBE9	reviewed	SPB1_MOU	pre-rRNA 2	Ftsj3	Mus muscu	838
RBM27_M	Q5SFM8	reviewed	RBM27_M	RNA-bindir	Rbm27 Kia	Mus muscu	1060
SBP1_MOU	P17563	reviewed	SBP1_MOU	Methaneth	Selenbp1 L	Mus muscu	472
AFF3_MOU	P51827	reviewed	AFF3_MOU	AF4/FMR2	Aff3 Laf4	Mus muscu	1254
MSH3_MO	P13705	reviewed	MSH3_MO	DNA mism	Msh3 Rep-	Mus muscu	1091
DEK_MOUS	Q7TNV0	reviewed	DEK_MOUS	Protein DE	Dek	Mus muscu	380
TCOF_MOU	O08784	reviewed	TCOF_MOU	Treacle prc	Tcof1	Mus muscu	1320
KANL3_MC	A2RSY1	reviewed	KANL3_MC	KAT8 regul	Kansl3 Kia	Mus muscu	903
F10A1_MO	Q99L47	reviewed	F10A1_MO	Hsc70-inte	St13 Fam1	Mus muscu	371
BPNT1_MC	Q9Z0S1	reviewed	BPNT1_MC	3'(2'),5'-bis	Bpnt1	Mus muscu	308
RL40_MOU	P62984	reviewed	RL40_MOU	Ubiquitin-r	Uba52 Ubc	Mus muscu	128
PSPC1_MC	Q8R326	reviewed	PSPC1_MC	Paraspeck	Pspc1 Psp	Mus muscu	523
2A5G_MOU	Q60996	reviewed	2A5G_MOU	Serine/thre	Ppp2r5c	Mus muscu	524
CCD13_M	D3YV10	reviewed	CCD13_M	Coiled-coil	Ccdc13	Mus muscu	709
NDUA7_M	Q9Z1P6	reviewed	NDUA7_M	NADH deh	Ndufa7	Mus muscu	113
JKIP3_MOU	Q5DTN8	reviewed	JKIP3_MOU	Janus kina	Jakmip3 Ki	Mus muscu	844
COPA_MO	Q8CIE6	reviewed	COPA_MO	Coatomer	Copa	Mus muscu	1224
CTCF_MOU	Q61164	reviewed	CTCF_MOU	Transcripti	Ctcf	Mus muscu	736
TET3_MOU	Q8BG87	reviewed	TET3_MOU	Methylcyto	Tet3	Mus muscu	1803
DYRK4_MC	Q8BI55	reviewed	DYRK4_MC	Dual speci	Dyrk4	Mus muscu	632
LRP1B_MC	Q9JI18	reviewed	LRP1B_MC	Low-densit	Lrp1b Lrpd	Mus muscu	4599
GA2L3_MC	Q3UWW6	reviewed	GA2L3_MC	GAS2-like	Gas2l3	Mus muscu	683
GEMI5_MC	Q8BX17	reviewed	GEMI5_MC	Gem-asso	Gemin5	Mus muscu	1502
CNTRL_MC	A2AL36	reviewed	CNTRL_MC	Centriolin	Cntrl Cep1	Mus muscu	2334
SUCO_MO	Q8C341	reviewed	SUCO_MO	SUN doma	Suco Opt	Mus muscu	1250
GANP_MO	Q9WUU9	reviewed	GANP_MO	Germinal-c	Mcm3ap G	Mus muscu	1971
CC186_MC	Q8C9S4	reviewed	CC186_MC	Coiled-coil	Ccdc186 C	Mus muscu	917
CSN4_MO	O88544	reviewed	CSN4_MO	COP9 sign	Cops4 Csn	Mus muscu	406
S22AM_MC	Q8R0S9	reviewed	S22AM_MC	Solute carr	Slc22a22 C	Mus muscu	554
NBEA_MOU	Q9EPN1	reviewed	NBEA_MOU	Neurobeac	Nbea Lyst2	Mus muscu	2936
SAP18_MC	O55128	reviewed	SAP18_MC	Histone de	Sap18	Mus muscu	153

CO6A1_MO	Q04857	reviewed	CO6A1_MO	Collagen a	Col6a1	Mus muscu	1025
SAPC2_MO	Q9D818	reviewed	SAPC2_MO	Suppresso	Sapcd2	Mus muscu	391
PCM1_MO	Q9R0L6	reviewed	PCM1_MO	Pericentrio	Pcm1	Mus muscu	2025
BCDO1_M	Q9JJS6	reviewed	BCDO1_M	Beta,beta-	Bco1 Bcdo	Mus muscu	566
CPIN1_MO	Q8WTY4	reviewed	CPIN1_MO	Anamorsin	Ciapin1	Mus muscu	309
NSE3_MOU	Q9CPR8	reviewed	NSE3_MOU	Non-struct	Nsmce3 M	Mus muscu	279
NSD3_MOU	Q6P2L6	reviewed	NSD3_MOU	Histone-lys	Nsd3 Whsc	Mus muscu	1439
FANCM_M	Q8BGE5	reviewed	FANCM_M	Fanconi an	Fancm Kia	Mus muscu	2021
DEN4B_MO	Q3U1Y4	reviewed	DEN4B_MO	DENN dom	Dennd4b E	Mus muscu	1499
IQGA2_MO	Q3UQ44	reviewed	IQGA2_MO	Ras GTPas	Iqgap2	Mus muscu	1575
TBCD1_MO	Q60949	reviewed	TBCD1_MO	TBC1 doma	Tbc1d1 Kia	Mus muscu	1255
RBP2_MOU	Q9ERU9	reviewed	RBP2_MOU	E3 SUMO-p	Ranbp2	Mus muscu	3053
KIF23_MOU	E9Q5G3	reviewed	KIF23_MOU	Kinesin-like	Kif23	Mus muscu	953
FETUA_MO	P29699	reviewed	FETUA_MO	Alpha-2-HS	Ahsg Fetua	Mus muscu	345
CO4B_MOU	P01029	reviewed	CO4B_MOU	Compleme	C4b C4	Mus muscu	1738
CFA45_MO	Q9D9U9	reviewed	CFA45_MO	Cilia- and f	Cfap45 Cc	Mus muscu	551
INO80_MO	Q6ZPV2	reviewed	INO80_MO	Chromatin	Ino80 Inoc	Mus muscu	1559
VIP1_MOU	A2ARP1	reviewed	VIP1_MOU	Inositol he	Ppip5k1 Hi	Mus muscu	1436
IF140_MOU	E9PY46	reviewed	IF140_MOU	Intraflagell	Ift140 WD1	Mus muscu	1464
SNUT1_MO	Q9Z315	reviewed	SNUT1_MO	U4/U6.U5 t	Sart1 Haf	Mus muscu	806
APAF_MOU	O88879	reviewed	APAF_MOU	Apoptotic p	Apaf1	Mus muscu	1249
PLCB1_MO	Q9Z1B3	reviewed	PLCB1_MO	1-phospha	Plcb1 Plcb	Mus muscu	1216
PDE6A_MO	P27664	reviewed	PDE6A_MO	Rod cGMP-	Pde6a Mpa	Mus muscu	859
RYR1_MOU	E9PZQ0	reviewed	RYR1_MOU	Ryanodine	Ryr1	Mus muscu	5035
ZEP1_MOU	Q03172	reviewed	ZEP1_MOU	Zinc finger	Hivep1 Cry	Mus muscu	2688
EP15R_MO	Q60902	reviewed	EP15R_MO	Epidermal	Eps15l1 Ep	Mus muscu	907
ERCC6_MO	F8VPZ5	reviewed	ERCC6_MO	DNA excisi	Ercc6 Csb	Mus muscu	1481
FUMH_MO	P97807	reviewed	FUMH_MO	Fumarate h	Fh Fh1	Mus muscu	507
WASC4_M	Q3UMB9	reviewed	WASC4_M	WASH com	Washc4 Ki	Mus muscu	1173
TLL1_MOU	Q62381	reviewed	TLL1_MOU	Tolloid-like	Tll1 Tll	Mus muscu	1013
APCL_MOU	Q9Z1K7	reviewed	APCL_MOU	Adenomato	Apc2	Mus muscu	2274
CO6A2_MO	Q02788	reviewed	CO6A2_MO	Collagen a	Col6a2	Mus muscu	1034
IGFN1_MO	Q3KNY0	reviewed	IGFN1_MO	Immunoglo	Igfn1	Mus muscu	2849
TSP2_MOU	Q03350	reviewed	TSP2_MOU	Thrombosp	Thbs2 Tsp2	Mus muscu	1172
TEX10_MO	Q3URQ0	reviewed	TEX10_MO	Testis-expr	Tex10	Mus muscu	928
ARI4A_MO	F8VPQ2	reviewed	ARI4A_MO	AT-rich inte	Arid4a Rbb	Mus muscu	1261
FLIP1_MOU	Q9CS72	reviewed	FLIP1_MOU	Filamin-A-i	Filip1	Mus muscu	1214
PLSI_MOUS	Q3V0K9	reviewed	PLSI_MOUS	Plastin-1	Pls1	Mus muscu	630
HD_MOUS	P42859	reviewed	HD_MOUS	Huntingtin	Htt Hd Hdh	Mus muscu	3119
CMTA1_MO	A2A891	reviewed	CMTA1_MO	Calmodulin	Camta1 Kia	Mus muscu	1682
PAR3L_MO	Q9CSB4	reviewed	PAR3L_MO	Partitioning	Pard3b Als	Mus muscu	1203
BCAS3_MO	Q8CCN5	reviewed	BCAS3_MO	BCAS3 mic	Bcas3	Mus muscu	928
RADI_MOU	P26043	reviewed	RADI_MOU	Radixin (ES	Rdx	Mus muscu	583
ABCAC_MO	E9Q876	reviewed	ABCAC_MO	Glucosylce	Abca12	Mus muscu	2595
PMFBP_MO	Q9WVQ0	reviewed	PMFBP_MO	Polyamine-	Pmfbp1 Sta	Mus muscu	1022
DLGP5_MO	Q8K4R9	reviewed	DLGP5_MO	Disks large	Dlgap5 Dlg	Mus muscu	808
OTU7B_MO	B2RUR8	reviewed	OTU7B_MO	OTU doma	Otud7b	Mus muscu	840
K1143_MO	Q8K039	reviewed	K1143_MO	Uncharact		Mus muscu	155

MARF1_MO	Q8BJ34	reviewed	MARF1_MO	Meiosis reg	Marf1 Kiaa	Mus muscu	1730
NALCN_MO	Q8BXR5	reviewed	NALCN_MO	Sodium lea	Nalcn Vgcn	Mus muscu	1738
MA7D2_MO	A2AG50	reviewed	MA7D2_MO	MAP7 dom	Map7d2 M	Mus muscu	781
TEX11_MO	Q14AT2	reviewed	TEX11_MO	Testis-expr	Tex11 Zip4	Mus muscu	947
ZFHX4_MC	Q9JJN2	reviewed	ZFHX4_MC	Zinc finger	Zfhx4 Zfh4	Mus muscu	3550
CRCC2_MO	F6XLV1	reviewed	CRCC2_MO	Ciliary root	Crocc2	Mus muscu	1638
NWD1_MC	A6H603	reviewed	NWD1_MC	NACHT dor	Nwd1	Mus muscu	1563
PRDX3_MC	P20108	reviewed	PRDX3_MC	Thioredoxi	Prdx3 Aop1	Mus muscu	257
PHF2_MOU	Q9WTU0	reviewed	PHF2_MOU	Lysine-spe	Phf2 Kiaa0	Mus muscu	1096
AN34B_MC	Q3UUF8	reviewed	AN34B_MC	Ankyrin rep	Ankrd34b I	Mus muscu	508
UNC5C_M	O08747	reviewed	UNC5C_M	Netrin rece	Unc5c Rcm	Mus muscu	931
CH10_MOI	Q64433	reviewed	CH10_MOI	10 kDa hea	Hspe1	Mus muscu	102
HNRPL_MC	Q8R081	reviewed	HNRPL_MC	Heterogen	Hnrnpl Hnr	Mus muscu	586
TEFM_MOU	Q5SSK3	reviewed	TEFM_MOU	Transcripti	Tefm	Mus muscu	364
NAA16_MC	Q9DBB4	reviewed	NAA16_MC	N-alpha-ac	Naa16 Nar	Mus muscu	864
YTDC2_MC	B2RR83	reviewed	YTDC2_MC	3'-5' RNA h	Ythdc2	Mus muscu	1445
TALD3_MO	E9PV87	reviewed	TALD3_MO	Protein TAL	Talpid3	Mus muscu	1520
EME1_MOU	Q8BJW7	reviewed	EME1_MOU	Crossover	Eme1	Mus muscu	570
ANLN_MOU	Q8K298	reviewed	ANLN_MOU	Anillin	Anln	Mus muscu	1121
SPTA1_MO	P08032	reviewed	SPTA1_MO	Spectrin al	Spta1 Spna	Mus muscu	2415
SVIL_MOUS	Q8K4L3	reviewed	SVIL_MOUS	Supervillin	Svil	Mus muscu	2170
NUB1_MOI	P54729	reviewed	NUB1_MOI	NEDD8 ulti	Nub1 Nyre	Mus muscu	614
TOPB1_MC	Q6ZQF0	reviewed	TOPB1_MC	DNA topois	Topbp1 Kia	Mus muscu	1515
CC187_MC	Q8C5V8	reviewed	CC187_MC	Coiled-coil	Ccdc187	Mus muscu	958
UBP8_MOU	Q80U87	reviewed	UBP8_MOU	Ubiquitin c	Usp8 Kiaa0	Mus muscu	1080
CFA54_MC	Q8C6S9	reviewed	CFA54_MC	Cilia- and f	Cfap54	Mus muscu	3106
NOG2_MO	Q99LH1	reviewed	NOG2_MO	Nucleolar (	Gnl2	Mus muscu	728
BCLF1_MC	Q8K019	reviewed	BCLF1_MC	Bcl-2-asso	Bclaf1 Btf I	Mus muscu	919
ZN318_MC	Q99PP2	reviewed	ZN318_MC	Zinc finger	Znf318 Tzf	Mus muscu	2237
PRP4B_MC	Q61136	reviewed	PRP4B_MC	Serine/thre	Prpf4b Cbp	Mus muscu	1007
SC11A_MC	Q9R0P6	reviewed	SC11A_MC	Signal pept	Sec11a Se	Mus muscu	179
BGH3_MOI	P82198	reviewed	BGH3_MOI	Transformi	Tgfbi	Mus muscu	683
TGFB3_MC	P17125	reviewed	TGFB3_MC	Transformi	Tgfb3	Mus muscu	410
FREM1_MC	Q684R7	reviewed	FREM1_MC	FRAS1-rela	Frem1	Mus muscu	2191
CPSF2_MC	O35218	reviewed	CPSF2_MC	Cleavage a	Cpsf2 Cps	Mus muscu	782
PROF1_MC	P62962	reviewed	PROF1_MC	Profilin-1 (I	Pfn1	Mus muscu	140
VP13A_MC	Q5H8C4	reviewed	VP13A_MC	Intermemb	Vps13a Ch	Mus muscu	3166
TERB1_MO	Q8C0V1	reviewed	TERB1_MO	Telomere r	Terb1 Ccdc	Mus muscu	768
CO4A3_MC	Q9QZS0	reviewed	CO4A3_MC	Collagen a	Col4a3	Mus muscu	1669
ADCY9_MC	P51830	reviewed	ADCY9_MC	Adenylate	Adcy9	Mus muscu	1353
GSTP1_MC	P19157	reviewed	GSTP1_MC	Glutathion	Gstp1 Gstp	Mus muscu	210
PHIPL_MO	Q8BGT8	reviewed	PHIPL_MO	Phytanoyl-	Phyhipl	Mus muscu	375
CIP2A_MO	Q8BWY9	reviewed	CIP2A_MO	Protein CIF	Cip2a Kiaa	Mus muscu	907
IL18R_MOI	Q61098	reviewed	IL18R_MOI	Interleukin	Il18r1	Mus muscu	537
CCD80_MC	Q8R2G6	reviewed	CCD80_MC	Coiled-coil	Ccdc80 Ur	Mus muscu	949
DBIL5_MO	O09035	reviewed	DBIL5_MO	Diazepam-	Dbil5	Mus muscu	87
LCA5_MOU	Q80ST9	reviewed	LCA5_MOU	Lebercilin (	Lca5	Mus muscu	704
TMTC3_MC	Q8BRH0	reviewed	TMTC3_MC	Protein O-r	Tmtc3	Mus muscu	920

RWDD1_M	Q9CQK7	reviewed	RWDD1_M	RWD doma	Rwdd1 Dfr	Mus muscu	243
PAXB1_MC	P58501	reviewed	PAXB1_MC	PAX3- and	Paxbp1 Gc	Mus muscu	919
VCIP1_MO	Q8CDG3	reviewed	VCIP1_MO	Deubiquiti	Vcpip1 Vci	Mus muscu	1220
PA1B2_MC	Q61206	reviewed	PA1B2_MC	Platelet-ac	Pafah1b2 P	Mus muscu	229
SZT2_MOU	A2A9C3	reviewed	SZT2_MOU	KICSTOR c	Szt2	Mus muscu	3431
ERC6L_MC	Q8BHK9	reviewed	ERC6L_MC	DNA excisi	Ercc6l	Mus muscu	1240
MED1_MO	Q925J9	reviewed	MED1_MO	Mediator o	Med1 Crsp	Mus muscu	1575
PBX2_MOU	O35984	reviewed	PBX2_MOU	Pre-B-cell l	Pbx2	Mus muscu	430
LRBA_MOU	Q9ESE1	reviewed	LRBA_MOU	Lipopolysa	Lrba Bgl Lb	Mus muscu	2856
THIKA_MO	Q921H8	reviewed	THIKA_MO	3-ketoacyl-	Acaa1a Ac	Mus muscu	424
LKHA4_MC	P24527	reviewed	LKHA4_MC	Leukotrien	Lta4h	Mus muscu	611
FAN1_MOU	Q69ZT1	reviewed	FAN1_MOU	Fanconi-as	Fan1 Kiaa1	Mus muscu	1020
NAV3_MOU	Q80TN7	reviewed	NAV3_MOU	Neuron na	Nav3 Kiaa(	Mus muscu	2359
WFS1_MO	P56695	reviewed	WFS1_MO	Wolframin	Wfs1	Mus muscu	890
CO6A6_M(	Q8C6K9	reviewed	CO6A6_M(	Collagen a	Col6a6	Mus muscu	2265
ANR31_MC	A0A140LI8	reviewed	ANR31_MC	Ankyrin rep	Ankrd31	Mus muscu	1857
PDIP3_MO	Q8BG81	reviewed	PDIP3_MO	Polymeras	Poldip3	Mus muscu	420
TSP50_MO	Q8BLH5	reviewed	TSP50_MO	Probable tl	Prss50 Tsp	Mus muscu	439
NMUR1_M	O55040	reviewed	NMUR1_M	Neuromed	Nmur1 Gpi	Mus muscu	428
REXO4_MC	Q6PAQ4	reviewed	REXO4_MC	RNA exonu	Rexo4 Gm:	Mus muscu	432
ITPR1_MOU	P11881	reviewed	ITPR1_MOU	Inositol 1,4	Itpr1 Insp3	Mus muscu	2749
CIRBP_MO	P60824	reviewed	CIRBP_MO	Cold-induc	Cirbp Cirp	Mus muscu	172
GAS2_MOU	P11862	reviewed	GAS2_MOU	Growth arr	Gas2 Gas-:	Mus muscu	314
CDC5L_M(	Q6A068	reviewed	CDC5L_M(	Cell divisio	Cdc5l Kiaa	Mus muscu	802
GCC2_MO	Q8CHG3	reviewed	GCC2_MO	GRIP and c	Gcc2 Kiaa(	Mus muscu	1680
UNC80_M(	Q8BLN6	reviewed	UNC80_M(	Protein unc	Unc80 Kiaa	Mus muscu	3326
NEDD1_M(	P33215	reviewed	NEDD1_M(	Protein NE	Nedd1 Nec	Mus muscu	660
ANM9_MO	Q3U3W5	reviewed	ANM9_MO	Protein arg	Prmt9 Prm	Mus muscu	846
MORC3_M	F7BJB9	reviewed	MORC3_M	MORC fam	Morc3 Nxp	Mus muscu	942
HRH1_MO	P70174	reviewed	HRH1_MO	Histamine	Hrh1 Bphs	Mus muscu	488
CE170_MC	Q6A065	reviewed	CE170_MC	Centrosom	Cep170 Kia	Mus muscu	1588
ZN521_MC	Q6KAS7	reviewed	ZN521_MC	Zinc finger	Znf521 Evi:	Mus muscu	1311
TUT7_MOU	Q5BLK4	reviewed	TUT7_MOU	Terminal u	Tut7 Kiaa1	Mus muscu	1491
FAT4_MOU	Q2PZL6	reviewed	FAT4_MOU	Protocadh	Fat4 Fatj	Mus muscu	4981
PREX1_MC	Q69ZK0	reviewed	PREX1_MC	Phosphatic	Prex1 Kiaa	Mus muscu	1650
TARA_MOU	Q99KW3	reviewed	TARA_MOU	TRIO and F	Triobp Kiaa	Mus muscu	2014
KAPCA_MC	P05132	reviewed	KAPCA_MC	cAMP-depe	Prkaca Pka	Mus muscu	351
H2AW_MO	Q8CCK0	reviewed	H2AW_MO	Core histo	Macroh2a2	Mus muscu	372
KCNH5_M(	Q920E3	reviewed	KCNH5_M(	Potassium	Kcnh5 Eag:	Mus muscu	988
DPOLQ_M(	Q8CGS6	reviewed	DPOLQ_M(	DNA polym	Polq Chao:	Mus muscu	2544
MYOME_M	Q80YT7	reviewed	MYOME_M	Myomegali	Pde4dip Ki	Mus muscu	2224
CDYL2_MC	Q9D5D8	reviewed	CDYL2_MC	Chromodo	Cdyl2	Mus muscu	503
FYCO1_M(	Q8VDC1	reviewed	FYCO1_M(	FYVE and c	Fyco1	Mus muscu	1437
RBM28_M(	Q8CGC6	reviewed	RBM28_M(	RNA-bindir	Rbm28	Mus muscu	750
MCM9_MC	Q2KHI9	reviewed	MCM9_MC	DNA helica	Mcm9 Mcn	Mus muscu	1134
CO4A4_M(	Q9QZR9	reviewed	CO4A4_M(	Collagen a	Col4a4	Mus muscu	1682
TEBP_MOU	Q9R0Q7	reviewed	TEBP_MOU	Prostaglan	Ptges3 Sid:	Mus muscu	160
ADT1_MOU	P48962	reviewed	ADT1_MOU	ADP/ATP tr	Slc25a4 Aa	Mus muscu	298

RED2_MOU	Q9JI20	reviewed	RED2_MOU	Double-str	Adarb2 Ada	Mus muscu	745
FKB1A_MC	P26883	reviewed	FKB1A_MC	Peptidyl-pr	Fkbp1a Fkb	Mus muscu	108
TT21A_MO	Q8C0S4	reviewed	TT21A_MO	Tetratricop	Ttc21a Thn	Mus muscu	1314
CDK13_MC	Q69ZA1	reviewed	CDK13_MC	Cyclin-dep	Cdk13 Cdc	Mus muscu	1511
RASD1_MC	O35626	reviewed	RASD1_MC	Dexametha	Rasd1 Dex	Mus muscu	280
SGO2_MOU	Q7TSY8	reviewed	SGO2_MOU	Shugoshin	Sgo2 Sgol2	Mus muscu	1164
IDHP_MOU	P54071	reviewed	IDHP_MOU	Isocitrate c	Idh2	Mus muscu	452
TSR1_MOU	Q5SWD9	reviewed	TSR1_MOU	Pre-rRNA-p	Tsr1 Kiaa14	Mus muscu	803
CCD71_MC	Q8VEG0	reviewed	CCD71_MC	Coiled-coil	Ccdc71	Mus muscu	433
CISD1_MO	Q91WS0	reviewed	CISD1_MO	CDGSH iro	Cisd1 D10	Mus muscu	108
E41L2_MO	O70318	reviewed	E41L2_MO	Band 4.1-li	Epb41l2 Ep	Mus muscu	988
SURF6_MC	P70279	reviewed	SURF6_MC	Surfeit locu	Surf6 Surf-	Mus muscu	355
RHG20_MC	Q6IFT4	reviewed	RHG20_MC	Rho GTPas	Arhgap20 K	Mus muscu	1182
ACOT9_MC	Q9R0X4	reviewed	ACOT9_MC	Acyl-coenz	Acot9 Acat	Mus muscu	439
PHF23_MC	Q8BSN5	reviewed	PHF23_MC	PHD finger	Phf23	Mus muscu	401
MAST4_MC	Q811L6	reviewed	MAST4_MC	Microtubul	Mast4	Mus muscu	2618
PACS1_MC	Q8K212	reviewed	PACS1_MC	Phosphofu	Pacs1	Mus muscu	961
P3C2A_MC	Q61194	reviewed	P3C2A_MC	Phosphatic	Pik3c2a Cp	Mus muscu	1686
PHB1_MOU	P67778	reviewed	PHB1_MOU	Prohibitin 1	Phb1 Phb	Mus muscu	272
SBNO1_MC	Q689Z5	reviewed	SBNO1_MC	Protein stra	Sbno1 Sno	Mus muscu	1390
3HIDH_MC	Q99L13	reviewed	3HIDH_MC	3-hydroxyis	Hibadh	Mus muscu	335
MPP8_MOU	Q3TYA6	reviewed	MPP8_MOU	M-phase pl	Mphosph8	Mus muscu	858
NCOR2_MC	Q9WU42	reviewed	NCOR2_MC	Nuclear re	Ncor2 Smr	Mus muscu	2472
LRP2_MOU	A2ARV4	reviewed	LRP2_MOU	Low-densit	Lrp2	Mus muscu	4660
MA2A1_MC	P27046	reviewed	MA2A1_MC	Alpha-man	Man2a1 Ma	Mus muscu	1150
DLGP1_MC	Q9D415	reviewed	DLGP1_MC	Disks large	Dlgap1 Gka	Mus muscu	992
TCEA3_MC	P23881	reviewed	TCEA3_MC	Transcripti	Tcea3 Tfiis	Mus muscu	347
ABCA1_MC	P41233	reviewed	ABCA1_MC	Phospholip	Abca1 Abc	Mus muscu	2261
GNPAT_MC	P98192	reviewed	GNPAT_MC	Dihydroxya	Gnpat Dha	Mus muscu	678
USH1C_MC	Q9ES64	reviewed	USH1C_MC	Harmonin	Ush1c	Mus muscu	910
UN13A_MC	Q4KUS2	reviewed	UN13A_MC	Protein unc	Unc13a	Mus muscu	1712
FNBP1_MC	Q80TY0	reviewed	FNBP1_MC	Formin-bin	Fnbp1 Fbp	Mus muscu	616
RL8_MOUS	P62918	reviewed	RL8_MOUS	Large ribos	Rpl8	Mus muscu	257
1433G_MC	P61982	reviewed	1433G_MC	14-3-3 prot	Ywhag	Mus muscu	247
NKTR_MOU	P30415	reviewed	NKTR_MOU	NK-tumor r	Nktr	Mus muscu	1453
STAR9_MO	Q80TF6	reviewed	STAR9_MO	StAR-relate	Stard9 Kiaa	Mus muscu	4561
ZN821_MC	Q6PD05	reviewed	ZN821_MC	Zinc finger	Znf821 Zfp	Mus muscu	413
WDR1_MO	O88342	reviewed	WDR1_MO	WD repeat	Wdr1	Mus muscu	606
FSTL5_MO	Q8BFR2	reviewed	FSTL5_MO	Follistatin-	Fstl5 Kiaa1	Mus muscu	847
TADBP_MC	Q921F2	reviewed	TADBP_MC	TAR DNA-b	Tardbp Tdp	Mus muscu	414
HAIR_MOU	Q61645	reviewed	HAIR_MOU	Lysine-spe	Hr	Mus muscu	1182
PYR1_MOU	B2RQC6	reviewed	PYR1_MOU	Multifuncti	Cad	Mus muscu	2225
SLF2_MOU	Q6P9P0	reviewed	SLF2_MOU	SMC5-SMC	Slf2 Fam17	Mus muscu	1278
ITPR2_MOU	Q9Z329	reviewed	ITPR2_MOU	Inositol 1,4	Itpr2 Itpr5	Mus muscu	2701
ARHG4_MC	Q7TNR9	reviewed	ARHG4_MC	Rho guanir	Arhgef4 Kia	Mus muscu	484
TGO1_MOU	Q8BI84	reviewed	TGO1_MOU	Transport a	Mia3 Kiaa0	Mus muscu	1930
CSF3_MOU	P09920	reviewed	CSF3_MOU	Granulocyt	Csf3 Csfg	Mus muscu	208
OXR1_MOU	Q4KMM3	reviewed	OXR1_MOU	Oxidation r	Oxr1 C7 Gr	Mus muscu	866

MRCKA_M(	Q3UU96	reviewed	MRCKA_M(	Serine/thre	Cdc42bpa	Mus muscı	1719
PHRF1_MC	A6H619	reviewed	PHRF1_MC	PHD and R	Phrf1 Kiaa:	Mus muscı	1682
VWA3A_M(	Q3UVV9	reviewed	VWA3A_M(	von Willebı	Vwa3a	Mus muscı	1148
PPR26_MC	Q6A025	reviewed	PPR26_MC	Protein pho	Ppp1r26 G	Mus muscı	1163
KCNH3_M(	Q9WVJ0	reviewed	KCNH3_M(	Potassium	Kcnh3 Elk2	Mus muscı	1095
ITSN2_MOI	Q9Z0R6	reviewed	ITSN2_MOI	Intersectin	Itsn2 Ese2	Mus muscı	1659
PTN14_MC	Q62130	reviewed	PTN14_MC	Tyrosine-pı	Ptpn14	Mus muscı	1189
RREB1_MC	Q3UH06	reviewed	RREB1_MC	Ras-respor	Rreb1	Mus muscı	1700
SF3B1_MC	Q99NB9	reviewed	SF3B1_MC	Splicing fa	Sf3b1 Sap:	Mus muscı	1304
H2AV_MOI	Q3THW5	reviewed	H2AV_MOI	Histone H2	H2az2 H2a	Mus muscı	128
AFF1_MOL	O88573	reviewed	AFF1_MOL	AF4/FMR2	Aff1 Mllt2 I	Mus muscı	1216
SASH1_MC	P59808	reviewed	SASH1_MC	SAM and S	Sash1	Mus muscı	1230
SHRM2_M(	A2ALU4	reviewed	SHRM2_M(	Protein Shı	Shroom2 A	Mus muscı	1481
KIF15_MOI	Q6P9L6	reviewed	KIF15_MOI	Kinesin-lik	Kif15 Klp2	Mus muscı	1387
CEFIP_MO	D3Z1D3	reviewed	CEFIP_MO	Cardiac-er	CEFIP	Mus muscı	1412
FIL1L_MOI	Q6P6L0	reviewed	FIL1L_MOI	Filamin A-i	Filip1l Doc	Mus muscı	1131
RIMS2_MO	Q9EQZ7	reviewed	RIMS2_MO	Regulating	Rims2 Rab	Mus muscı	1530
ROAA_MOI	Q99020	reviewed	ROAA_MOI	Heterogen	Hnrnpab C	Mus muscı	285
SPAG1_MC	Q80ZX8	reviewed	SPAG1_MC	Sperm-ass	Spag1 Tpis	Mus muscı	901
IGS10_MO	Q3V1M1	reviewed	IGS10_MO	Immunoglo	Igsf10	Mus muscı	2594
PSA3_MOL	O70435	reviewed	PSA3_MOL	Proteasom	Psma3	Mus muscı	255
ESF1_MOL	Q3V1V3	reviewed	ESF1_MOL	ESF1 homo	Esf1 Abtap	Mus muscı	845
MAP7_MOI	O88735	reviewed	MAP7_MOI	Ensconsin	Map7 Mtap	Mus muscı	730
SDCG8_M(	Q80UF4	reviewed	SDCG8_M(	Serologica	Sdccag8 C	Mus muscı	717
NCKX1_M(	Q91WD8	reviewed	NCKX1_M(	Sodium/pc	Slc24a1 N	Mus muscı	1130
PTPA_MOL	P58389	reviewed	PTPA_MOL	Serine/thre	Ptpa Ppp2ı	Mus muscı	323
CETN4_MC	Q8K4K1	reviewed	CETN4_MC	Centrin-4 (	Cetn4 Cen	Mus muscı	168
DACT2_MC	Q7TN08	reviewed	DACT2_MC	Dapper hoı	Dact2 Dpr:	Mus muscı	757
N6MT1_M(	Q6SKR2	reviewed	N6MT1_M(	Methyltran	N6amt1 H	Mus muscı	214
REV1_MOL	Q920Q2	reviewed	REV1_MOL	DNA repair	Rev1 Rev1l	Mus muscı	1249
CE112_MC	Q5PR68	reviewed	CE112_MC	Centrosom	Cep112 Cc	Mus muscı	954
CLAP1_MC	Q80TV8	reviewed	CLAP1_MC	CLIP-asso	Clasp1 Kia	Mus muscı	1535
ASXL3_MO	Q8C4A5	reviewed	ASXL3_MO	Putative Pc	Asxl3	Mus muscı	2259
SHIP1_MO	Q9ES52	reviewed	SHIP1_MO	Phosphatic	Inpp5d 7a3	Mus muscı	1191
NUSAP_M(	Q9ERH4	reviewed	NUSAP_M(	Nucleolar a	Nusap1	Mus muscı	427
RGPA1_MC	Q6GYP7	reviewed	RGPA1_MC	Ral GTPase	Ralgapa1 (	Mus muscı	2035
CSN1_MOI	Q99LD4	reviewed	CSN1_MOI	COP9 signa	Gps1 Cops	Mus muscı	471

ion regulator activity (GO: 0140110)

Entry	Reviewed	Entry Nam	Protein nar	Gene Nam	Organism	Mass
Q9WU42	reviewed	NCOR2_M	Nuclear re	Ncor2 Smr	Mus muscı	269807
Q9JJN2	reviewed	ZFHX4_MC	Zinc finger	Zfhx4 Zfh4	Mus muscı	392323
B1AY10	reviewed	NFX1_MOL	Transcripti	Nfx1	Mus muscı	123811
Q6PD05	reviewed	ZN821_MC	Zinc finger	Znf821 Zfp	Mus muscı	46828
Q3UH06	reviewed	RREB1_MC	Ras-respor	Rreb1	Mus muscı	184154
Q5SWW4	reviewed	MED13_M(	Mediator o	Med13 Kia	Mus muscı	238591
Q8K019	reviewed	BCLF1_MC	Bcl-2-asso	Bclaf1 Btf I	Mus muscı	106002
Q2MHN3	reviewed	ZFHX2_MC	Zinc finger	Zfhx2 Kiaa:	Mus muscı	273485
Q925J9	reviewed	MED1_MOI	Mediator o	Med1 Crsp	Mus muscı	167141
Q9D5D8	reviewed	CDYL2_MC	Chromodo	Cdyl2	Mus muscı	56142
Q03172	reviewed	ZEP1_MOL	Zinc finger	Hivep1 Cry	Mus muscı	288344
Q9DB00	reviewed	GON4L_M(	GON-4-like	Gon4l Gon	Mus muscı	248742
O55128	reviewed	SAP18_MC	Histone de	Sap18	Mus muscı	17595
Q61645	reviewed	HAIR_MOU	Lysine-spe	Hr	Mus muscı	127193
Q9WTU0	reviewed	PHF2_MOL	Lysine-spe	Phf2 Kiaa0	Mus muscı	120814
A2ABV5	reviewed	MED14_M(	Mediator o	Med14 Crs	Mus muscı	160966
O54972	reviewed	MTG16_M(	Protein CB	Cbfa2t3 Cl	Mus muscı	68032
P11031	reviewed	TCP4_MOL	Activated F	Sub1 Pc4 F	Mus muscı	14427
Q61164	reviewed	CTCF_MOL	Transcripti	Ctcf	Mus muscı	83745
A2A891	reviewed	CMTA1_M(	Calmoduli	Camta1 Kia	Mus muscı	184319
Q7TS63	reviewed	ZFAT_MOU	Zinc finger	Zfat Gm92	Mus muscı	137883

n regulator activity (GO: 00045182)

Entry	Reviewed	Entry Nam	Protein nar	Gene Nam	Organism	Mass
Q91WK2	reviewed	EIF3H_MO	Eukaryotic	Eif3h Eif3s	Mus muscı	39832

dent activity (GO: 0140657)

Entry	Reviewed	Entry Nam	Protein nar	Gene Nam	Organism	Mass
E9Q5G3	reviewed	KIF23_MOL	Kinesin-like	Kif23	Mus muscı	108776
Q09XV5	reviewed	CHD8_MO	Chromodo	Chd8 Kiaa:	Mus muscı	290847
Q3V0Q1	reviewed	DYH12_M(	Dynein axc	Dnah12 Dr	Mus muscı	356231
B1AVY7	reviewed	KI16B_MOI	Kinesin-like	Kif16b Kiaa	Mus muscı	150058
Q9JHS4	reviewed	CLPX_MOL	ATP-depen	Clpx	Mus muscı	69229
Q6ZPV2	reviewed	INO80_MC	Chromatin	Ino80 Inoc	Mus muscı	176520
E9QAM5	reviewed	HELZ2_MC	Helicase w	Helz2	Mus muscı	331563
Q8R420	reviewed	ABCA3_M(	Phospholip	Abca3	Mus muscı	191971
P21271	reviewed	MYO5B_M(	Unconvent	Myo5b Kia	Mus muscı	210570
E9Q876	reviewed	ABCAC_M(	Glucosylce	Abca12	Mus muscı	292592
Q8VDN2	reviewed	AT1A1_MO	Sodium/pc	Atp1a1	Mus muscı	112982
Q99PU8	reviewed	DHX30_M(	ATP-depen	Dhx30 Helg	Mus muscı	136668
Q5NC05	reviewed	TTF2_MOU	Transcripti	Ttf2	Mus muscı	125530
Q02053	reviewed	UBA1_MOL	Ubiquitin-l	Uba1 Sbx U	Mus muscı	117809
Q2KHI9	reviewed	MCM9_MC	DNA helica	Mcm9 Mcn	Mus muscı	125814
P41233	reviewed	ABCA1_M(	Phospholip	Abca1 Abc	Mus muscı	253912
Q6P9L6	reviewed	KIF15_MOL	Kinesin-like	Kif15 Klp2	Mus muscı	160120
Q6PGB8	reviewed	SMCA1_M(	Probable g	Smarca1 S	Mus muscı	121715

B2RR83	reviewed	YTDC2_MC	3'-5' RNA h	Ythdc2	Mus muscı	161092

ay feedback loops 2 (P04398) and p53 pathway (P00059)

Entry	Reviewed	Entry Nam	Protein nar	Gene Nam	Organism	Mass
Q61194	reviewed	P3C2A_MC	Phosphatic	Pik3c2a Cp	Mus muscı	190758
O88879	reviewed	APAF_MOL	Apoptotic p	Apaf1	Mus muscı	141003

: activity (GO: 0016209)

Entry	Reviewed	Entry Nam	Protein nar	Gene Nam	Organism	Mass
P20108	reviewed	PRDX3_MC	Thioredoxi	Prdx3 Aop1	Mus muscı	28127

tress response (P00046)

Entry	Reviewed	Entry Nam	Protein nar	Gene Nam	Organism	Mass
P0C871	reviewed	PA24B_MC	Cytosolic p	Pla2g4b	Mus muscı	88448

roteasome pathway (P00060)

Entry	Reviewed	Entry Nam	Protein nar	Gene Nam	Organism	Mass
Q02053	reviewed	UBA1_MOU	Ubiquitin-l	Uba1 Sbx U	Mus muscı	117809

(PC00072)

Entry	Reviewed	Entry Nam	Protein nar	Gene Nam	Organism	Mass
Q64433	reviewed	CH10_MOU	10 kDa hea	Hspe1	Mus muscı	10963
Q9R0Q7	reviewed	TEBP_MOL	Prostaglan	Ptges3 Sid	Mus muscı	18721
P17742	reviewed	PPIA_MOU	Peptidyl-pr	Ppia	Mus muscı	17971
P26883	reviewed	FKB1A_MC	Peptidyl-pr	Fkbp1a Fkl	Mus muscı	11923
