# Supplementary material for: Testicular SIRT1 Loss Reveals an Aging‐Like Proteomic Landscape and Precipitates Reproductive Deterioration
Source: Andrology. 2026 Mar 12;14(6):1579–89. doi: 10.1111/andr.70201 (PMC13432521; doi:10.1111/andr.70201)
Supplement: Supplementary file 4 — Supporting File 4: andr70201‐sup‐0004‐DataS3.pdf [file ANDR-14-1579-s005.pdf]

wt only; anti-aging proteins (*n*=5)		
**Entry**	**Protein**	**kDa**
P20108	Thioredoxin-dependent peroxide reductase, mitochondrial	28.1
P0C871	Cytosolic phospholipase A2 beta	88.5
Q02053	Ubiquitin-like modifier-activating enzyme 1	117.8
Q64433	10 kDa heat shock protein, mitochondrial	109.6
Q9R0Q7	Prostaglandin E synthase 3	187.2
P17742	Peptidyl-prolyl cis-trans isomerase A	179.7
P26883	Peptidyl-prolyl cis-trans isomerase FKBP1A	119.2
