# Supplementary material for: Testicular SIRT1 Loss Reveals an Aging‐Like Proteomic Landscape and Precipitates Reproductive Deterioration
Source: Andrology. 2026 Mar 12;14(6):1579–89. doi: 10.1111/andr.70201 (PMC13432521; doi:10.1111/andr.70201)
Supplement: Supplementary file 5 — Supporting File 5: andr70201‐sup‐0005‐DataS4.pdf [file ANDR-14-1579-s004.pdf]

wt only; regulators of spermatogenesis (*n*=15)		
**Entry**	**Protein**	**kDa**
Q8C0V1	Telomere repeats-binding bouquet formation protein 1	86.8
Q64511	DNA topoisomerase 2-beta	181.9
P60330	Separin	233.0
Q3TTP0	Testicular spindle-associated protein SHCBP1L	70.9
P70218	Mitogen-activated protein kinase kinase kinase kinase 1	91.5
Q4VA53	Sister chromatid cohesion protein PDS5 homolog B	164.4
Q8C0N0	Sperm motility kinase Z	56.1
Q920Q2	DNA repair protein REV1	137.3
Q811L6	Microtubule-associated serine/threonine-protein kinase 4	284.4
P05132	cAMP-dependent protein kinase catalytic subunit alpha	40.6
P11103	Poly [ADP-ribose] polymerase 1	113.1
O54692	Centromere/kinetochore protein zw10 homolog	88.1
P51830	Adenylate cyclase type 9	151.0
Q60991	Cytochrome P450 7B1	58.5
Q69ZA1	Cyclin-dependent kinase 13	164.6
