# Supplementary material for: Testicular SIRT1 Loss Reveals an Aging‐Like Proteomic Landscape and Precipitates Reproductive Deterioration
Source: Andrology. 2026 Mar 12;14(6):1579–89. doi: 10.1111/andr.70201 (PMC13432521; doi:10.1111/andr.70201)
Supplement: Supplementary file 6 — Supporting File 6: andr70201‐sup‐0006‐DataS5.pdf [file ANDR-14-1579-s001.pdf]

From	Entry	Reviewed	Entry Nam	Protein nar	Gene Nam	Organism	Length
CDKL5_MO	Q3UTQ8	reviewed	CDKL5_MO	Cyclin-dep	Cdkl5	Mus muscu	938
GLU2B_MO	O08795	reviewed	GLU2B_MO	Glucosidas	Prkcsh	Mus muscu	521
CTTB2_MO	B9EJA2	reviewed	CTTB2_MO	Cortactin-b	Cttnbp2 Ki	Mus muscu	1648
SMG1_MO	Q8BKX6	reviewed	SMG1_MO	Serine/thre	Smg1 Atx K	Mus muscu	3658
TCPE_MOU	P80316	reviewed	TCPE_MOU	T-complex	Cct5 Ccte	Mus muscu	541
MESD_MO	Q9ERE7	reviewed	MESD_MO	LRP chape	Mesd Meso	Mus muscu	224
GSTK1_MO	Q9DCM2	reviewed	GSTK1_MO	Glutathion	Gstk1	Mus muscu	226
PRDX6_MO	O08709	reviewed	PRDX6_MO	Peroxiredo	Prdx6 Aop2	Mus muscu	224
SRRM1_MO	Q52KI8	reviewed	SRRM1_MO	Serine/argi	Srrm1 Pop	Mus muscu	946
TMEDA_MO	Q9D1D4	reviewed	TMEDA_MO	Transmem	Tmed10 Tm	Mus muscu	219
RL7_MOUS	P14148	reviewed	RL7_MOUS	Large ribos	Rpl7	Mus muscu	270
TSGA8_MO	Q9JJL0	reviewed	TSGA8_MO	Testis-spec	Tsga8 Hala	Mus muscu	238
ZN292_MO	Q9Z2U2	reviewed	ZN292_MO	Zinc finger	Zfp292 Zfp	Mus muscu	2698
CAP1_MOU	P40124	reviewed	CAP1_MOU	Adenylyl cy	Cap1 Cap	Mus muscu	474
UBR5_MOU	Q80TP3	reviewed	UBR5_MOU	E3 ubiquiti	Ubr5 Edd E	Mus muscu	2792
STK10_MO	O55098	reviewed	STK10_MO	Serine/thre	Stk10 Lok	Mus muscu	966
EFTU_MOU	Q8BFR5	reviewed	EFTU_MOU	Elongation	Tufm	Mus muscu	452
S23IP_MOU	Q6NZC7	reviewed	S23IP_MOU	SEC23-inte	Sec23ip	Mus muscu	998
DBLOH_MO	Q9JIQ3	reviewed	DBLOH_MO	Diablo IAP-	Diablo Sma	Mus muscu	237
SETBP_MO	Q9Z180	reviewed	SETBP_MO	SET-bindin	Setbp1 Kia	Mus muscu	1582
ATM_MOUS	Q62388	reviewed	ATM_MOUS	Serine-pro	Atm	Mus muscu	3066
SDHA_MOU	Q8K2B3	reviewed	SDHA_MOU	Succinate	Sdha	Mus muscu	664
RYR2_MOU	E9Q401	reviewed	RYR2_MOU	Ryanodine	Ryr2	Mus muscu	4966
PHB2_MOU	O35129	reviewed	PHB2_MOU	Prohibitin-2	Phb2 Bap E	Mus muscu	299
ADT2_MOU	P51881	reviewed	ADT2_MOU	ADP/ATP tr	Slc25a5 Aa	Mus muscu	298
RB11A_MO	P62492	reviewed	RB11A_MO	Ras-relatec	Rab11a Ra	Mus muscu	216
LDHA_MOU	P06151	reviewed	LDHA_MOU	L-lactate d	Ldha Ldh-1	Mus muscu	332
ROP1_MOU	Q9ESG2	reviewed	ROP1_MOU	Ropporin-1	Ropn1	Mus muscu	212
PCNT_MOU	P48725	reviewed	PCNT_MOU	Pericentrin	Pcnt Pcnt2	Mus muscu	2898
